# MuSyC is a consensus framework that unifies multi-drug synergy metrics for combinatorial drug discovery

**DOI:** 10.1038/s41467-021-24789-z

**Published:** 2021-07-29

**Authors:** David J. Wooten, Christian T. Meyer, Alexander L. R. Lubbock, Vito Quaranta, Carlos F. Lopez

**Affiliations:** 1 Department of Physics, Pennsylvania State University, University Park, PA USA; 2 Program in Chemical and Physical Biology, Vanderbilt University School of Medicine, Nashville, TN USA; 3 Department of Biochemistry, Vanderbilt University Nashville, Nashville, TN USA; 4 Department of Pharmacology, Vanderbilt University School of Medicine, Nashville, TN USA; 5 Department of Biomedical Informatics, Vanderbilt University Medical Center, Nashville, TN USA

**Keywords:** Combinatorial libraries, Systems biology, Computer modelling, Standardization

## Abstract

Drug combination discovery depends on reliable synergy metrics but no consensus exists on the correct synergy criterion to characterize combined interactions. The fragmented state of the field confounds analysis, impedes reproducibility, and delays clinical translation of potential combination treatments. Here we present a mass-action based formalism to quantify synergy. With this formalism, we clarify the relationship between the dominant drug synergy principles, and present a mapping of commonly used frameworks onto a unified synergy landscape. From this, we show how biases emerge due to intrinsic assumptions which hinder their broad applicability and impact the interpretation of synergy in discovery efforts. Specifically, we describe how traditional metrics mask consequential synergistic interactions, and contain biases dependent on the Hill-slope and maximal effect of single-drugs. We show how these biases systematically impact synergy classification in large combination screens, potentially misleading discovery efforts. Thus the proposed formalism can provide a consistent, unbiased interpretation of drug synergy, and accelerate the translatability of synergy studies.

## Abstract (web summary)

The lack of a unifying metric characterizing combinatorial drug interactions has impeded the development of combinatorial therapies. Here, the authors present MuSyC, a consensus synergy metric that overcomes several caveats associated with other, popular metrics.

## Introduction

Throughout the preceding century, two principles have been used to quantify synergy of drug combinations: the Dose Equivalence Principle (DEP), introduced by Loewe^1,2^, and the Multiplicative Survival Principle (MSP), introduced by Bliss^3^. In 1992, a committee was convened in Saariselkä, Finland seeking to find a consensus between these principles and unify the field^4,5^. Unable to reconcile their differences, the committee’s conclusion (The Saariselkä Agreement) did not reach a consensus and simply recommended that drug combination studies explicitly state how synergy was calculated^4,5^. Multiple synergy models have since emerged, often derived as extensions of either the DEP or MSP, further splintering the field^6–12^. In the absence of a consensus framework for drug synergy, discovery efforts for combinations often calculate all available synergy metrics^13–15^, as first recommended by Greco and colleagues following Saariselkä^16^. However, there remains no basis for choosing one metric over another, which becomes particularly problematic when synergy metrics conflict. This “calculate everything” paradigm thus hampers reproducibility between studies, delays progress in the discovery of synergistic drug combinations, and negatively impacts the translatability of combination discovery efforts.

Despite the lack of consensus on how to quantify synergy, drug combination screens remain essential to both pharmaceutical and academic discovery efforts, as shown by recent studies by AstraZeneca and the NCI-DREAM consortia^17,18^, as well as combinatorial CRISPR screens^19^. Yet, the paucity of successful clinical combinations explicable by true pharmacological interaction, rather than patient-to-patient variability^20^, is symptomatic of the challenges facing the field. Therefore, the need identified at Saariselkä still exists: a consensus framework to interpret drug combination pharmacology.

We recently introduced a framework to quantify synergy based on the Law of Mass Action, named Multi-dimensional Synergy of Combinations (MuSyC)^21^, that distinguishes between different synergy types (e.g., potency, efficacy). In the present work, we build upon our previous findings to show how MuSyC generalizes the DEP and MSP, thereby unifying the field of drug synergy, as sought at Saariselkä. Further, we map the landscape of current synergy metrics, including: Bliss Independence^3^, Loewe Additivity^1^, Combination Index (CI)^22^, Highest Single Agent (HSA)^23^, Effective Dose model^6^, ZIP^7^, a partial differential equation (PDE) Hill model by Schindler^8^, BRAID^11^, and the General Pharmacodynamic Interaction (GPDI) model^24^. In mapping relationships between these various frameworks, we identified systematic differences impacting the interpretation of synergy in drug combination experiments. Specifically, we found: (1) the conflation of synergistic potency and efficacy masks synergistic interactions; (2) MSP-based frameworks are biased toward antagonism for drugs with intermediate efficacy; and (3) DEP-based frameworks contain a Hill-slope dependent bias. The Hill-slope bias results from satisfying the famous “sham” combination thought experiment, thus arguing against the merit of sham-compliance as a measure of validity for synergy frameworks. Using five large combination datasets^25–29^, MuSyC identifies real-world examples where the conflicting assumptions of previous drug synergy frameworks misleads or impedes drug discovery efforts through these pervasive and predictable biases. Additionally, we show that MuSyC uncovers two consequential errors in the highly cited CI^22,30^ which has been proposed as the standard Mass Action-based, synergy framework^31^. We therefore propose MuSyC as a consensus framework to interpret combination pharmacology and signify its broad applicability to the study of drug mixtures.

## Results

### A state-transition model to measure multi-drug synergistic effects

**Fig. 1:**
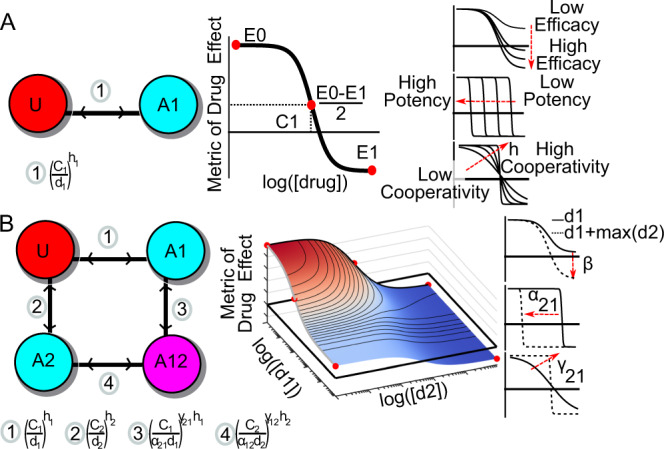
MuSyC is a mass-action, state-transition model of drug combination synergy. **A** Two-state transition model for a single drug system. The “unaffected” cells are indicated in red with the letter “U”, while affected cells are indicated in cyan with “A1”. The traditional equation for fitting dose-response relationships (middle) is the 4-parameter Hill equation. We derive this equation using the Law of Mass Action from a two-state transition model of drug effect (left). Edge notation is equal to the ratio of states' percent occupancy at equilibrium ($\frac{{A}_{1}}{U}$) at dose (*d*_1_). The Hill equation contains parameters measuring a drug’s efficacy (*E*_0_ − *E*_1_), potency (*C*), and cooperativity (*h*). Each parameter corresponds to distinct geometric transformations of the dose-response curve (right). **B** Two-drug model: MuSyC is derived from a four-state state-transition model of combination pharmacology (left) based on the Law of Mass Action and results in a 2D Hill-like equation describing a dose-response surface (middle). Cells affected by both drugs are indicated in the magenta circle and labeled “A12”. Red to blue color gradient on the dose-response surface ranges from no effect (red) to maximum achieved effect (blue). The edge notations (left) refer to the ratio of the connected corners for the boundary condition. For example, edge #3 annotation means $\left(\frac{{A}_{12}}{{A}_{1}}\right)\to \left({\frac{{C}_{1}}{{\alpha }_{21}{d}_{1}}}^{{\gamma }_{21}{h}_{1}}\right)$ when *d*_2_ → *∞*. Beyond the parameters of the single Hill equation, the 2D Hill equation has additional parameters (*β*, *α*, *γ*) corresponding to distinct transformations of the dose-response surface (right) (Video S1). These transformations describe changes in a single drug’s efficacy, potency, and cooperativity due to the combination, and, therefore, are interpreted as synergistic efficacy (*β*), synergistic potency (*α*), and synergistic cooperativity (*γ*). There are two values for *α* and *γ* because each drug can independently modulate the potency and cooperativity of the other^6,7^ (edge 3 vs. edge 4 of the state transition model). In contrast, the single *β* parameter describes the percent increase in maximal effect due to both drugs (effect *E*3 at *A*12). See Fig. S1 for MuSyC extension to three drugs.

The 4-parameter Hill equation is commonly used to fit dose-response data from in vitro and in vivo assays (see Box 1 Eq. (10) and Table 1 for parameter annotation). Here we derive this equation from the equilibrium of a two-state transition model of drug effect based on the Law of Mass Action (Fig. 1A, left). Traditionally, the parameters of the Hill equation are interpreted as a drug’s efficacy (*E*_0_ − *E*_1_), potency (*C*), and cooperativity (*h*), also known as the Hill slope. These parameters correspond to three possible geometric transformations of a dose-response curve (Fig. 1A, right). To generalize this one-drug formalism to two concurrent drugs, we developed a four-state transition model of combination pharmacology (Fig. 1B, left)^21^. From this model, we derive a two-dimensional (2D) Hill equation for two drugs (Box 1, Eq. (15)) defining a dose-response surface (Fig. 1B, middle). The 2D Hill equation contains five additional parameters, not present in the single-drug Hill equation, which measure different types of drug interactions. These additional parameters measure changes in a drug’s efficacy (*β*), potency (*α*_12_ and *α*_21_), and cooperativity (*γ*_12_ and *γ*_21_) in a combination—corresponding to three distinct types of synergy (Fig. 1B, right, Table 1). See Supplemental Code 1 and Supplemental Section Interactive MuSyC Jupyter Notebook for an interactive demonstration of the 2D Hill equation parameters. As we show below, these parameters are conflated in traditional synergy metrics (e.g. Loewe, Bliss, and HSA), as well as in recently proposed ones obscuring the true origin and magnitude of drug synergy or antagonism.

**Table 1:** Annotation of MuSyC parameters.

*U*	Percent of unaffected population.
*A*_1_, *A*_2_	Percent of affected by drug 1 and drug 2, respectively.
*A*_1,2_	Percent of affected by both drug 1 and drug 2.
*d*_1_, *d*_2_	Drug concentrations for drug pair.
*E*_*d*_	Measured effect at (*d*_1_, *d*_2_).
*C*_1_, *C*_2_	The concentration of drug required to achieve 50% of the maximal effect (i.e., EC_50_).
*h*_1_, *h*_2_	Hill coefficients for dose-response curves of drug 1 and 2 in isolation.
*E*_0_	The basal effect *E*_*d*_ (*d*1 = *d*2 = 0).
*E*_1_, *E*_2_	Maximal efficacy of drugs 1 and 2 in isolation.
*E*_3_	Maximal efficacy of the combination of drugs 1 and 2.
*β*	Percent increase (or decrease) in max effect with both drugs over the most efficacious single drug ($\beta := \frac{\min ({E}_{1},{E}_{2})-{E}_{3}}{{E}_{0}-\min ({E}_{1},{E}_{2})}$).
*α*_12_	Fold change in the potency (*C*_2_) of [*d*_2_] induced by drug 1.
*α*_21_	Fold change in the potency (*C*_1_) of [*d*_1_] induced by drug 2.
*γ*_12_	Fold change in the cooperativity (*h*_2_) of [*d*_2_] induced by drug 1.
*γ*_21_	Fold change in the cooperativity (*h*_1_) of [*d*_1_] induced by drug 2.

### Mapping the landscape of prominent synergy models within a consensus framework

**Fig. 2:**
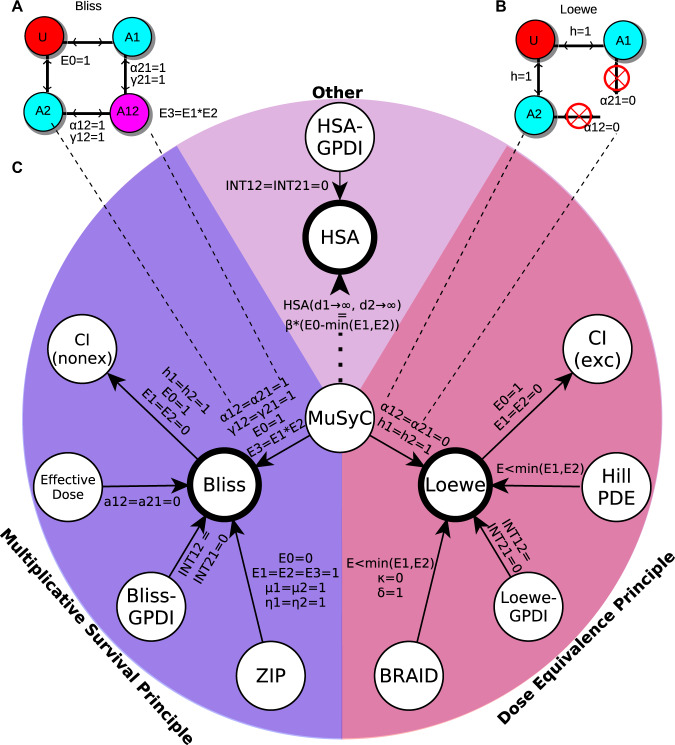
Unifying MSP and DEP with MuSyC, and mapping the landscape of drug synergy. **A** The Bliss null model, the base model for all MSP frameworks, emerges from MuSyC when *E*_0_ = 1, *α*_12_ = *α*_21_ = *γ*_12_ = *γ*_21_ = 1, and *E*_3_ = *E*_1_*E*_2_. **B** The Loewe null model, the base model for all DEP frameworks, emerges from MuSyC when *h*_1_ = *h*_2_ = 1 and *α*_12_ = *α*_21_ = 0. The constraint on *α* indicates the drugs' activities are mutually exclusive (i.e., the double-drugged state *A*_1,2_ does not exist). **C** Network of relationships between synergy frameworks (nodes) grouped by their underlying principle (colors). The notation next to solid edges signifies conditions under which source model reduces to end model’s null model. The dotted edge indicates MuSyC synergistic efficacy (*β*) is proportional to HSA as *d*_1_ → *∞*, *d*_2_ → *∞*. See Supplemental Section Derivation of the theoretical relationships between different synergy frameworks for complete annotation of the parameters defined in each method. Where possible, parameters from each framework were translated in terms of the dose-response parameters defined for MuSyC (Table 1) to facilitate comparison. CI has two equations for synergy for the mutually exclusive (exc) and non-mutually exclusive (nonex) binding case. Conditions for which Bliss or Loewe reduces to the CI(nonex) and CI(ex) models were found in Chou and Talalay^34^. The mutually exclusive case is recommended by Chou^37^ and used in the CompuSyn software. Likewise, GPDI has three separate equations for HSA, Bliss, and Loewe models. In contrast, MuSyC has one equation (Box 1, Eq. (15)) which reduces to Bliss and Loewe under certain conditions.

Multiple alternative synergy models have been proposed, most broadly derived from one of two guiding principles: the Multiplicative Survival Principle (MSP) or the Drug Equivalence Principle (DEP) (Table 2). Prior work has shown contradictory results when comparing between MSP and DEP frameworks^4,12,32^, and a lack of consensus remains on the commonality between the two principles^4,7,9,11^. Here we show MuSyC satisfies both the DEP and MSP under specific parametric constraints (Fig. 2A, B), thereby unifying the foundational principles of drug synergy.

**Table 2:** Comparison of traditional and modern frameworks for calculating synergy. *CI has two equations for synergy in the original derivation^22^, for the mutually exclusive and mutually non-exclusive case. The mutually exclusive case has been widely adopted and is the model compared here. The concentration-dependent synergy of Bliss and Loewe are explored in Supplemental Section “Interactive MuSyC Jupter Notebook”, Figs. S12–S16, and Supplemental Code 1, an interactive Jupyter Notebook. This notebook shows how different MuSyC synergy parameters may be reflected at specific concentrations of Bliss or Loewe synergy.

	Dose Equivalence Principle					Multiplicative Survival Principle				Other		Multiple
	Loewe	BRAID	Hill PDE	GPDI (Loewe)	Combination Index*	Bliss	Effective Dose	ZIP	GPDI (Bliss)	HSA	GPDI (HSA)	MuSyC
Defined for arbitrary metrics of drug effect (not just percent data)	✓	✓	–	✓	–	–	–	✓		✓	✓	✓
Does not require all drugs to be equally efficacious	–	✓	✓	✓	–	✓	–	–	✓	✓	✓	✓
Synergy is concentration independent	–	✓	–	–	–	–	✓	–	–	–	–	✓
Synergy is related to traditional dose-response parameters	–	–	–	✓	–	–	✓	–	✓	✓	✓	✓
Satisfies the sham experiment	✓	✓	✓	✓	✓	–	–	–	–	–	–	–

The MSP was first described by Bliss^3^ and is the foundation of the Bliss Independence framework. MSP assumes the probability of a cell surviving treatment by drug 1 (*U*_1_) is independent of the probability of the same cell surviving treatment by drug 2 (*U*_2_). Therefore, the probability of surviving both Drug 1 and Drug 2 is *U* = *U*_1_ ⋅ *U*_2_^3^. Synergy or antagonism occur when *U* ≠ *U*_1_ ⋅ *U*_2_. A method to define an alternative Bliss null model has been reported for growth-rate data as the sum of growth-rate inhibition^33^, but this formulation is uncommon, and not classified as MSP. MuSyC satisfies the MSP under the following conditions: (1) the effect metric is expressed as a percent (*E*_0_ = 1, and *E*_3_ = *E*_1_*E*_2_), (2) there is no potency synergy (*α*_12_ = *α*_21_ = 1), and (3) there is and no cooperativity synergy (*γ*_12_ = *γ*_21_ = 1) (Fig. 2A, see Supplemental Section Multiplicative Survival Principle for details).

The DEP was first established by Loewe^1,2^. DEP-based methods are characterized by linear isoboles (contours of equal effect) (Fig. S2A). A combination of doses *d*_1_ and *d*_2_, achieving effect *E*, satisfies the DEP when $\frac{{f}_{1}({d}_{1})}{{f}_{1}^{-1}(E)}+\frac{{f}_{2}({d}_{2})}{{f}_{2}^{-1}(E)}=1$, where *f*_*i*_(*d*_*i*_) represents the monotherapy response of drug *i*. MuSyC satisfies the DEP under the following conditions: (1) the drugs’ actions are mutually exclusive (*α*_12_ = *α*_21_ = 0) and (2) *h*_1_ = *h*_2_ = 1 (Figs. 2B and S2B, see Supplemental Section Dose Equivalence Principle for details).

From the literature, we identified several prominent synergy models beyond Bliss and Loewe including: CI^34^, HSA^23^, Effective Dose model^6^, ZIP^7^, Hill PDE^8^, and GPDI^24^. Table 2 compares key features and assumption of the different synergy models. Each of these methods, as well as MuSyC, defines synergy based on the experimental deviation from a null (additive) dose-response surface. Because almost all synergy frameworks are founded on either the DEP or MSP, we standardized relationships between the various models, mapping the global landscape of drug synergy (Fig. 2C, see Supplemental Section Relationships between different synergy frameworks for details).

**Fig. 3:**
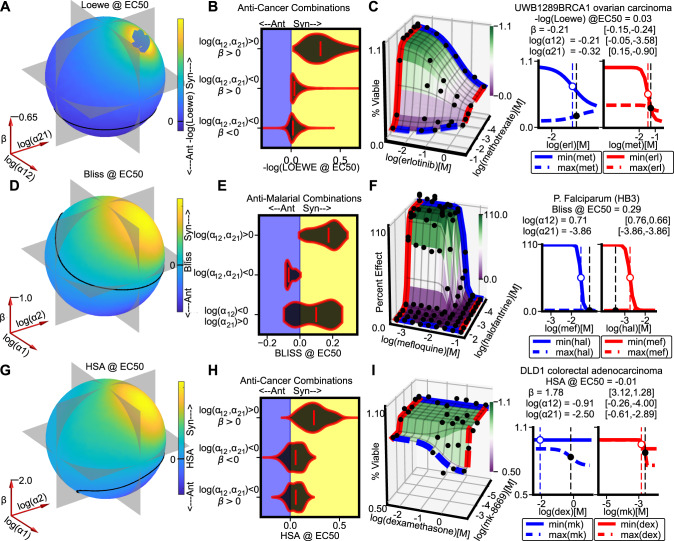
Conflating potency and efficacy synergy masks synergistic interactions in large drug combination datasets. **A** The colors on the sphere (radius on *β* axis bottom left) represent the value of Loewe (colorbar to right) for a drug combination with a MuSyC synergy profile (*α*_12_, *α*_21_, and *β*) (axes bottom left). For all combinations: *E*_0_ = 1, *E*_1_ = *E*_2_ = 0, *h*_1_ = *h*_2_ = 1, *d*_1_ = *d*_2_ = *C*_1_ = *C*_2_, *γ*_12_ = *γ*_21_ = 1. The solid line marks the boundary between Loewe synergy and antagonism. Along this contour, which includes many different sets of (*α*_12_, *α*_21_, and *β*), Loewe is the same (−log(Loewe) = 0). Gray planes correspond to *β* = 0, ${{{\mathrm{log}}}}\,({\alpha }_{12})=0$, and ${{{\mathrm{log}}}}\,({\alpha }_{21})=0$. The hole in the upper-right quadrant represents sets for which Loewe is undefined. **B** Distribution of Loewe for anti-cancer drug combinations^26^ grouped by their synergy profiles according to MuSyC. Loewe was calculated as detailed in Methods section, including the Hill slope correction. The background color distinguishes antagonism (purple, “Ant”) from synergism (yellow, “Syn”). **C** The anti-cancer combination methotrexate and L-778123^26^ is antagonistically potent and efficacious against HT29 cells, by MuSyC; however, it is designated by Loewe to be synergistic. Left panel shows the MuSyC-fitted dose-response surface, right panel shows the edges of the MuSyC surface. Color on the dose-response surface indicates effect (% Viable), with colorbar given next to the surface. On the right, the open circles mark the EC50 for each drug in isolation, closed circle is the shifted EC50 due to antagonistic potency. Brackets are 95% confidence intervals for each parameter based on Monte Carlo sampling (see Methods section). **D** Sphere for Bliss as in **A**. **E** Distribution of Bliss for anti-malarial drug combinations^25^. Combinations for which each drug alone achieves *E*_*max*_ < 0.1 were selected, ensuring *E*_1_ ⋅ *E*_2_ ≈ *E*_3_ ≈ 0. Under this condition, the differences between MuSyC and Bliss are due only to asymmetric potency synergy (all combinations near the *β* = 0 plane in **D**). **F** Mefloquine increases the potency of halofantrine (red curves) but halofantrine decreases the potency of mefloquine (blue curves) in the HB3 strain of *P. falciparum*^25^. **G** Sphere for HSA as in **A**. **H** Distribution of HSA for anti-cancer combinations^26^ grouped by MuSyC synergy profile. In antagonistically potent combinations, HSA can miss synergistic efficacy. **I** Combination of dexamethasone and mk-8669 in DLD1 cells^26^ is anagonistically potent, but synergistically efficacious.

**Fig. 4:**
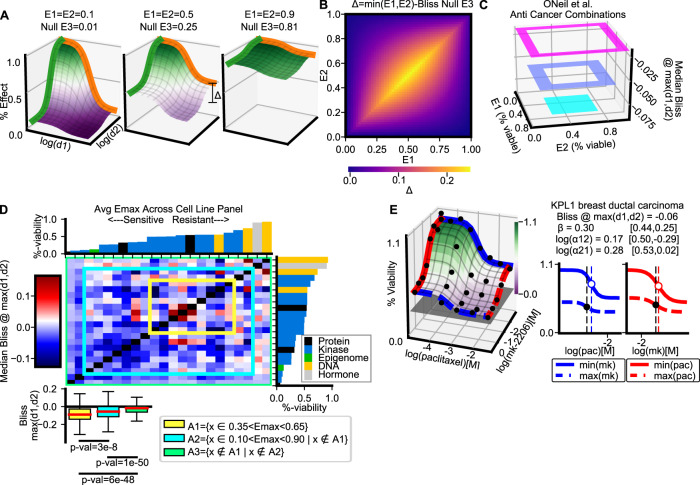
Bliss is biased against combinations of moderately efficacious drugs. **A** The null dose-response surface according to Bliss such that Bliss is zero at all doses for different single agent efficacy. Δ is defined as the expected increase in percent effect of the combination over the stronger single agent at saturating doses. The left and right panels have the same expected increase according to Bliss, Δ = 0.09, while the combination of moderately efficacious drugs (middle panel) has a expected increase of Δ = 0.25. The color of the surface indicates the drug combination effect, from no effect (green) to maximum (purple). The solid color lines on the left and back sides show the single-drug responses. **B** Calculation of Δ (colorbar bottom) for surfaces with different pairings of (*E*1, *E*2). Color indicates the difference, with range given on the colorbar below the image. **C** Median Bliss for anti-cancer combinations^26^ grouped by the maximal efficacy of their single agents. Ranges for each square: cyan square: [0.35, 0.65], blue square: [0.1, 0.9], and magenta square: [0.0, 1.0]. Bliss is calculated at the maximum tested concentrations of both drugs. **D** Heatmap of the median Bliss score (colorbar left) for each combination across the cancer cell-line panel^26^. Rows and columns are ordered by the average efficacy of each drug alone over all cell-lines (*E*_*max*_) (bar graphs top and right). Colored boxes correspond to groupings denoted in the legend (bottom). Boxplots show Bliss trends toward antagonism for combinations of moderately efficacious drugs (assuming approximate normality, one-sided *t*-test, blue < yellow;green < blue;green < yellow). The bottom and top of the boxes are the first and third quartiles, respectively. The red line shows the median. The whiskers extend to 1.5 times the interquartile range below and above the first and third quartiles. The boxplots represent *n* = 967 biologically independent combinations (yellow), *n* = 6047 combinations (cyan), and *n* = 7773 combinations (green). **E** Dose-response surface of paclitaxel and mk-2206 in KPL1 cells^26^. Gray plane is the expected effect of the combination by Bliss at max(d1,d2).

**Fig. 5:**
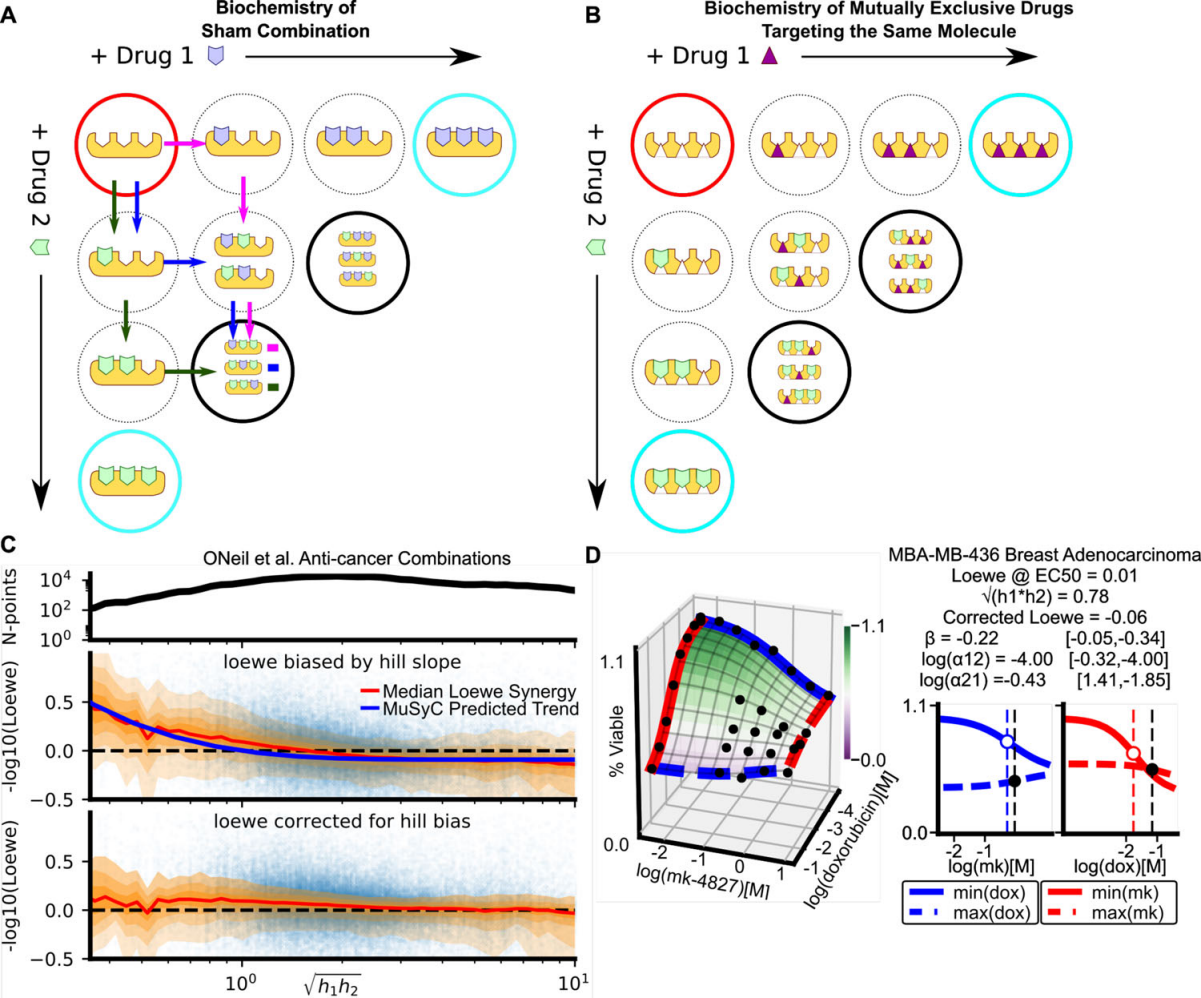
Hill-slope dependent bias results from enforcing sham compliance DEP-based frameworks. **A** An illustration of the unique biochemistry of the sham experiment. The red circle represents an undrugged molecule with three binding sites. In a sham experiment, a drug is treated as though it were two separate drugs (green and blue polygons). Mixed states in which the binding sites are bound by both green and blue drugs (black circles) are equivalent to fully drugged states (cyan circles). We highlight three paths (green, blue, magenta arrows) that can be followed to reach a mixed-drugged state. These three paths correspond to the coefficient of $3\frac{{d}_{1}{d}_{2}^{2}}{{C}^{3}}$ in Eq. (19) in Box 2. **B** In a combination of mutually exclusive drugs (triangle and polygon), targeting the same molecule, and with the same number of binding sites, the mixed states (black circles) are not equivalent to fully drugged (cyan circles) accounting for the discrepancy between MuSyC and the sham experiment (Box 2). **C** Loewe synergy is biased by Hill slope in the anti-cancer drug screen^26^. The orange shaded regions show moving window percentiles (window width is 0.1) of Loewe (10th through 90th percentiles, in steps of 10). The top panel shows how many data points are present in the window. The blue curve in the middle plot shows the median MuSyC-predicted bias as a function of the geometric mean of the Hill slopes (see Methods section). Subtracting the MuSyC-estimated bias (calculated for each data point) from Loewe yields the bottom plot. **D** The antagonistically efficacious and potent combination of mk-4827 and doxorubicin^26^ is misidentified as synergistic by Loewe, because both drugs in isolation have Hill slopes *h* < 1.

In deriving this map, we uncovered potential sources of error when using MSP or DEP methods which impact interpretation of synergy studies. Specifically, we identified three recurrent considerations meriting attention from the field. (1) Previous synergy metrics conflate different synergy types (i.e. potency, efficacy, cooperativity) in ways that can mask synergistic and antagonistic interactions (Fig. 3). (2) The connection between MuSyC and the MSP-derived frameworks depend on the single drugs’ efficacy (*E*_1_, *E*_2_), and as a result, MSP frameworks are biased against the combination of moderately efficacious single agents (Fig. 4). (3) The connection between the DEP and MuSyC is constrained by single drugs’ Hill slopes (*h*), and therefore DEP frameworks impose a Hill-slope dependent bias, artificially inflating the synergy for drugs with low Hill slopes (Fig. 5). This bias is a consequence of satisfying the sham experiment as described in Section Re-examining the sham experiment: Sham compliance introduces Hill-dependent bias in DEP models. While there may be different approaches to quantify synergy relative to a given null model, the biases we discuss are intrinsic to the null models themselves, while the magnitude of bias depends on the precise synergy quantification. To assess impact of these considerations on synergy calculations in different fields, we analyzed five large publicly available datasets (Table 3) using MuSyC and other synergy frameworks (see Methods section for description of fitting methods). We find the MuSyC algorithm to be robust to different types and magnitude of noise as well as sampling designs facilitating the analysis of many types of data (Fig. S3). Note, while synergistic cooperativity (*γ*) is theoretically plausible (as initially postulated by^7^), including it did not increase the fit quality (Fig. S4) as measured by AIC and therefore we do not explore synergistic cooperativity in subsequent analyses.

**Table 3:** Summary of the datasets used for comparisons and validating theoretical predictions by MuSyC.

Model	# of Combinations	Metric of drug effect	Effect range	Refs.
*P. falciparum* (Strains:3D7,HB3,Dd2)	773	Percent response	[0,100]	^25^
37 cancer cell lines	22,738	Percent viable	[0,1]	^26^
60 cancer cell lines	330,064	Percent growth	[−100, 100]	^27^
HIV	116	Infectivity	[0,1]	^28^
*S. cerevisiae*	175	Area under growth curve	[0,484]	^29^

### Conflating synergistic potency and efficacy masks synergistic interactions

To determine how conflation of distinct synergy types impacts the interpretation of drug-response data, we generated synthetic dose-response surfaces using MuSyC (Eq. (15)) across a range of *α* and *β* values and calculated the synergy according to Loewe, Bliss, and Highest Single Agent (HSA) at the EC50 of both drugs (Fig. 3A, D, G and Video S2). In each case, many distinct sets of (*α*_12_, *α*_21_, *β*) are indistinguishable (e.g. the black contour line on the spheres).

Figure 3A shows that near the boundary between synergism and antagonism, Loewe is insensitive to changes in synergistic potency, tracking instead with synergistic efficacy. Consequently, in the anti-cancer dataset from O’Neil et al.^26^, Loewe misses potency antagonism in combinations with synergistic efficacy (Fig. 3B middle distribution, see Fig. S5 for an example surface). This reflects Loewe’s assertion of infinite potency antagonism (*α*_12_ = *α*_21_ = 0, Fig. 2A) in its null model. Therefore, combinations that are antagonistically potent (*α* < 1) are all synergistic by Loewe in the absence of sufficient antagonistic efficacy (values above black contour in Fig. 3A). Indeed, Loewe is frequently synergistic even in cases of antagonistic potency and efficacy (Fig. 3B bottom distribution). As an example, the combination of methotrexate (targets folate synthesis) and erlotinib (EGFR inhibitor) in UWB1289 (BRCA1-mutant ovarian carcinoma) cells is antagonistically efficacious and potent by MuSyC, but synergistic by Loewe (Fig. 3C).

Some assays, such as the anti-malarial combination screen from Mott et al.^25^ are designed so that the drug effect spans the entire range from *E*_0_ = 1 to *E*1 = *E*2 = *E*3 = 0. In such cases there is no synergistic efficacy, because each drug alone already achieves the greatest efficacy measurable. Nevertheless, even if all synergy detected by an assay is synergistic potency, traditional synergy metrics can still conflate asymmetric synergistic potencies. This can occur when *α*_12_ is synergistic while *α*_21_ is antagonistic, or vice versa (Fig. 3D, Bliss synergy, black contour line through *β* = 0 plane). In the anti-malarial dataset^25^, Bliss is consistently synergistic when *log*(*α*_12_, *α*_21_) > 0, and antagonistic if *log*(*α*_12_, *α*_21_) < 0; however, when *log*(*α*_12_) < 0 < *log*(*α*_21_), Bliss will strictly classify a combination as either synergistic or antagonistic (Fig. 3E bottom distribution) despite the asymmetric interactions. As an example, Bliss conceals that halofantrine (inhibits polymerization of heme molecules) reduces the potency of mefloquine (targets phospholipids) against the multi-drug resistant malaria strain HB3 (Fig. 3F).

HSA is commonly thought to quantify synergistic efficacy. However, for antagonistically potent combinations, HSA cannot distinguish synergistic and antagonistic efficacy because it does not account for the topology of the dose-response surface (compare $({{{\mathrm{log}}}}\,({\alpha }_{12}),{{{\mathrm{log}}}}\,({\alpha }_{21}),\beta )=(-,-,+)$ and ( − , − , − ) quadrants of Fig. 3G and Video S2). In the anti-cancer combination dataset^26^, we observe this trend (Fig. 3H middle vs bottom distributions). As an example, the synergistically efficacious combination of dexamethasone (agonist of the glucocorticoid receptor) and mk-8669 (PI3K/mTOR dual inhibitor) in a colorectal adenocarcinoma cell-line is masked by HSA due to antagonistic potency (Fig. 3I). Repressing glucocorticoid signaling has previously been shown to repress mTOR signaling^35^ providing a potential molecular mechanism explaining the synergy.

### MSP is biased against combinations of drugs with intermediate efficacy

MSP frameworks, such as Bliss, explicitly expect drug effects to measure the “percentage of cells affected”, which is by definition bounded within the closed interval *E* ∈ [0, 1]. Nevertheless, dose-response data is usually not a measure of percent affect, but rather of relative percent effect (see Supplemental Section Percent Affect vs Percent Effect for an example). This distinction, maintained by MuSyC (Box 1), is critical because percent effect data commonly saturates (i.e., percent affect is near 100%) at intermediate effect (i.e., relative percent effect is near 50%). For combinations of these moderately efficacious drugs, Bliss expects a large increase in effect over the single agents, even when each drug is administered at saturating concentrations (Fig. 4A, middle panel). In contrast, if combining drugs with high or low efficacy, Bliss expects a more modest increase (Fig. 4A, left and right panels).

Based on this expectation that *E*_3_ = *E*_1_ ⋅ *E*_2_ (Fig. 2A), MuSyC predicts Bliss would be biased toward antagonism in combinations of moderately efficacious drugs (Fig. 4B yellow shading around *E*_1_ = *E*_2_ ≈ 0.5). As expected, the median Bliss score in the anti-cancer dataset is biased toward antagonism for moderately efficacious combinations 0.35 < (*E*1, *E*2) < 0.65 (Fig. 4C, cyan square), though the magnitude of bias is less than predicted in Fig. 4B. This bias persists even when looking at pan-cancer trends in the combination of drugs which have, on average, intermediate effect over the entire cell-line panel (Fig. 4D). As a particular example, the synergistic efficacy of paclitaxel (targets microtubule stability) and mk-2206 (AKT inhibitor) in KPL1 cells is masked by Bliss’s high expectation for moderately efficacious drugs (Fig. 4E, gray plane). In several datasets, the magnitude of this MuSyC-predicted bias was sufficient to obfuscate many of the strongest synergies and antagonisms according to Bliss (Fig. S6A, B).

Other MSP-based methods, such the Effective Dose model also assume data measures percent affect and fit a simplified 2-parameter Hill equation enforcing *E*_0_ = 1 and *E*_1_ = 0. This assumption can lead to poor fits of percent effect data for moderately efficacious drugs, and thus invalid synergy scores (Fig. S7). Therefore, the distinction between percent affect and percent effect is a critical component of MuSyC.

Box 1 Derivation of MuSyC Consider a reversible transition between an unaffected population (*U*) and an affected population (*A*) governed by

7 $$U\mathop{\rightleftharpoons}\limits_{{r}_{-1}}^{{r}_{1}\cdot {d}^{h}}A$$

where *d* is the concentration of the drug, *h* is the Hill slope, often called cooperativity, and *r*_1_ and *r*_−1_ are constants corresponding to the reaction rate (Fig. 1A). Applying the Law of Mass Action, steady state ratios of *U* and *A* are

8 $$\begin{array}{l}\frac{{{{\rm{d}}}}U}{{{{\rm{d}}}}t}=A\cdot {r}_{-1}-U\cdot {r}_{1}{d}^{h}\equiv 0\\ \frac{A}{U}=\frac{{r}_{1}{d}^{h}}{{r}_{-1}}\equiv {\left(\frac{d}{C}\right)}^{h}\end{array}$$

When $d={\left(\frac{{r}_{-1}}{{r}_{1}}\right)}^{\frac{1}{h}}$, then (*A* = *U*). This dose is commonly called the EC50 (herein denoted as *C*). Equation (8) is called the “median effect equation”, and has been shown to describe multiple distinct drug mechanisms of action^52^. Because 100% of the population is either unaffected or affected, we also have the condition *U* + *A* = 1. This leads to the 2-parameter 1D Hill equation

9 $$U=\frac{{C}^{h}}{{C}^{h}+{d}^{h}}=\frac{1}{1+{\left(\frac{d}{C}\right)}^{h}}$$

If the *U* and *A* differ by an observed effect (such as proliferation rate^53^), the measured effect *E* at dose *d* will be a weighted average

$$E=U\cdot {E}_{0}+A\cdot {E}_{1},$$

where *E*_0_ and *E*_1_ are the the effects characteristic of the *U* and *A*, respectively. From this we find the final form of a 4-parameter Hill equation:

10 $$E={E}_{1}+\frac{{E}_{0}-{E}_{1}}{1+{\left(\frac{d}{C}\right)}^{h}}$$

**2D extension of the Hill equation for two-drug systems**.

Consider a system with 4 possible states, *U*, *A*_1_, *A*_2_, and *A*_1,2_ corresponding to populations that are unaffected, affected by drug 1 alone, affected by drug 2 alone, or affected by both drugs, respectively. The corresponding transitions between these states are:

11 $$\left[[U\mathop{\rightleftharpoons}\limits_{{r}_{-1}}^{{r}_{1}\cdot {d}_{1}^{{h}_{1}}}{A}_{1};\quad U\mathop{\rightleftharpoons}\limits_{{r}_{-2}}^{{r}_{2}\cdot {d}_{2}^{{h}_{2}}}{A}_{2};\quad {A}_{1}\mathop{\rightleftharpoons }\limits_{{r}_{-2}^{{\gamma }_{12}}}^{{r}_{2}^{{\gamma }_{12}}\cdot {\left({\alpha }_{12}{d}_{2}\right)}^{{\gamma }_{12}{h}_{2}}}{A}_{1,2};\quad {A}_{2}\mathop{\rightleftharpoons}\limits_{{r}_{-1}^{{\gamma }_{21}}}^{{r}_{1}^{{\gamma }_{21}}\cdot {\left({\alpha }_{21}{d}_{1}\right)}^{{\gamma }_{21}{h}_{1}}}{A}_{1,2}]\right]$$

Here, the *α* parameters quantify the modulation of one drug’s EC50 (potency) due to the other drug. Similarly, the *γ* parameters measure the change of a drug’s Hill slope (cooperativity) due to the other drug.

As in the 1D case, finding the steady state of the system leads to the following system of equations

12 $$\frac{{{{\rm{d}}}}U}{{{{\rm{d}}}}t}	=-U\cdot \left({r}_{1}{d}_{1}^{{h}_{1}}+{r}_{2}{d}_{2}^{{h}_{2}}\right)+{A}_{1}\cdot {r}_{-1}+{A}_{2}\cdot {r}_{-2}\\ \frac{{{{\rm{d}}}}{A}_{1}}{{{{\rm{d}}}}t}	=-{A}_{1}\cdot \left({r}_{-1}+{r}_{2}^{{\gamma }_{12}}{({\alpha }_{12}{d}_{2})}^{{\gamma }_{12}{h}_{2}}\right)+U\cdot {r}_{1}{d}_{1}^{{h}_{1}}+{A}_{1,2}\cdot {({r}_{-2})}^{{\gamma }_{12}}\\ \frac{{{{\rm{d}}}}{A}_{2}}{{{{\rm{d}}}}t}	=-{A}_{2}\cdot \left({r}_{1}^{{\gamma }_{21}}{({\alpha }_{21}{d}_{1})}^{{\gamma }_{21}{h}_{1}}+{r}_{-2}\right)+U\cdot {r}_{2}{d}_{2}^{{h}_{2}}+{A}_{1,2}\cdot {({r}_{-1})}^{{\gamma }_{21}}\\ \frac{{{{\rm{d}}}}{A}_{1,2}}{{{{\rm{d}}}}t}	=-{A}_{1,2}\cdot \left({r}_{-1}^{{\gamma }_{21}}+{r}_{-2}^{{\gamma }_{12}}\right)+{A}_{1}\cdot {r}_{2}^{{\gamma }_{12}}{({\alpha }_{12}{d}_{2})}^{{\gamma }_{12}{h}_{2}}+{A}_{2}\cdot {r}_{1}^{{\gamma }_{21}}{({\alpha }_{21}{d}_{1})}^{{\gamma }_{21}{h}_{1}}$$

At equilibrium, the Eq. (12) must all be equal to zero; however, the system only defines a rank 3 matrix. Taking the first three equations from (12) with the constraint *U* + *A*_1_ + *A*_2_ + *A*_1,2_ = 1, we define

13 $${{{\bf{M}}}}:= \left[\begin{array}{llll}-\left({r}_{1}{d}_{1}^{{h}_{1}}+{r}_{2}{d}_{2}^{{h}_{2}}\right)&{r}_{-1}&{r}_{-2}&0\\ {r}_{1}{d}_{1}^{{h}_{1}}&-\left({r}_{-1}+{r}_{2}^{{\gamma }_{12}}{({\alpha }_{12}{d}_{2})}^{{\gamma }_{12}{h}_{2}}\right)&0&{({r}_{-2})}^{{\gamma }_{12}}\\ {r}_{2}{d}_{2}^{{h}_{2}}&0&-\left({r}_{1}^{{\gamma }_{21}}{({\alpha }_{21}{d}_{1})}^{{\gamma }_{21}{h}_{1}}+{r}_{-2}\right)&{({r}_{-1})}^{{\gamma }_{21}}\\ 1&1&1&1\end{array}\right]$$

such that

$${{{\bf{M}}}}\cdot {\left[U\quad {A}_{1}\quad {A}_{2}\quad {A}_{1,2}\right]}^{T}={\left[0\quad 0\quad 0\quad 1\right]}^{T}$$

or, solving for the proportions of each state,

14 $${\left[U\quad {A}_{1}\quad {A}_{2}\quad {A}_{1,2}\right]}^{T}={{{{\bf{M}}}}}^{-1}\cdot {\left[0\quad 0\quad 0\quad 1\right]}^{T}$$

If we again consider distinct effects *E*_0_, *E*_1_, *E*_2_, and *E*_3_ distinguishing populations *U*, *A*_1_, *A*_2_, and *A*_1,2_, we find the equation for the dose-response surface to be

15 $$E=\left[{E}_{0}\quad {E}_{1}\quad {E}_{2}\quad {E}_{3}\right]\cdot {{{{\bf{M}}}}}^{-1}\cdot {\left[0\quad 0\quad 0\quad 1\right]}^{T}$$

As *d*1 → *∞* the equation reduces to

16 $$E={E}_{3}+\frac{{E}_{1}-{E}_{3}}{1+{\left(\frac{{\alpha }_{12}d2}{C2}\right)}^{{\gamma }_{12}h2}}$$

by which we can see the 2D equation reduces to a 1D Hill equation at the boundaries (See Supplemental Section *Proof of boundary behavior of 2D Hill equation*).

### Re-examining the sham experiment: Sham compliance introduces Hill-dependent bias in DEP models

A new synergy model’s consistency is traditionally tested with the “sham” combination thought experiment. In a sham experiment, a single drug is considered as though it were a combination, with the expectation that the drug should be neither synergistic nor antagonistic with itself. DEP frameworks, characterized by linear isoboles, are known to satisfy the sham experiment, while MSP frameworks famously do not^9^.

In Box 2, we show MuSyC only satisfies the sham experiment when *h* = 1, which makes sense as MuSyC produces linear isoboles only in this condition (Fig. S2B and Eq. 25). Further, our analysis in Box 2 revealed that sham combinations exhibit unique biochemistry, only equivalent to true combinations in the case *h* = 1. When *h* ≠ 1, combinations contain intermediate states representing mixed-inhibition (black circles, Fig. 5A, B). In sham combinations, these mixed-inhibition states are equivalent to single drug complete-inhibition states (Fig. 5A, cyan circles), while for real combinations, these are *not* equivalent (Fig. 5B). When *h* = 1, these intermediate mixed-inhibition states do not exist, explaining the concordance between sham and true combinations in this case. We expect when *h* ≠ 1, enforcing sham compliance leads to predictable systematic biases. We expect when *h* < 1 DEP frameworks will overestimate synergy (Fig. S2C), and when *h* > 1, DEP frameworks will overestimate antagonism (Fig. S2D).

In combinations from the anti-cancer dataset, the average trend of Loewe synergy closely follows the Hill slope bias predicted by MuSyC (Fig. 5C). Further, subtracting the MuSyC-predicted bias from Loewe values for each combination results in a distribution independent of Hill slope (bottom panel). The bias toward synergy is particularly large for drugs with low Hill slopes. As an example, both doxorubicin (DNA damaging agent) and mk-4827 (PARP inhibitor) have small Hill slopes when applied to MBA-MB-436 cells, and their combination is synergistic by Loewe. However, using MuSyC, we see this combination is both antagonistically efficacious and antagonistically potent (Fig. 5D). In one dataset (Cokol et al.), this MuSyC-predicted bias revealed a screen-wide underestimation of synergy by Loewe (Fig. S6A,C).

Therefore, satisfying sham compliance biases models toward synergy for drugs with low Hill slopes, regardless of with what these drugs are combined. This bias—which stems from enforcing a biochemical reaction scheme only appropriate for sham combinations—should be sufficient grounds for dismissing the sham experiment as a measure of a new synergy framework’s validity.

Box 2: Sham compliance of MuSyC, and the mass action biochemistry of a sham experiment To simulate a sham experiment using MuSyC, there is no state *A*_1,2_ (Fig. 1B), which requires *α*_12_ = *α*_21_ = 0. Further, because drugs 1 and 2 are the same, *h*_1_ = *h*_2_ = *h*, *C*_1_ = *C*_2_ = *C*, and *E*_1_ = *E*_2_. Thus, the 2D Hill equation (Eq. (15)) reduces to

17 $${E}_{d}({d}_{1},{d}_{2})=\frac{{E}_{0}+{E}_{1}\frac{{d}_{1}^{h}+{d}_{2}^{h}}{{C}^{h}}}{1+\frac{{d}_{1}^{h}+{d}_{2}^{h}}{{C}^{h}}}\quad \,{{{\mbox{MuSyC}}}}\; {{{\mbox{Sham}}}}\,$$

In comparison, the true dose-response surface of a sham experiment can be analytically determined from the 1D Hill dose-response equation (Eq. (10)) as

18 $${E}_{d}({d}_{1},{d}_{2})	={E}_{{{{\mathrm{single}}}}}({d}_{1}+{d}_{2})\\ 	=\frac{{E}_{0}+{E}_{1}{\left(\frac{{d}_{1}+{d}_{2}}{C}\right)}^{h}}{1+{\left(\frac{{d}_{1}+{d}_{2}}{C}\right)}^{h}}\quad \,{{{\mbox{True}}}}\; {{{\mbox{Sham}}}}\,$$

Equations (17) and (18) are only equivalent when *h* = 1. This makes sense, as the constraints on *α* and *h* are the conditions required for MuSyC to satisfy the DEP (Fig. 2B). To see what happens when *h* ≠ 1, consider, for instance, a molecule with three binding sites targeted by a small molecule inhibitor (*h* = 3) (Fig. 5A, B). For clarity, we assert *E*_0_ = 1 and *E*_1_ = 0, though the findings are valid more generally. The MuSyC sham surface follows

$${E}_{d}({d}_{1},{d}_{2})={\left(1+\frac{{d}_{1}^{3}+{d}_{2}^{3}}{{C}^{3}}\right)}^{-1}\quad \,{{{\mbox{MuSyC}}}}\; {{{\mbox{Sham}}}}\,$$

In contrast, the true sham surface is

19 $${E}_{d}({d}_{1},{d}_{2})	={\left(1+{\left(\frac{{d}_{1}+{d}_{2}}{C}\right)}^{3}\right)}^{-1}\\ 	={\left(1+\frac{{d}_{1}^{3}}{{C}^{3}}+3\frac{{d}_{1}^{2}{d}_{2}}{{C}^{3}}+3\frac{{d}_{1}{d}_{2}^{2}}{{C}^{3}}+\frac{{d}_{2}^{3}}{{C}^{3}}\right)}^{-1}\quad \,{{{\mbox{True}}}}\; {{{\mbox{Sham}}}}\,$$.

The two additional cross-terms in the true sham equation ($3\frac{{d}_{1}^{2}{d}_{2}}{{C}^{3}}$ and $3\frac{{d}_{1}{d}_{2}^{2}}{{C}^{3}}$) describe the six possible mixtures of drugs 1 and 2 that, together, fill all binding sites (Fig. 5A, blue, green, and magenta paths show three possible mixtures). In a sham experiment, because drugs 1 and 2 are the same, the diagonal states (black and cyan circles) in Fig. 5A are all equivalent, and fully inhibited.

Conversely, in non-sham combinations, drugs rarely target the same binding sites, or even the same molecule. Even when two drugs are mutually exclusive inhibitors of the same molecule and have the same number of binding sites, the cross-terms describe non-equivalent, not fully inhibited states (Fig. 5B). A commonly applied and physiologically supported approximation is that only fully bound molecules become (in)active (see reaction schemes 5–7 in Weiss^54^). Partially bound cross-terms are therefore reasonably modeled as unaffected, and the absence of these cross-terms from Eq. (17) is justified for real (non-sham) combinations (see Discussion section). Further, when the two drugs do not target the same molecule or are mutually exclusive or have the same number of binding sites, by far the preponderance of real combinations, the diagonal states are ill defined yet remain embedded in the sham equation.

### MuSyC reveals errors in the derivation and application of the Combination Index

**Fig. 6:**
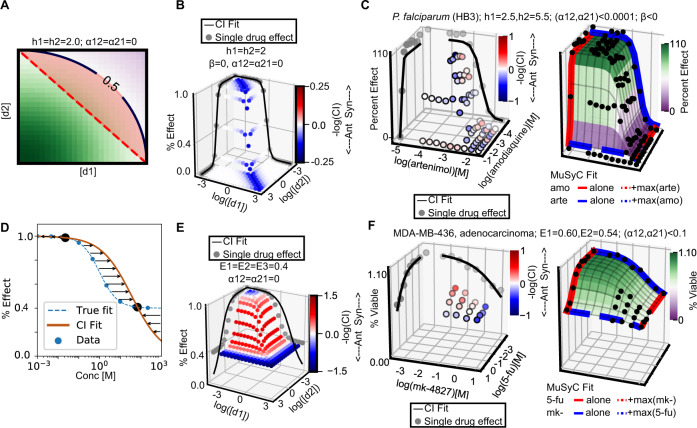
Comparing MuSyC and CI. **A** Linear isoboles, characteristic of CI, emerge from MuSyC when the drugs are mutually exclusive (*α*_12_ = *α*_21_ = 0) and *h*_1_ = *h*_2_ = 1 (Fig. S2). When *h* ≠ 1, MuSyC predicts non-linear isoboles. Here, CI would erroneously assess dose pairs in the red shaded region (between straight, dotted, diagonal line, and curved line above it) as antagonistic. **B** An example, synthetic two-drug dataset where this Hill-dependent bias is apparent (*h*_1_ = *h*_2_ = 2, *E*_0_ = 1, *E*_1_ = *E*_2_ = 0.0, *C*_1_ = *C*_2_ = 1, *α*_12_ = *α*_21_ = 0). This trend is most apparent near *d*_1_ ≈ *d*_2_. **C** The combination of artenimol (alkylating agent) and amodiaquinine (putative inhibitor of heme polymerase activity) in the HB3 strain of malaria (chloroquine sensitive)^25^. Left panel shows the predicted CI bias (antagonism along diagonal due to *h* > 1). Right panel shows MuSyC’s fit. Because *E*_0_ = 1, *E*_1_ = *E*_2_ = 0, and (*α*_12_, *α*_21_) < 0.0001, the difference between MuSyC and CI, is due to the Hill slope error in CI. **D** A synthetically generated dose-response dataset (blue points) with (*E*_0_ = 1, *E*_1_ = 0.4, *C* = 1, *h* = 1) is fit using the CI method (*E* = *f*(*d*), orange line), which assumes the effect must range from 100% to 0%. To calculate synergy, CI uses *f*^−1^(*E*) (orange line). Residuals in the CI fit of *f*^−1^(*E*) to the true data are annotated with arrows. Black dots denote where the CI fit and true curve (blue dotted line) cross. CI overestimates *f*^−1^(*E*) for effects between the black dots, and underestimates it for effects outside the black dots. When CI overestimates *f*^−1^(*E*), we expect it to overestimate synergy. Likewise, when CI underestimates *f*^−1^(*E*) we expect it to underestimate synergy. **E** A synthetic two-drug dataset with partially efficacious drugs (*E*_0_ = 1, *E*_1_ = *E*_2_ = 0.4, *C*_1_ = *C*_2_ = 1, *h*_1_ = *h*_2_ = 1, *α*_12_ = *α*_21_ = 0) is analyzed with CI. As expected, in regions where CI overestimates *f*^−1^(*E*), it overestimates synergy (red), and likewise for underestimation (blue). **F** Experimental combination of drugs with intermediate efficacy showing (left) CI’s effect-dependent bias and (right) MuSyC’s fit. The combination is mk-4827 (PARP inhibitor) combined with 5-fu (thymidylate synthase inhibitor) in MDA-MB-436 breast adenocarcinoma^26^ (BRCA1 mutant^51^). Because MuSyC finds (*α*_12_, *α*_21_) < 0.1 and *h*_1_ ≈ *h*_2_ ≈ 1, the principle difference between the MuSyC model and CI relates to the effect range. As predicted, CI is antagonistic at the highest doses, despite these being the only doses which achieve greater effect than either drug alone (*β* = 0.17).

Recent reports have identified potential flaws with the use of CI^11,36^, yet it remains the most highly cited synergy metric^30,34,37^. CI has recently been proposed to the Food and Drug Administration (FDA) and National Institutes of Health (NIH) as the de facto definition of drug synergy^31^. Due of its prominence in the field, here we specifically examine its behavior with respect to the biases discussed above. We find CI and MuSyC have the same null model when *h*_1_ = *h*_2_ = 1, *α*_12_ = *α*_21_ = 0, *E*_0_ = 1, *E*_1_ = *E*_2_ = 0 (Supplemental Section Relationship between different synergy frameworks). The presence of constraints on *h* and *E* indicates CI could be impacted by both a Hill-slope and efficacy range bias, like those we reported above for DEP and MSP-based frameworks, respectively. Like MuSyC, CI is based on the Law of Mass-Action, facilitating a direct comparison of their formulations. In doing so, we found two errors in CI with significant consequences: (1) a fundamental math error in its derivation impacting combinations with *h* ≠ 1 (Box 3, Fig. 6A–C), and (2) a fitting error that arises when applied to drugs with partial efficacy (*E*_1_ or *E*_2_ > 0) (Fig. 6D–F).

Details of the error in the derivation of CI are in Box 3. Because of this error, the CI equation is only valid for combinations of drugs with Hill slopes equal to one (*h*_1_ = *h*_2_ = 1). In this regime, MuSyC also results in linear isoboles (Fig. S2B). When *h* ≠ 1 (Figs. 6A and S8A), the CI equation incorrectly factors the exponent outside the sum, introducing the exact same cross terms that we show (Box 2) are only valid for sham combinations, and not valid for all combinations when *h*_1_ ≠ *h*_2_ ≠ 1. Therefore, when *h* ≠ 1, CI suffers from the same Hill-slope dependent bias we show in Fig. 5. We show the consequence of this bias in illustrative two-drug examples using synthetic (Figs. 6B and S8B) and experimental combinations (Figs. 6C and S8C).

Even when *h* = 1, CI requires that the maximal and minimal effects (*E*_0_, *E*_1_, Fig. 1) be fixed at 1.0 and 0. Subsequently, CI fits the single-drug dose-response curves using the two-parameter median-effect equation^30^ (Eq. (8)), see also Supplemental Section Percent Affect vs Percent Effect—Combination Index). In contrast, MuSyC is based on a four-parameter Hill equation (Box 1, Eq. (10)), and thus can describe metrics of drug effects with arbitrary ranges. It is common to observe in many dose-response assays, such as percent viability, drug effects that do not reach 0% (i.e. *E*_1_ > 0)^38^. For such assays, the two-parameter median-effect equation fits poorly (Fig. 6D). These poor fits lead to an effect-dependent error in CI quantification of synergy as observed in synthetic (Fig. 6E) and experimental combinations (Fig. 6F). We note, this effect-dependent error is not the same as the effect-range bias we report for MSP-based frameworks in Section “MSP is biased against combinations of drugs with intermediate efficacy”.

Box 3: Derivation error in combination index Both MuSyC and combination index (CI) describe combination drug responses using the Law of Mass Action. The common form of CI was developed from a model of mutually exclusive inhibitors, and MuSyC reproduces this model when *α*_12_ = *α*_21_ = 0 (Fig. 1B). Surprisingly though, even in this case, MuSyC and CI disagree. CI predicts additive drug combinations will result in linear isoboles, irrespective of the Hill slope, while MuSyC (with *α*_12_ = *α*_21_ = 0) predicts linear isoboles only when *h*_1_ = *h*_2_ = 1 (Fig. 6A and Eq. 25). This motivated us to compare the derivations of CI and MuSyC to understand this discrepancy.

While formalized in its current form in Chou and Talalay^34^, the mathematics underlying CI are developed in Chou and Talalay^55^. We found an error in the section *Inhibition of the Higher-Order Kinetic Systems by Mutually Exclusive Inhibitors*, which is responsible for the discrepancy between MuSyC and CI. Specifically, when the authors consider the special-case of *h* = 1, they correctly solve for the ratio of inhibited (affected) to uninhibited (unaffected) targets after treatment with *n*-drugs as

$$\frac{A}{U}=\mathop{\sum }\limits_{j=1}^{n}\frac{{d}_{j}}{{C}_{j}}\quad {{{\rm{Eq.}}}}\,11\,{{{{\rm{from}}}}}\,{{{\mathrm{Chou}}}}\,{{{\mathrm{and}}}}\,{{{\mathrm{Talalay}}}}^{55}$$

as well as the the 1-drug case with arbitrary *h* (the median-effect equation (Eq. (8)).

$$\frac{\overline{{A}_{j}}}{\overline{{U}_{j}}}={\left(\frac{{d}_{j}}{{C}_{j}}\right)}^{{h}_{j}}\quad {{{\rm{Eq.}}}}\,12\,{{{{\rm{from}}}}}\,{{{\mathrm{Chou}}}}\,{{{\mathrm{and}}}}\,{{{\mathrm{Talalay}}}}^{55}.$$

See Table S1 for details of how we translated variable names from Chou and Talalay^55^. However, the authors incorrectly generalized these two equations to an *n*-drug, arbitrary *h* case by erroneously “relating the first-order Eq. (11) to the [h]th order relationship ... Eq. (12)” (Chou and Talalay^55^, pg. 209)

20 $${{{\rm{INVALID}}}}\quad \frac{A}{U}={\left(\mathop{\sum }\limits_{j = 1}^{n}\frac{{d}_{j}}{{C}_{j}}\right)}^{h}\quad {{{\mbox{Unlabeled}}}}\; {{{\mbox{equation}}}}\; {{{\mbox{from}}}}\; {{{\mathrm{Chou}}}}\; {{{\mathrm{and}}}}\; {{{\mathrm{Talalay}}}}^{55},\,{{{\rm{pg}}}}.\,209$$

To show this is incorrect, consider *n* mutually exclusive drugs following Hill kinetics (a model graphically represented for *n* = 2 in Fig. 2B). At equilibrium (see Box 1), for each drug *j* we assert

$$\frac{{{{\rm{d}}}}{A}_{j}}{{{{\rm{d}}}}t}=U{r}_{j}{d}_{j}^{{h}_{j}}-{A}_{j}{r}_{-j}\equiv 0$$

where ${C}_{j}\equiv {\left(\frac{{r}_{-j}}{{r}_{j}}\right)}^{\frac{1}{{h}_{j}}}$. From this we can solve $\frac{{A}_{j}}{U}={\left(\frac{{d}_{j}}{{C}_{j}}\right)}^{{h}_{j}}$ for each drug. Because $A=\mathop{\sum }\nolimits_{j = 1}^{n}{A}_{j}$, we instead find the correct form for the multi-drug case with arbitrary *h* is

21 $${{{\rm{CORRECT}}}}\quad \frac{A}{U}=\mathop{\sum }\limits_{j=1}^{n}{\left(\frac{{d}_{j}}{{C}_{j}}\right)}^{{h}_{j}}$$

The error in the invalid equation factors the exponent *h* outside the sum, whereas we see each term in the sum must be raised to its own *h*_*j*_. When *h*_*j*_ = *h* = 1 the discrepancy between these equations vanishes. However the authors use the incorrect version to define the CI^34^, irrespective of *h*. This accounts for the discrepancy between CI and MuSyC, and introduces a Hill-slope dependent bias in CI calculation of synergy (Figs. 6 and S8). Specifically, CI underestimates synergy when *h* > 1 (Fig. 6D–F), and overestimate synergy when *h* < 1 (Fig. S8). The ubiquity of this error in DEP frameworks applied to real data is further addressed in Section *Re-examining the sham experiment: Sham compliance introduces Hill-dependent bias in DEP models*.

## Discussion

Herein, we have demonstrated four key advances of MuSyC^21^ germane to the study of combination pharmacology: (1) the unification of the DEP and MSP; (2) the decoupling of three distinct types of synergy; (3) the revelation of biases emerging from constraints on the single drug pharmacological profile inherent in the DEP and MSP; and (4) reporting on flaws of highly cited CI including a mathematical, derivational error which impacts its reliability for synergy quantification.

The DEP and MSP have formed the foundational principles of most synergy frameworks over the last century; however, the connection between these principles has remained unknown^4,5^. Here, approaching combination pharmacology using the Law of Mass Action applied to a state-transition model results in a single framework unifying both principles. By mapping all frameworks on a common landscape, MuSyC facilitates rigorous investigation of oft-cited, contradictory conclusions between existing frameworks^16^—contradictions that preclude reproducibility between synergy studies. Specifically, as is seen in Fig. 2C, there is no combination which can simultaneously satisfy the conditions required by both DEP and MSP synergy frameworks. Previous works advocate prioritizing combinations that are synergistic by all methods^5^, or choosing a synergy model carefully based on the data measured and shape of dose-response curves^32^. In contrast, MuSyC’s unification of DEP and MSP means it is applicable regardless of whether the data are best described by the DEP or MSP, or something in-between, so no choice is required. Further, while MuSyC’s mass-action formalism applies most directly to molecular inhibition, its synergy parameters describe geometric transformations of efficacy, potency, and Hill slope, which are well-established quantities used to describe sigmoidal responses, regardless of mechanism.

One key advance of MuSyC, facilitating this unification, was the decoupling of *α*, *β*, and *γ*. These synergy parameters correspond directly to classic, pharmacological measures of a drug’s potency, efficacy, and cooperativity. By calculating synergy in this way, interpretation of synergy does not depend on arbitrary expectations or thresholds. Rather, an *α* of 10 corresponds to a 10-fold increase in a compound’s potency, as a result of the other drug, regardless of whether we define *α* = 1 or *α* = 10 as the “threshold” for synergy. As practical advice for accurately fitting all synergy parameters, we recommend sampling (*d*_1_, *d*_2_) around the four corners (0, 0), (*d*_1,max_, 0), (0, *d*_2,max_), and (*d*_1,max_, *d*_2,max_) to best constrain synergistic efficacy, and around the four edges (*C*_1_, 0), (*C*_1_, *d*_2,max_), (0, *C*2), (*d*_1,max_, *C*2) to best constrain synergistic potencies and cooperativities, where *d*_*i*,max_ is an asymptotically high dose of drug *i*. We refer interested readers to Supplemental Section “Interactive MuSyC Jupter Notebook”, Figs. S12–S16, and Supplemental Code 1, which provide an interactive demonstration that shows how each synergy parameter results in different outputs across multiple concentrations. We envision distinguishing synergies of potency, efficacy, and cooperativity will be of differential consequence in alternate contexts. For example, in cancer synergistic efficacy may be most important, while for neurological disorders, synergistic cooperativity—i.e. sharp on-off drug response —may be preferred. In an analysis of clinical trials of combination therapies, we find synergistic efficacy is statistically higher in clinically efficacious combinations than clinically non-efficacious combinations (see Supplemental Section MuSyC statistically distinguishes efficacious and non-efficacious drug combinations in clinical trials based on combination screens, Figs. S9 and S10).

The relationship between MuSyC and the MSP and DEP frameworks (Fig. 2) is constrained by monotherapy parameters (*E*_1_, *E*_2_ for MSP, *h* for DEP). These constraints suggested systematic biases in MSP and DEP frameworks contingent on a single drug’s efficacy (MSP, Fig. 4) and Hill slope (DEP, Fig. 5). These systematic biases merit consideration when using these frameworks for drug discovery in large screens, or when accounting for batch-effects across different datasets (Fig. S6). Such systematic biases can confound machine learning models to predict synergy, decreasing their utility. Additionally, the constraint on *h* highlighted a discrepancy between the biochemistry of true sham experiments and real combinations. The centrality of the sham experiment in the drug synergy literature cannot be overstated; however, we argue enforcing sham compliance comes at the cost of improperly modeling real combinations, leading to a predictable Hill-dependent bias.

CI has previously been criticized for its procedure to fit the median-effect equation. Specifically, CI depends on a log-transformation in order to linearize the two-parameter median effect equation “in similar logic to the defunct Scatchard analysis in pharmacology, which has been replaced by non-linear modeling”^36^. That is, this log-transformation alters the noise profile such that small deviations for effects at low concentrations result in large deviations in the synergy calculations. Additionally, this transformation results in information loss, since undefined values for effects outside the range of (0,1) are forcibly removed, and as a consequence, CI synergy estimates become “statistically unstable” for noisy experimental data^11^. Beyond these valid points regarding CI’s practical application, here we uncover a mathematical error in the derivation of CI (Box 3) causing a systematic bias depending on the Hill slope. Because these flaws compound in non-linear ways, the expected error when applying CI is unique to each particular combination and assay design.

The prospects of higher-order synergies (i.e., interactions beyond pairwise) and scaling laws for drug mixtures, while provocative, have remained contentious^6,12,39,40^. MuSyC’s cubic geometry allows it to be easily extended to three or more drugs (Fig. S1), and we expect MuSyC will enable a more refined search for higher-order interactions. For instance, combinations that mix different synergy profiles (e.g., drugs 1 and 2 are synergistically potent, and drugs 2 and 3 are synergistically efficacious) may exhibit different higher-order interactions than combinations all sharing a single synergy type. However, the number of synergy parameters in MuSyC scales as 2^*N*^(*N* + 1) − 3*N* − 1 (including *γ*) where *N* is the number of drugs (Fig. S1), and the commensurate data necessary to fully constrain MuSyC hyper-surfaces invokes a parameter identifiability problem (“the curse of dimensionality”). Nonetheless, MuSyC’s geometry could be leveraged to guide sampling schemes to constrain the boundaries, allowing the solution to be built up step-wise from the boundaries (see Supplemental Section Proof of boundary behavior of the 2D Hill equation).

MuSyC expects single-drug dose-response curves to be sigmoids well fit by the 1D Hill equation (Eq. (10)), and dose-response surfaces to be well fit by the 2D Hill equation (Eq. (15)). In our experience, these expectations are met by real data, as most single drugs have monotonic, sigmoidal responses, and even complex drug interactions can be modeled using various mixtures of *α*, *β*, and *γ* (96% and 88% of combinations in anti-cancer and anti-malarial datasets had *R*^2^ > 0.7, respectively). However, it is possible for drugs to have multiphasic responses due to poly-pharmacology which are not well fit by a Hill curve. It may be possible to extend MuSyC to encompass such drugs—for instance by including a multiphasic Hill model^41^ or modeling effects of “partially affected” states (Fig. 5A, B and Box 3). In extreme cases, it may only be possible to apply non-parametric frameworks such as Bliss, Loewe, or HSA. Nevertheless we note that without fitting dose-response curves to a parametric model, these metrics are sensitive to noise in individual data-points. Replicate measurements may be able to reduce this sensitivity. Additionally, MuSyC assumes all drugs are administered concurrently, whereas patient treatments are often staggered. New theory and experimental methods are needed to address the synergy of combinations which are staggered temporally, bridging the synergy of pharmacodynamics with the synergy of pharmacokinetics. Finally, in the datasets we analyzed here, we did not find a role for synergistic cooperativity (*γ*). Future studies in other systems are needed to better understand situations when synergistic cooperativity is expected.

By viewing the landscape of drug synergy through the lens of mass-action, we have demonstrated the underlying assumptions, limitations, and biases of commonly applied synergy methods. We have shown how MuSyC unifies the DEP and MSP thus providing a consensus framework for the study of combination pharmacology. These findings provide much needed clarity to the field and should promote the reproducibility of drug synergy studies across drug combination discovery efforts. Such a rigorous approach to the discovery and application of drug combinations will open the door to the discovery of new and previously discarded avenues for therapeutic mixtures.

## Methods

We note the synergy calculations conducted for the different published datasets were not necessarily the same as those used in the original paper. Indeed the limitations of the current frameworks forced customized analysis for each publication highlighting the need for a consensus framework. However, in order to compare between datasets, we have calculated Bliss, Loewe, HSA, and other synergy frameworks, as described below, from the raw data.

### Software

#### Implementation and website

A web application to calculate MuSyC synergy parameters for users’ data is available at https://musyc.lolab.xyz/. Experimental data are uploaded in comma-separated value (CSV) format; data format details and usage instructions are in the supplemental materials. The application fits dose-response surfaces using MuSyC and offers the results both as a CSV download of fit parameters, and interactive plots of the dose-response surface.

The web application uses the Django web application framework (djangoproject.com) and Python 3.7. Fitting tasks are processed asynchronously using a message queue (RabbitMQ; rabbitmq.com) and task-worker framework (Celery; celeryproject.org). Data are organized in a Postgres relational database (postgres.org). The following packages were used for fitting, data analysis, or visualization: SciPy v1.1.0^42^, Numpy v1.14.3^43^, Pandas v0.23.0^44^, Matplotlib v2.2.2^45^, uncertainties v3.0.2^46^.

#### Fitting 2D Hill equation

Here we describe fitting protocol for drug metrics where the metric of drug effect decreases as dose increases (*E*_0_ > *E*_3_) (e.g., anti-proliferative drugs); however, the framework is equally valid if increasing the drug corresponds to increases the effect (*E*_0_ < *E*_3_) (e.g., percent effect).

Previously, we found it necessary to use a Metropolis Hastings Monte Carlo (MCMC) seeded with a particle swarm optimization (PSO) to fit the 2D Hill equation^21^. This was prompted by the inconsistent performance of standard non-linear least squares (NLLS) regression. In particular, we observed instances of calculated uncertainties in NLLS which were several orders of magnitude greater than the parameter value. This, we have discovered, is because the multi-collinearity between the Hill slope and the EC50 (*C*) inherent in the structure of the Hill equation—collinearities which are amplified when extending the Hill equation to 2D. The correlation between variable *h* and *C* is easiest to observe in a linearized 1D Hill equation (Eq. (1)).

1 $${{{\mathrm{log}}}}\,\left(\frac{{E}_{0}-{E}_{m}}{E-{E}_{m}}-1\right)=h* {{{\mathrm{log}}}}\,(d)-h* {{{\mathrm{log}}}}\,(C)$$

In Eq. (1), the intercept of the line ($h* {{{\mathrm{log}}}}\,(C)$) depends on the slope of the line (*h*). This correlation is problematic when trying to estimate the parameter uncertainty (*σ*) from NLLS regression because *σ* is estimated as the square root of the inverse Hessian, approximated to be **J**^*T*^**J** (where **J** is the Jacobian at the solution). **J** of the 4-parameter Hill equation is

2 $${{{\bf{J}}}}=\left[\frac{\partial }{\partial {E}_{0}}\quad \frac{\partial }{\partial {E}_{m}}\quad \frac{\partial }{\partial h}\quad\frac{\partial }{\partial C}\right]$$

3 $$=\left[\begin{array}{llll}1-\frac{1}{{\frac{d}{C}}^{h}+1}&\frac{1}{{\frac{d}{C}}^{h}+1}&-\frac{({E}_{0}-{E}_{m}){\left(\frac{d}{C}\right)}^{h}{{{\mathrm{log}}}}\,\left(\frac{d}{C}\right)}{{\left({\left(\frac{d}{C}\right)}^{h}+1\right)}^{2}}&-\frac{h({E}_{m}-{E}_{0}){\left(\frac{d}{C}\right)}^{h}}{C{\left({\left(\frac{d}{C}\right)}^{h}+1\right)}^{2}}\end{array}\right]$$

When the Hill slope is large (e.g., *h* > 5), the second two terms of the **J** cause the numerical approximation of the inverse of *J* to be undefined. This problem is compounded in the 2D Hill equation where, in addition to *h* and *C*, the parameters *α* and *γ* are co-linear. However, this does not affect the accuracy of the fitted parameter values from the NLLS regression—only the parameter uncertainty^47^.

For the fitting the 2D Hill equation in this study, we adopted a Monte Carlo sampling approach to calculate the fit parameter uncertainty. This is significantly faster than our previous method (PSO + MCMC)^21^ while maintaining reasonable calculations of the parameter uncertainties accounting for the multi-collinearities described above. The Monte Carlo algorithm for fitting the 2D Hill equation is as follows. First, the 4-parameter 1D Hill equation (Eq. (10)) is fit to the dose-response of each drug in isolation. The fit uses the Trust Region Reflective (TRF) algorithm implemented in the *curve*_*fit*() module of the scipy.optimization package. *h* and *C* were unconstrained while *E*_max_ and *E*_0_ are constrained for each dataset as annotated in the Methods section, data acquisition, preparation, and analysis. In general, adjusting the parameter bounds to closely match what is feasible for the given dataset will lead to better parameter estimates, helping the curve fitting algorithm to avoid becoming stuck in a suboptimal local minima. The initial 1D Hill fits provide estimates for (*E*_0_, *E*_1_, *E*_2_, *C*_1_, *C*_2_, *h*1, *h*2), because the 2D Hill equation becomes equivalent to the 1D Hill equation in the limit as *d*_*i*_ → 0. In practice, best fits of these parameters in the 2D Hill equation which have counterparts from the 1D Hill equation tend to be similar to their monotherapy fits which were used as initial guesses. However we note that it is possible for these values to differ significantly from their monotherapy best guesses when the monotherapy data are noisy, and thus can have wide uncertainties. Next the 2D Hill equation (Eq. (15)) is fit using the TRF algorithm with initial values based on the 1D Hill equation fits and with bounds based on the parameter uncertainty calculated for the 1D Hill fits. The initial values for parameters unique to the 2D Hill equation, *E*_3_, *α*_21_, *α*_12_, *γ*_12_, *γ*_21_ are ($\min ({E}_{1},{E}_{2})$,1,1,1,1). For all combinations *r*_1_ = *r*_2_ = 100. The bounds for ${{{\mathrm{log}}}}\,({\alpha }_{21}),{{{\mathrm{log}}}}\,({\alpha }_{12})$ are set to [−4,4]. From this initial fit, 100 Monte Carlo samples are used to calculate the parameter uncertainty as described by Motulsky and Christopoulos^47^, (Chapter 17: Generating confidence intervals by Monte Carlo, pg. 104). Specifically, noise, with a distribution N(0,*σ*), where *σ* is equal to the root mean square (RMS) of the best fit, is added to best-fit values of the 2D Hill equation for all drug doses. The data plus noise is then fit again initializing the optimization from the best-fit parameters of the original data. This is done 100 times. From this ensemble of fits, the 95% confidence interval of each parameter can be calculated. This Monte Carlo approach results in asymmetric confidence intervals which better captures the non-Gaussian distribution of uncertainty for many fits (e.g. the distribution of *h* is log-normal) as well as being robust to the co-linear parameters in the 2D Hill equation. The asymmetric confidence interval is particularly salient when the dose-range is insufficient to observe the lower plateau of the dose-response. Only combinations for which *R*^2^ > 0.7 and the fitted EC50s of both drugs was less than maximum tested dose for each (${C}_{1} < \max (d1),{C}_{2} < \max (d2)$) were included for subsequent analysis.

#### Comparing fitting algorithm robustness between different synergy frameworks

We additionally examined the performance of the MuSyC fitting when the raw data is subject to different types of noise (Fig. S3A). We synthetically generated 10 random samples of 5400 noise and synergy profiles and compared between all parameterized models of synergy the percent of convergence (Fig. S3B), fit quality assessed by *R*^2^ (Fig. S3C), and variation in synergy parameters assessed by Z-score between the random samples (Fig. S3D). Fitting algorithms for the different models is described in Section Calculation of other synergy metrics. Overall, we find MuSyC performs as well as or better than comparable parameterized models of synergy on the tested synergy profiles. We note this analysis is hampered by the lack of a “true” standard for synergy necessitating dose-response surfaces to be generated based on a defined model—in this case the MuSyC model. Bliss, Loewe, and HSA in general do not require fitting an equation to the data, and were thus excluded from this analysis. Nevertheless we note such synergy metrics may be more sensitive to noise in the data, as noise in individual datapoints is not smoothed out via a curve fit.

### Data acquisition, preparation, and analysis

#### ONeil et al. anti-cancer screen

The anti-cancer drug combination data were downloaded from the supplemental materials of ONeil et al.^26^. Single agent and combination datasets were merged. Drug effect was the mean normalized percent viability (*X*/*X*_0_ column) calculated as detailed in ONeil et al.^26^. The minimum and maximum bounds for [*E*_0_, *E*_1_, *E*_2_, *E*_3_] during 2D Hill equation fits were [0.99,0.0,0.0,0.0,0.0] and [1.01,2.5,2.5,2.5], respectively.

#### Mott et al. anti-malarial screen

The anti-malarial drug combination data were downloaded from https://tripod.nih.gov/matrix-client/ from the Malaria Matrix project. Blocks downloaded for analysis were: [1601,1602,1603,1701,1702,1703,1761,1764]. Only blocks with a mqcConfidence of >0.9 were included. The drug effect was calculated as described in Mott et al.^25^. Effects <−20% and >120% were removed. The minimum and maximum bounds for [*E*_0_, *E*_1_, *E*_2_, *E*_3_] during 2D Hill equation fits were [90.,0.0,0.0,0.0] and [110,200,200,200], respectively.

#### Tan et al. anti-HIV screen

The anti-HIV drug combination data were downloaded from the supplemental table four of Tan et al.^28^. Drug effect was one minus the normalized infection rates as detailed in Tan et al.^28^. The minimum and maximum bounds for [*E*_0_, *E*_1_, *E*_2_, *E*_3_] during 2D Hill equation fits were [0.99,0.0,0.0,0.0,0.0] and [1.01,1.5,1.5,1.5], respectively.

#### Cokol et al. anti-fungal screen

The anti-fungal drug combination data were downloaded from supplemental dataset one in Cokol et al.^29^. The raw cell growth measurements for all 200 drug-drug interaction assays were stored as a 96 × 64 matrix of numbers. Rows were time points with 15 min intervals and columns are the indices of an 8 × 8 drug matrix. Drug dilutions were linear between the maximum reported in Table 1 of Cokol et al.^29^ and 0. The drug effect was quantified using the area under the growth curve (AUGC), calculated using Simpson’s integration, after the first 10 time points (150 min). The background unique to each experiment was removed by subtracting the minimum observed growth rate for each pair individually. The minimum and maximum bounds for [*E*_0_, *E*_1_, *E*_2_, *E*_3_] during 2D Hill equation fits were [0, 0, 0, 0] and [*∞*, *∞*, *∞*, *∞*], respectively.

#### Holbeck et al. anti-cancer screen

The ALMANAC anti-cancer drug combination data were downloaded from https://wiki.nci.nih.gov/display/NCIDTPdata/NCI-ALMANAC file *ComboDrugGrowth*_*Nov*2017. *zip*. The matching single dose-response data were downloaded from https://wiki.nci.nih.gov/display/NCIDTPdata/NCI-60+Growth+Inhibition+Data, June 2016 release *DOSE*_*RESPONSE* link. Single agent and combination datasets were merged using pandas dataframe operations. Drug effect was the percent growth inhibition calculated as detailed in^27^. The minimum and maximum bounds for [*E*_0_, *E*_1_, *E*_2_, *E*_3_] during 2D Hill equation fits were [99, −100, −100, −100, −100] and [101, 350, 350, 350], respectively.

### Calculation of other synergy metrics

#### Bliss, Loewe, and HSA

Bliss, Loewe, and HSA depend on the concentration of drugs so a combination can be synergistic at one dose, but antagonistic at another dose. Several methods have been proposed for extracting a single synergy metric per combination from these frameworks to enable comparisons between combinations^13–15,26^. For our analysis, we calculate the synergy score at the combination of each drug’s EC50 (Figs. 3 and 5) as proposed by Malyutina et al.^48^ or at the maximum tested concentration of each drug (Fig. 4). The EC50 of each drug was calculated from the fits to the 2D Hill Eq. (15) which we have observed to be more robust to noise when estimating the single drug pharmacologic profile. Assuming the notation for the 1D Hill equation and inverse Hill equation—which calculate effect (*E* given a dose (*d*) and a dose given an effect, respectively—are given by

$$\begin{array}{l}Hx(d)={E}_{x}+\frac{({E}_{0}-{E}_{x})}{1+{\left(\frac{{d}_{x}}{{C}_{x}}\right)}^{{h}_{x}}}\\ H{x}_{inv}(E)={C}_{x}* {\left(\frac{({E}_{0}-{E}_{x})}{(E-{E}_{x})}-1\right)}^{\frac{1}{{h}_{x}}}\end{array}$$

where *E*_*x*_ < *E*_0_, then equations for Bliss, Loewe, and HSA at the EC50 are:

4 $${{{\rm{Bliss}}}}=H1({C}_{1})* H2({C}_{2})-E({C}_{1},{C}_{2})$$

5 $${{{\rm{Loewe}}}}=\frac{C1}{H{1}_{inv}(Ed({C}_{1},{C}_{2}))}+\frac{{C}_{2}}{H{2}_{inv}(Ed({C}_{1},{C}_{2}))}$$

6 $${{{\rm{HSA}}}}=min\left(H1({C}_{1}),H2({C}_{2})\right)-E({C}_{1},{C}_{2})$$

where *Ed*(*C*_1_, *C*_2_) is the measured effect of combining *C*_1_ of *d*_1_ and *C*_2_ of *d*_2_. And equations for Bliss at the max of each drug is:

$$Bliss=H1(\max ({d}_{1}))* H2(\max ({d}_{2}))-E(\max ({d}_{1}),\max ({d}_{2}))$$

Thus, Loewe synergy is calculated using an equation similar to CI^34^, while Bliss and HSA are calculated using an “excess over” approach, which calculates the raw difference between the expected and observed responses. While the reference models are always the same, we note alternative equations may be used to quantify synergy of a combination^33,49^, though the biases we report are intrinsic to the reference models, not the synergy quantification approach. These equations assume the metric of drug effect decreases as the dose increases. Because many single agents did not reach 0% maximum efficacy, the EC50s (*C*_1_, *C*_2_) were not necessarily 50% (Fig. S7). If *E*(*C*_1_, *C*_2_) < *E*_1_, *E*_2_ then Loewe was undefined. We apply a $-{{{{\mathrm{log}}}}\,}_{10}$ transformation the scale Loewe to match the ranges Bliss and HSA are synergistic; therefore, f $-{{{{\mathrm{log}}}}\,}_{10}(Loewe)\, > \, 0$ the combination is synergistic and if $-{{{{\mathrm{log}}}}\,}_{10}(Loewe)\, < \, 0$ the combination is antagonistic. Additionally, for Figs. 3 and 5 we had to calculate the Hill-dependent bias in Loewe. For Fig. 3, we subtracted the Hill slope bias to only study the impact of conflating synergistic potency and efficacy. To calculate the bias, Loewe was calculated as in Eq. (5) where *Hx*_inv_ was was evaluated at the MuSyC-predicted *Ed*(*d*1, *d*2) for the combination retaining all the single drug parameters (*E*_0_, *E*_1_, *E*_2_, *C*_1_, *C*_2_, *h*1, *h*2) and assuming (*α*_12_ = *α*_21_ = 0). This resulted in an estimate of the bias purely due to the Hill slope in the Loewe calculation.

#### ZIP and BRAID

Both ZIP and BRAID were calculated for each combination using the R packages available for each method: (ZIP’s R code is in the supplemental file 1 of the manuscript^7^ and BRAID’s package is available from: https://cran.r-project.org/web/packages/braidReports/braidReports.pdf.

#### Effective dose model

To fit Zimmer et al.’s Effective Dose Model we used the scipy.optimization.curve_fit module in Python 2.7. Specifically, the Effective Dose Model, Eq. 2 in Zimmer et al.^50^ (Eq. 30 in Supplement), contains parameters (*C*_1_, *C*_2_, *a*_12_, *a*_21_, *h*_1_, *h*_2_) where the *a* parameters are the synergy values. In the model, there are no parameters for efficacy because it is assumed the drug effect ranges between zero and one. When this is not true, the Effective Dose Model results in poor fits to the data (Fig. S7A, B).

#### Schindler’s Hill PDE model

The Hill PDE model has no parameters to fit as the dose-response surface is derived the single dose-response curves. In fact, Schindler does not propose a method to estimate synergy from experimental data, but postulates some implementation of perturbation theory could be used to fit experimental data^8^. Therefore, to calculate the synergy of this model, we defined the sum of residuals between the null surface and the experimental data as the metric of synergy.

#### Combination Index

Following the CI fitting algorithm in the CompuSyn software, we fit the median-effect equation, a 2-parameter, log-linearized form of the Hill equation to each drug alone obtained by assuming *E*_0_ = 1 and *E*_1_ = 0. *C* and *h* were then obtained from the slope and y-intercept of the log-linearized data using the scipy.stats.linregress module in Python 2.7. While CI assumes the drug effect is scaled between (0, 1), when this is not the case, a poor fit results (Figs. 6 and S7C, D). All data points with percent viability >1 were excluded from the CI fit because the median-effect equation becomes complex in those cases. For some drugs, this left too few points to fit a line, such that CI was undefined. In other cases, the fitted value for *h* was <0, a physically unrealistic result; therefore, those combinations were also considered undefined. After that, CI was calculated using Eq. (5).

As with Loewe, we apply a $-{{{{\mathrm{log}}}}\,}_{10}$ transformation to scale CI synergy such that $-{{{{\mathrm{log}}}}\,}_{10}({{{\mathrm{CI}}}})\; > \; 0$ the combination is synergistic and if $-{{{{\mathrm{log}}}}\,}_{10}({{{\mathrm{CI}}}})\, < \, 0$ the combination is antagonistic.

#### GPDI model

The GPDI model fitting algorithm was developed in Python by the authors based on description from^24^. Fits for the single drug parameters (*C*1, *C*2, *h*1, *h*2, *E*1, *E*2) were based on the single dose-response data alone (See Table S5 for parameter translation). Fitting for the parameters (*INT*_21_, *INT*_12_, *C*_*INT*,12_, *C*_*INT*,21_) was done using *curve*_*fit* function in python in either the Loewe or Bliss version of GPDI (see Supplemental Section 4.5). For all conditions (*h*_*INT*,12_ = *h*_*INT*,21_ = 1) as was asserted in the original paper.

### Drug combination database analysis

Initial possible matching drug names between the in vitro experiments and the Drug Combination Database (DCDB) were determined using fuzzy string matching in Python (https://github.com/seatgeek/fuzzywuzzy v0.17.0). Drugs which had a sorted token ratio of 85 were initially included. Of the 427 drugs, there were 172 single drug matches. Matches included structural analogs. See *matching*_*drug*_*names*−11−29−2019_*final*.*csv* for complete matching list. Of these matching drugs, there were 126 tested combinations in clinical trials according to DCDB. Outliers in the synergy calculation were considered values 1.5 times the interquartile range (*Q*1−*Q*3) above or below Q1 or Q3 respectively.

### Reporting summary

Further information on research design is available in the Nature Research Reporting Summary linked to this article.

## Supplementary information

Supplementary Information

Description of Additional Supplementary Files

Supplementary Code 1

Reporting Summary

## Data Availability

The datasets analyzed in this study were obtained from publicly available sources with DOIs Mott (10.1038/srep13891, Figs. 3, 6, S4, S6, and S9), O’Neil (10.1158/1535-7163.MCT-15-0843, Figs. 3–4 and S4–S9), Holbeck (10.1158/0008-5472.CAN-17-0489, Figs. 7, S6, and S9), Tan (10.1038/nbt.2391, Fig. S6), and Cokol (10.1038/msb.2011.71, Figs. S6 and S9). Clinical trial data was collected from the Drug Combination Database (DCDB) (10.1093/database/bau124, http://public.synergylab.cn/dcdb/, Figs. S9–S10). The synergy datasets generated in this study are available in a repository at https://bitbucket.org/meyerct1/musyc_theory/.

## Code availability

All code required for recreating manuscript analyses from the MuSyC fits are available for review in the repository https://bitbucket.org/meyerct1/musyc_theory/. A web application to calculate MuSyC parameters is available at https://musyc.lolab.xyz/. The code for the interactive Jupyter notebook demonstration of MuSyC is available at https://github.com/djwooten/natcomms-musyc2021/.
