# Supplementary material for: MuSyC is a consensus framework that unifies multi-drug synergy metrics for combinatorial drug discovery
Source: Nat Commun. 2021 Jul 29;12:4607. doi: 10.1038/s41467-021-24789-z (PMC8322415; doi:10.1038/s41467-021-24789-z)
Supplement: Supplementary file 1 — Supplementary Information [file 41467_2021_24789_MOESM1_ESM.pdf]

MuSyC is a Consensus Framework That Unifies Multi-Drug Synergy Metrics for Combinatorial Drug Discovery: Supplemental Materials

Contents

1 Supplement Figures **2**

2 Relationships between MuSyC and the MSP and DEP **17**
2.1 Multiplicative Survival Principle 17
2.2 Dose Equivalency Principle 18

3 Relationships between different synergy frameworks **18**
3.1 Combination Index 18
3.2 Effective dose model (Zimmer et. al.) 18
3.3 ZIP 19
3.4 BRAID 20
3.5 General Pharmacodynamic Interaction Model 21
3.6 Highest Single Agent 22
3.7 2D Hill PDE 22

4 Percent Affect vs Percent Effect **23**
4.1 MSP 23
4.2 Combination Index 23

5 Sham Compliance of Synergy Frameworks **24**
5.1 Sham Compliance of ZIP 24

6 MuSyC statistically distinguishes efficacious and non-efficacious drug combinations in clinical trials based on *in vitro* combination screens. **25**

7 Proof of boundary behavior of the 2D Hill equation **25**

8 MuSyC Web Application **26**
8.1 Data format 26
8.2 Usage 26
8.2.1 Create an account 26
8.2.2 Create a dataset 26
8.2.3 View results 26

9 Interactive MuSyC Jupyter Notebook **26**

1 Supplement Figures

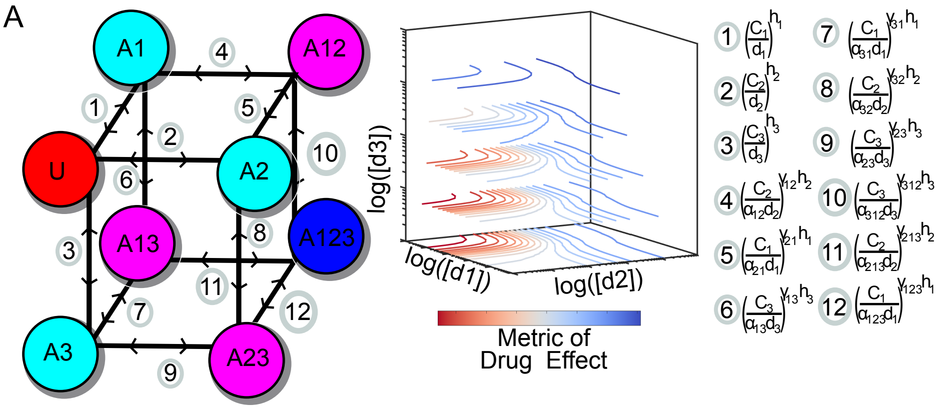

Figure S1: **Extension of MuSyC to combinations of three drugs.** A) Following the cubic geometry of Figure 1B (main text), combinations of 3 drugs result in a cube. Numbered notation next to each edge corresponds to the ratio of the connected corners at equilibrium for the boundary conditions. For example, edge #10 annotation means $\frac{A_{123}}{A_{12}} \to \left(\frac{C_3}{\alpha_{23} d_3}\right)^{\gamma_{312} h_3}$ as doses $d_1 \to$ inf, $d_2 \to$ inf. For combinations of 4 drugs, the geometry is a tesseract. In general, MuSyC can describe combinations N-drug combinations by considering 2^N possible states with transitions defining the edges of an N-dimensional hypercube. Dose-response surfaces generalize to N-dimensional scalar functions. In the most general case, for N drugs there are $2^N - N - 1$ distinct β parameters (one for each state characterized by the action of at least 2 drugs), and $N \cdot \left(2^{N-1} - 1\right)$ distinct α and γ parameters (one for each edge, excluding edges connected to the undrugged state, which correspond only to single-drug potency and cooperativity). Thus, MuSyC can account for higher-order synergies (e.g., synergy that emerges from a combination of three drugs, but is not evident in any pairwise combination of those drugs), however the rapid growth of the number of synergy parameters with N suggests that significant quantities of data, or confident knowledge of pairwise synergies, would be needed to measure such higher-order synergies.

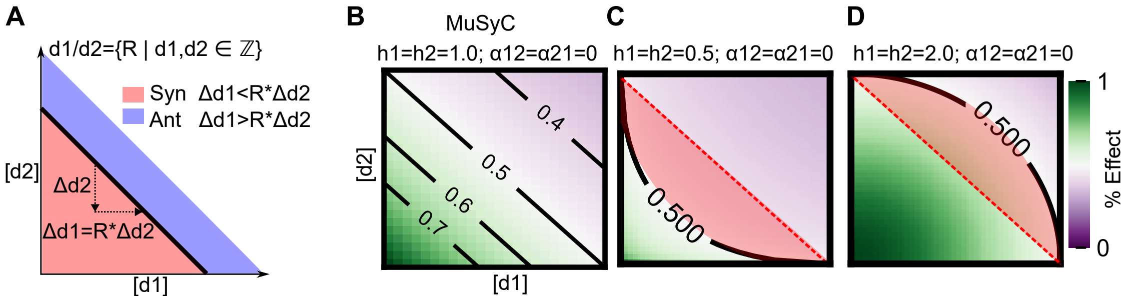

Figure S2: **Hill-slope and nonlinear isoboles.** A) Isoboles are defined as contours of equal effect. Along these lines, all dose pairs result in the same effect. The linear isoboles of Loewe result from asserting that for a given effect E—achievable either by dose d_1 of Drug 1 alone, or dose d_2 of Drug 2 alone—there is a constant ratio $R = \frac{d_1}{d_2}$ such that using Δd_2 less of Drug 2 can always be compensated for with $\Delta d_1 = R\Delta d_2$ more of Drug 1 to achieve the same effect. Synergy (Syn, red) occurs when less of one drug is required to compensate for a decrease in the other than expected. Likewise, antagonism (Ant, blue) occurs when more of one drug is required. B) MuSyC's isoboles become linear under the constraint that the drugs are mutually exclusive ($\alpha_{12} = \alpha_{21} = 0$) and $h_1 = h_2 = 1$. Additionally, the slope of the line (R) in MuSyC is equal to the ratio of the two drugs' EC50 ($-\frac{C2}{C1}$) (See eq. 27 in Supplemental Section Relationship between MuSyC and the MSP and DEP). C) When $h < 1$, the MuSyC isoboles bend inward. As a result, DEP-based frameworks (e.g. Loewe and CI), which assume linear isoboles, will quantify the red shaded region (between the straight, dotted, diagonal line, and the curved line below it) as synergistic thereby overestimating the synergy. D) Conversely, when $h > 1$, MuSyC isoboles bend outward causing DEP-based frameworks to overestimate antagonism. For clarity, panels C and D have been replicated in Figures 6A (main text), S8A.

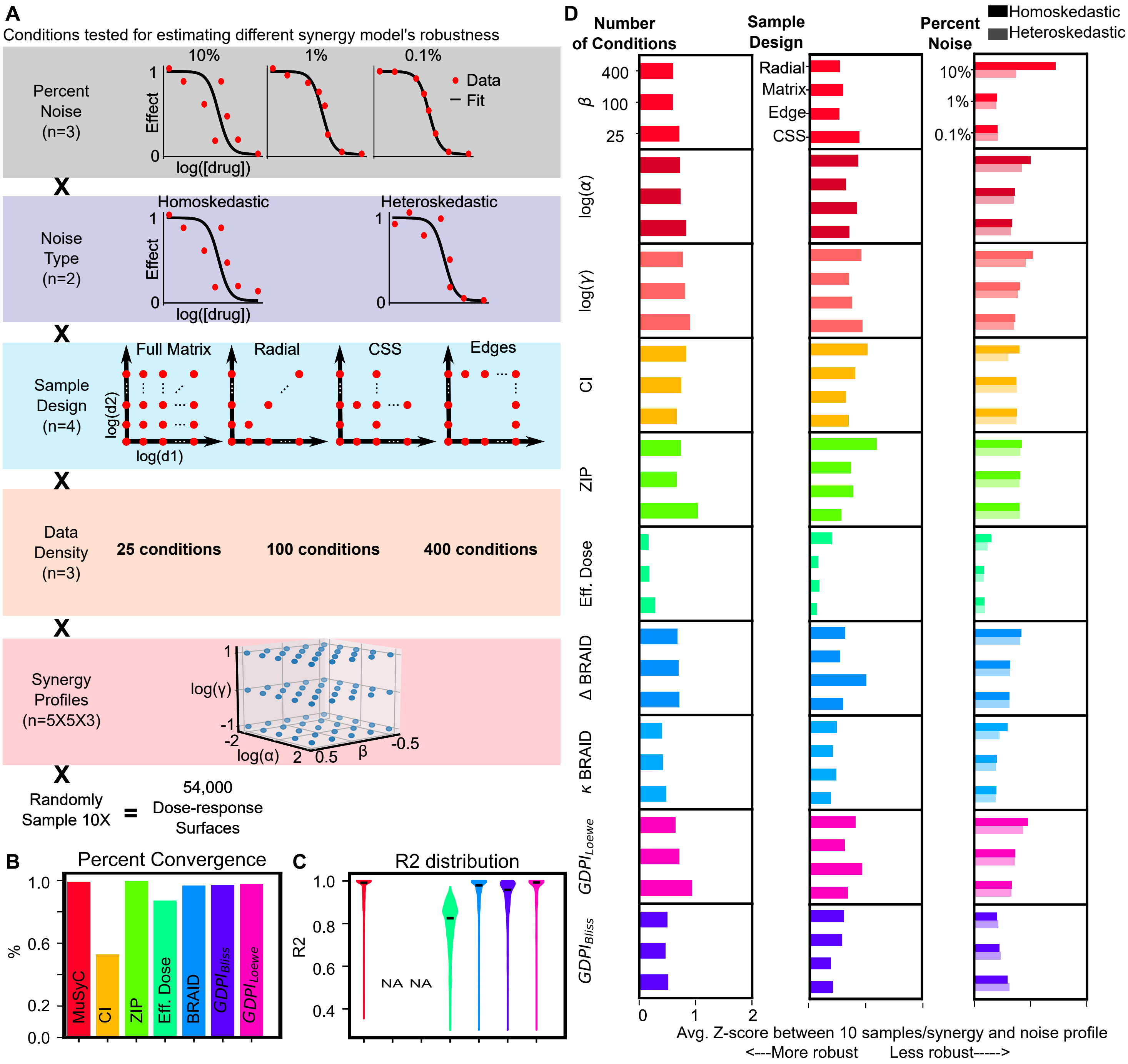

Figure S3: **Fit algorithm robustness to different noise profiles.** A) Varying conditions considered included percent noise, noise type, sampling design, data density, and synergy profile. Each condition was randomly sampled 10 times corresponding to 54,000 synthetic datasets. For sample design, the data density was distributed evenly such that a Full Matrix sample with 100 conditions has 10 single doses per drug while the CSS or the Edge sampling with 100 conditions has 25 single dose samples (100/4 lines). All surfaces have the following single drug parameters ($h1 = h2 = 1$,$E0 = 1$,$E1 = E2 = 0.334$,$C1 = C2 = 1e-5$). Doses were sampled between ($1e-10$,1) using a log sampling. B) Percent of conditions for which the fit converged out of 54,000. Only parameterized models of drug synergy were compared. Fits with $R^2 < 0.3$ were considered to not converge. MuSyC and ZIP converge most frequently (99%) while CI and Effective Dose (Eff. Dose) converge less frequently as they both assume the maximal and minimal effect of the drug is (0,1). C) Distributions of R^2 values for each method. ZIP and CI synergy are calculated as the deviation at each point from the null and therefore have no R^2 value. MuSyC has the highest mean R^2 value (0.95) followed by BRAID and GPDI(Loewe) at (0.94). D) Average Z-score between each 10 matched random samples is used as a measure of robustness. Lower average Z-score indicates less variation in synergy calculation as a result of noise. MuSyC fits are not significantly impacted by the density of the data (first column). CSS sampling increases the uncertainty of β (second column) which is expected because the point used to define β (max(d1),max(d2)) is absent. Overall, the Effective Dose (Eff. Dose) was the most robust. For all methods, heteroskedastic distribution of noise is better fit than homoskedastic, a difference that becomes more exaggerated at higher levels of noise.

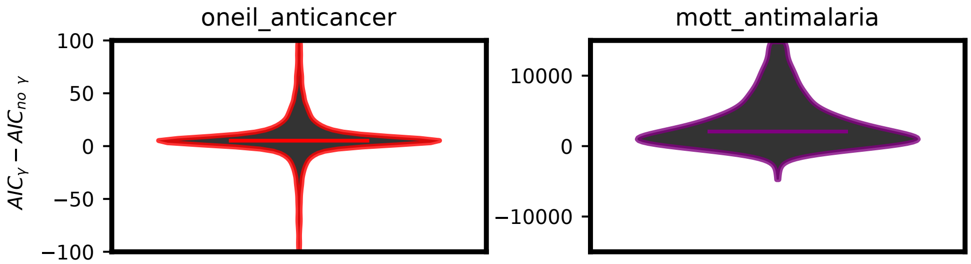

Figure S4: **Model selection prefers simpler MuSyC model.** Difference in the Akaike Information Criterion (AIC) values for MuSyC models including fitting synergistic cooperativity (γ_{12} and γ_{21}) or fixing γ to 1 thereby reducing the parameter count by 2. Models which minimize AIC are preferred. In most cases the simpler model is preferred. The mean $AIC_\gamma - AIC_{no\gamma}$ for anti-cancer and anti-malarial datasets was 8 and 3312, respectively. The percent of combinations for which the model including γ had a lower AIC value was 18% and 5%, respectively.

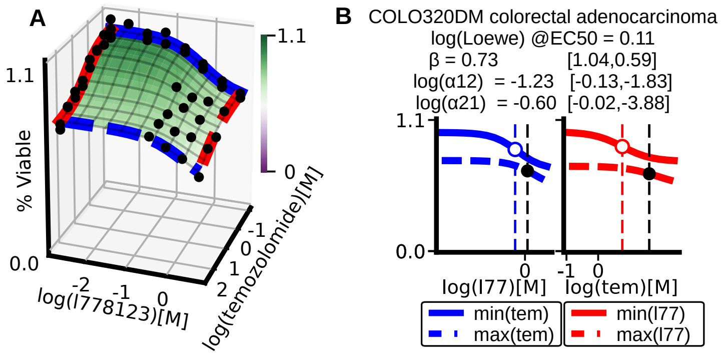

Figure S5: **Loewe conflates synergistic potency and efficacy.** A) Fitted dose response surface of the combination of L778123 (a dual farnesyl and geranylgeranyl transferase inhibitor) and temozolomide (DNA alkylating agent) in COLO320DM cell lines[1]. B) This combination is synergistically efficacious but antagonistically potent, and is an example where Loewe misses antagonistically potent interactions.

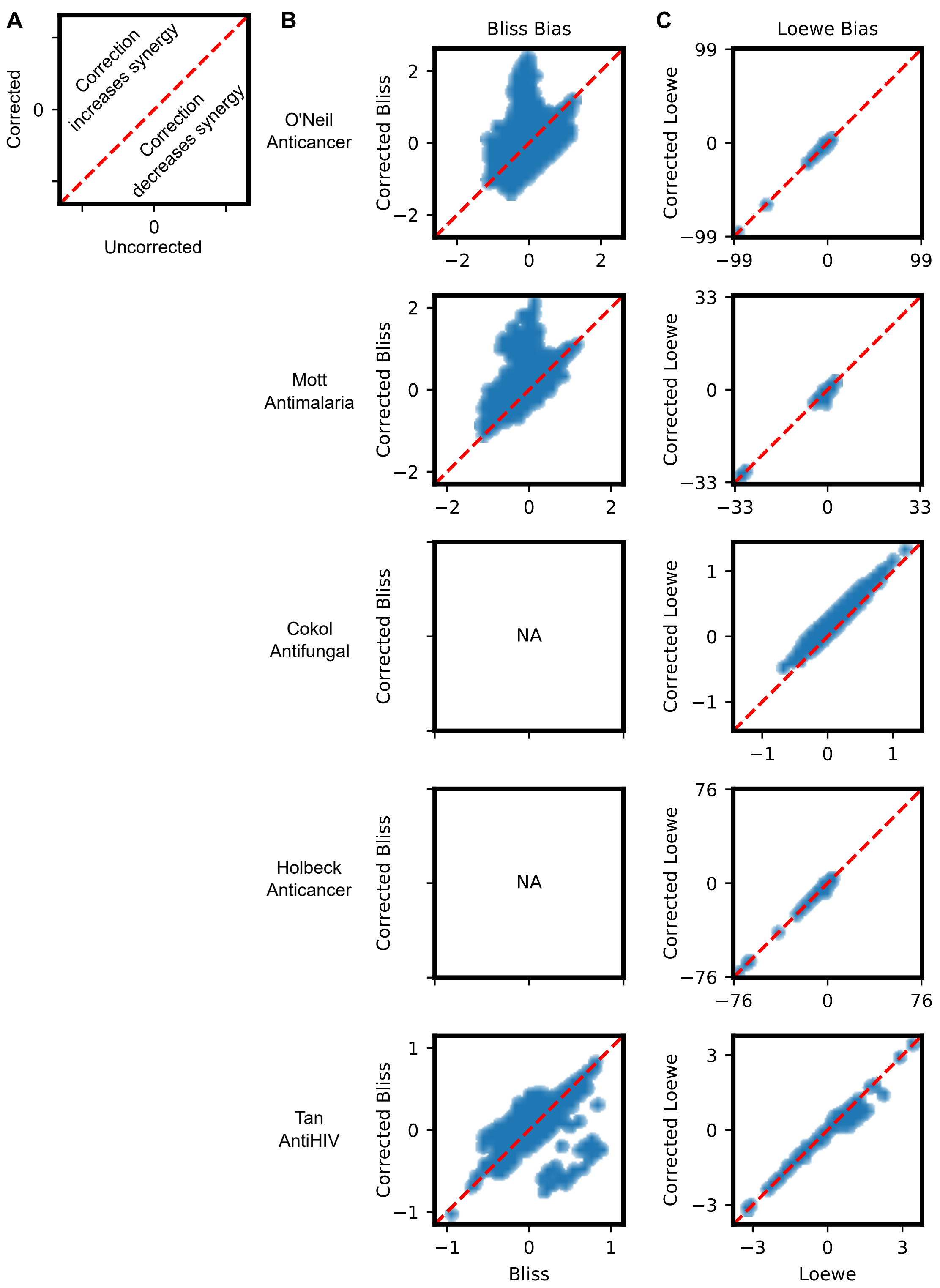

Figure S6: **Impact of bias in Bliss and Loewe.** A) Schematic for interpreting bias figures. Synergy as calculated by Bliss or Loewe is plotted on the x-axis. Subtracting the MuSyC-estimated bias (calculated for each data point) results in a corrected value of Bliss or Loewe synergy, plotted on the y-axis. We consider efficacy-bias for Bliss (see Figure 4B, main text) and Hill-slope bias for Loewe (see Figure 5C, main text). Points along the line $y = x$ have no bias. Points above the line indicate the uncorrected metric underestimated synergy. Points below the line indicate the uncorrected metric overestimated synergy. B) Bliss efficacy-bias plots. Bliss cannot be calculated for the Cokol and Holbeck datasets due to their effect metrics (Table 3, main text). There is substantial deviation from the red line indicating the magnitude of the efficacy-bias is comparable to the magnitude of uncorrected Bliss. C) Loewe Hill-bias plots. While, there is less deviation from the red line than Bliss, we observe whole datasets (e.g., Cokol et. al) for which the bias artificially reduces Loewe synergy.

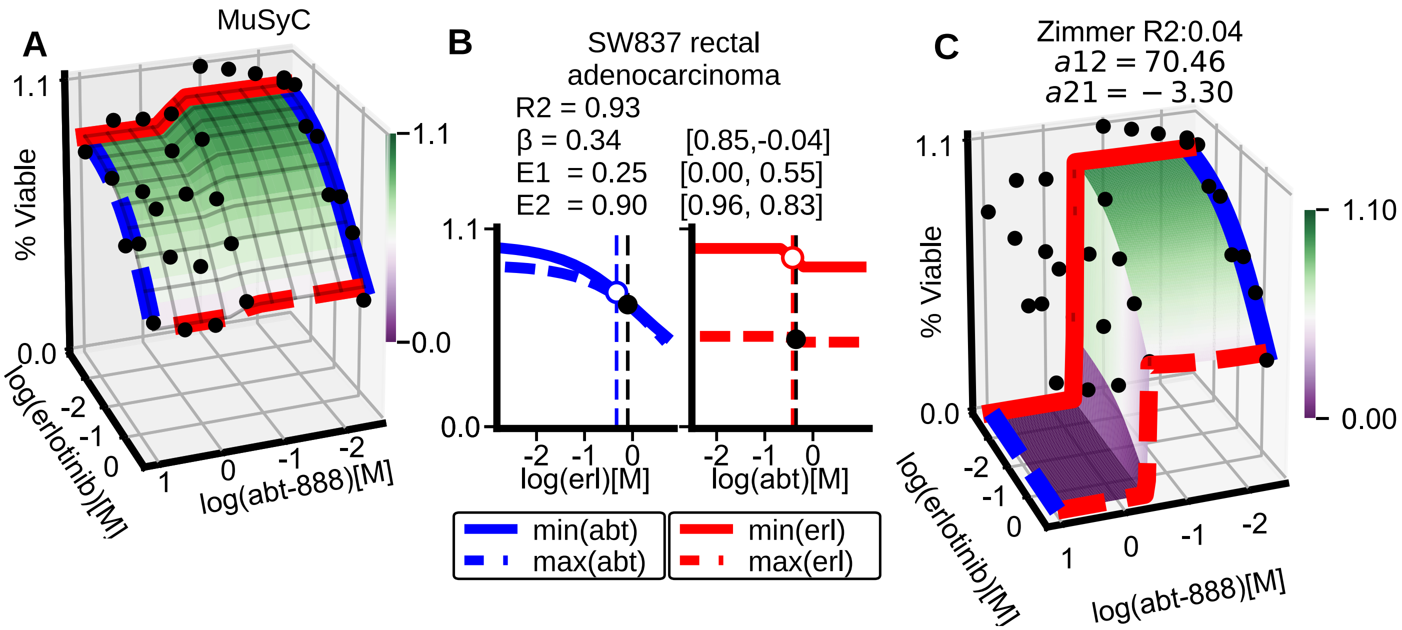

Figure S7: **Drugs with weak monotherapy maximum effect can cause problems for some synergy models.** A) Dose-response surface for combination erlotinib and abt-888 in SW837 cells[1]. B) MuSyC parameter fits showing weak $E_{max} = E_2 = 0.9$ for abt-888. C) Dose response surface according to Effective Dose model, which enforces $E_0 = 1$ and $E_1 = E_2 = 0$, which leads to poor fit to data.

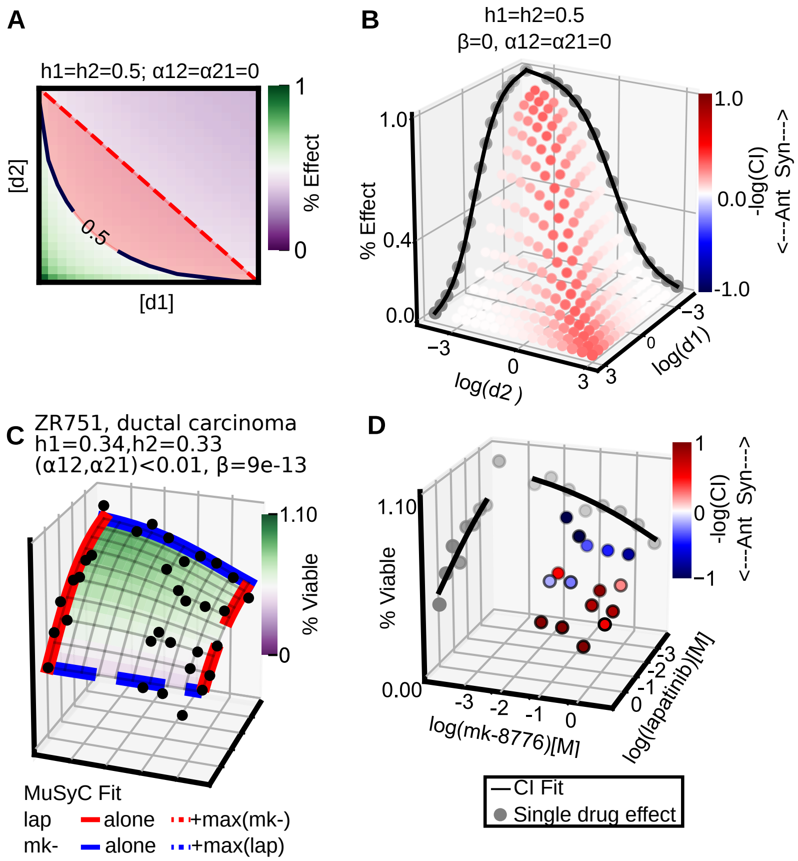

Figure S8: **Small Hill-slope biases Combination Index toward synergy.** A) MuSyC predicts nonlinear isoboles (bottom) when $h < 1$. Here, CI would erroneously assess dose pairs in the red shaded region (between the straight, dotted diagonal line, and the solid curve beneath it) as synergistic. B) An example, synthetic two-drug dataset where this Hill dependent bias is apparent ($h_1 = h_2 = 0.5$,$E_0 = 1$, $E_1 = E_2 = 0.0$, $C_1 = C_2 = 1$, $\alpha_{12} = \alpha_{21} = 0$). This trend is most apparent near $d_1 \approx d_2$. C) MuSyC fit of the combination of mk-8776 (CHK1 inhibitor) and lapatinib (EGFR inhibitor) in ZR751 (luminal B breast cancer)[1]. D) Color indicates CI of the combination from (C), and shows the predicted CI bias (synergy along diagonal due to $h < 1$). Because $E_0 = 1$,$E_1 = E_2 = 0$, and (α_{12},α_{21})<0.0001, the difference between MuSyC and CI is due to the Hill slope error in CI.

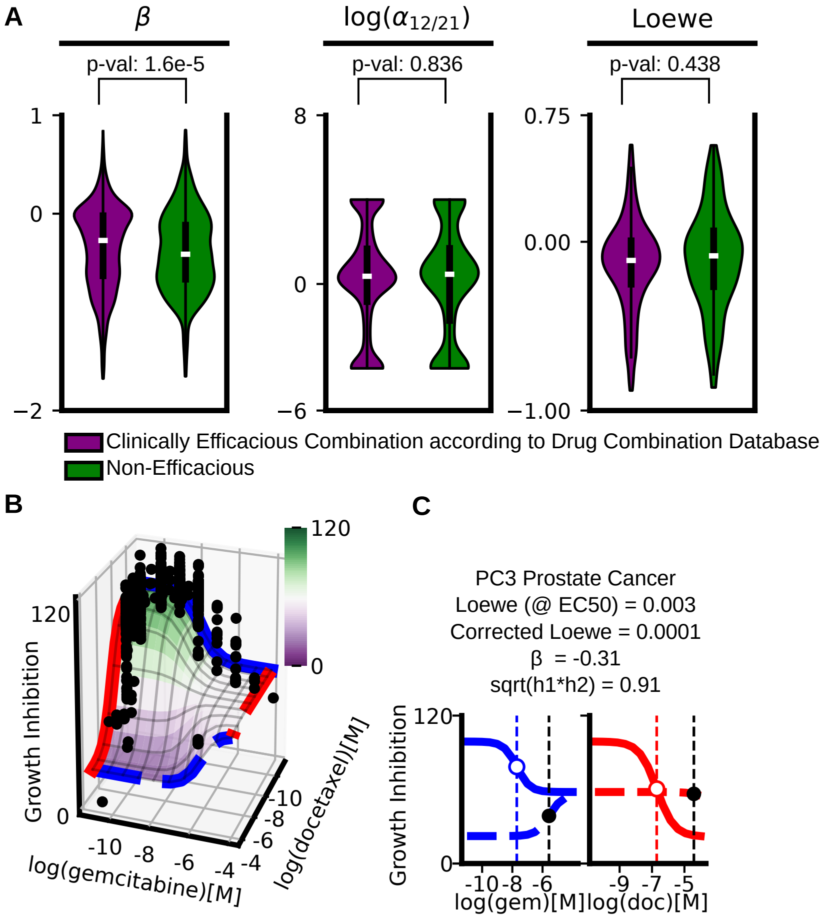

Figure S9: **MuSyC distinguishes Non-efficacious and Efficacious clinical combinations based on *in vitro* screening data.** A) Distributions of synergistic efficacy (β), synergistic potency ($log(\alpha_{12/21})$), and Loewe additivity from *in vitro* measurements of clinical combinations in DCDB. Data were aggregated from [1–4], see Figure S10 for details. Only combinations with two drugs were considered. p-values were calculated by one-sided t-test (null hypothesis $green > purple$) after outliers were removed. The white marks indicate the means, and black boxes indicate the 25th and 75th percentiles. Sample count for p-value calculations for β, $log(\alpha_{12/21})$, and Loewe are (n=2538, 5266, 1198) efficacious combinations (purple) and (n=796, 1620, 315) non-efficacious combinations (green), respectively. B) Combination of gemcitabine (nucleoside analogue) and docetaxel (tubulin stabilizer) in prostate cancer PC3 cells (ALMANAC dataset [2]). C) The combination in (B) is antagonistically efficacious (β=-0.31), but synergistic by Loewe due in part to the Hill bias ($\sqrt{h_1 \cdot h_2} < 1$, Figure 5, main text).

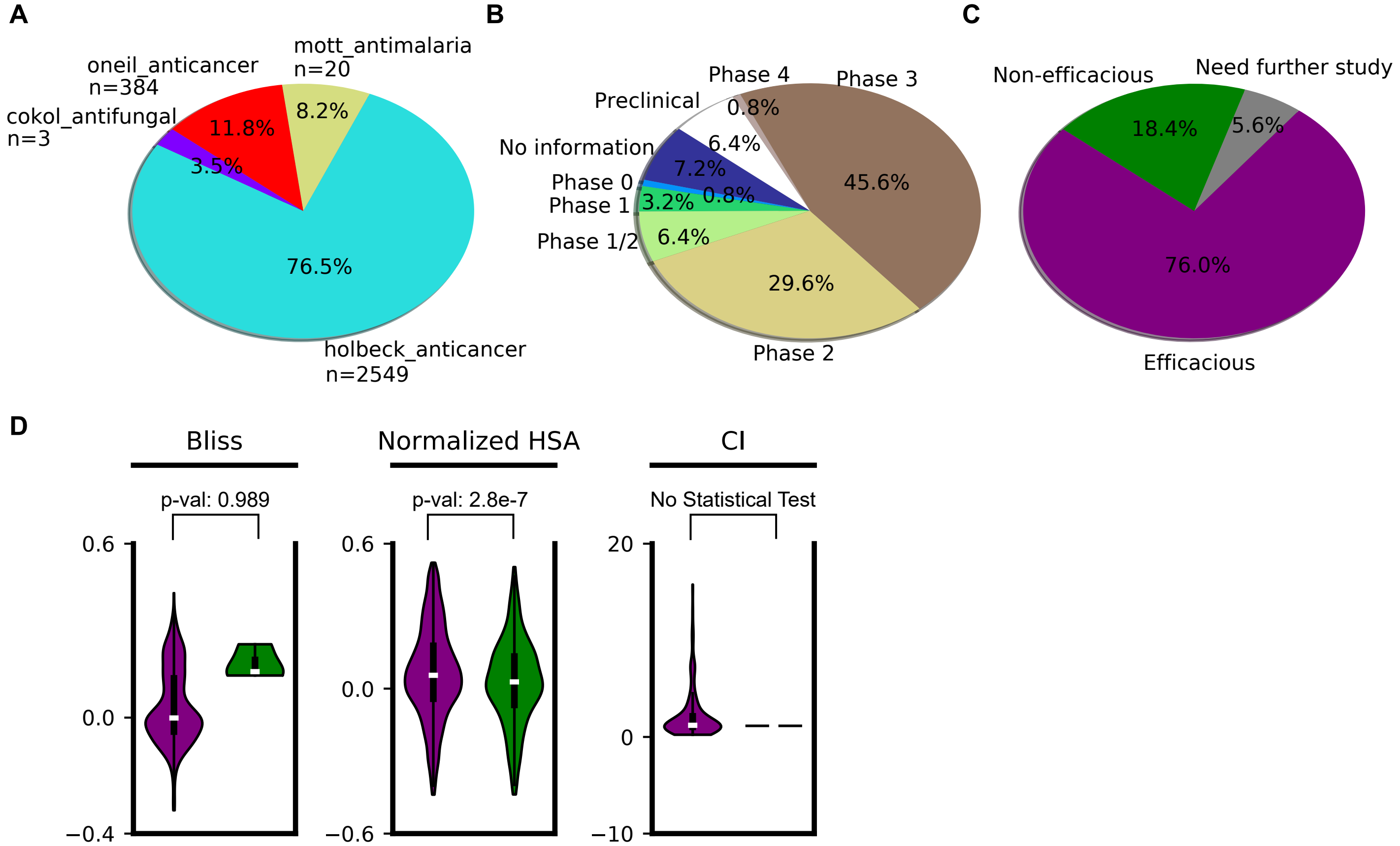

Figure S10: **Clinical drug combinations with matching *in vitro* data**. A) Five *in vitro* drug combination datasets (Table 3, main text) were cross-referenced with clinical drug combinations in DCDB. Total there were 126 matching combinations tested in 2,956 samples (n in chart). The ALMANAC screen (holbeck_anticancer)[2] contains the 77% of the 126 combinations. B) Of the clinically-tested drug combinations, 46% reached a Phase 3 or Phase 4 clinical trial and over 85% were at least Phase 1. C) Clinical combinations are annotated as efficacious according to DCDB. D) Distribution of Bliss, HSA, and CI for the efficacious and non-efficacious combinations. Bliss and CI cannot be calculated for any of the holbeck_anticancer or cokol_antifungal due to the measured drug effects not being a percentage in the range (0,1) resulting in only 3 and 1 combinations, respectively, which are non-efficacious. The classic HSA method depends on the assay scale. However, if HSA is normalized by dividing the effect metric range, similar to MuSyC's β, there is a statistical difference (middle panel, p-value calculated by one-sided t test, assuming normality; green>purple). Sample count for p-value calculations for Bliss, HSA, and CI are (n=403, 2497, 265) efficacious combinations (purple) and (n=3, 772, 1) non-efficacious combinations (green), respectively. The white mark shows the median. The thick portion of the vertical bar running through the middle of each violin plot shows the interquartile range, while the thin portion shows the minimum to maximum range.

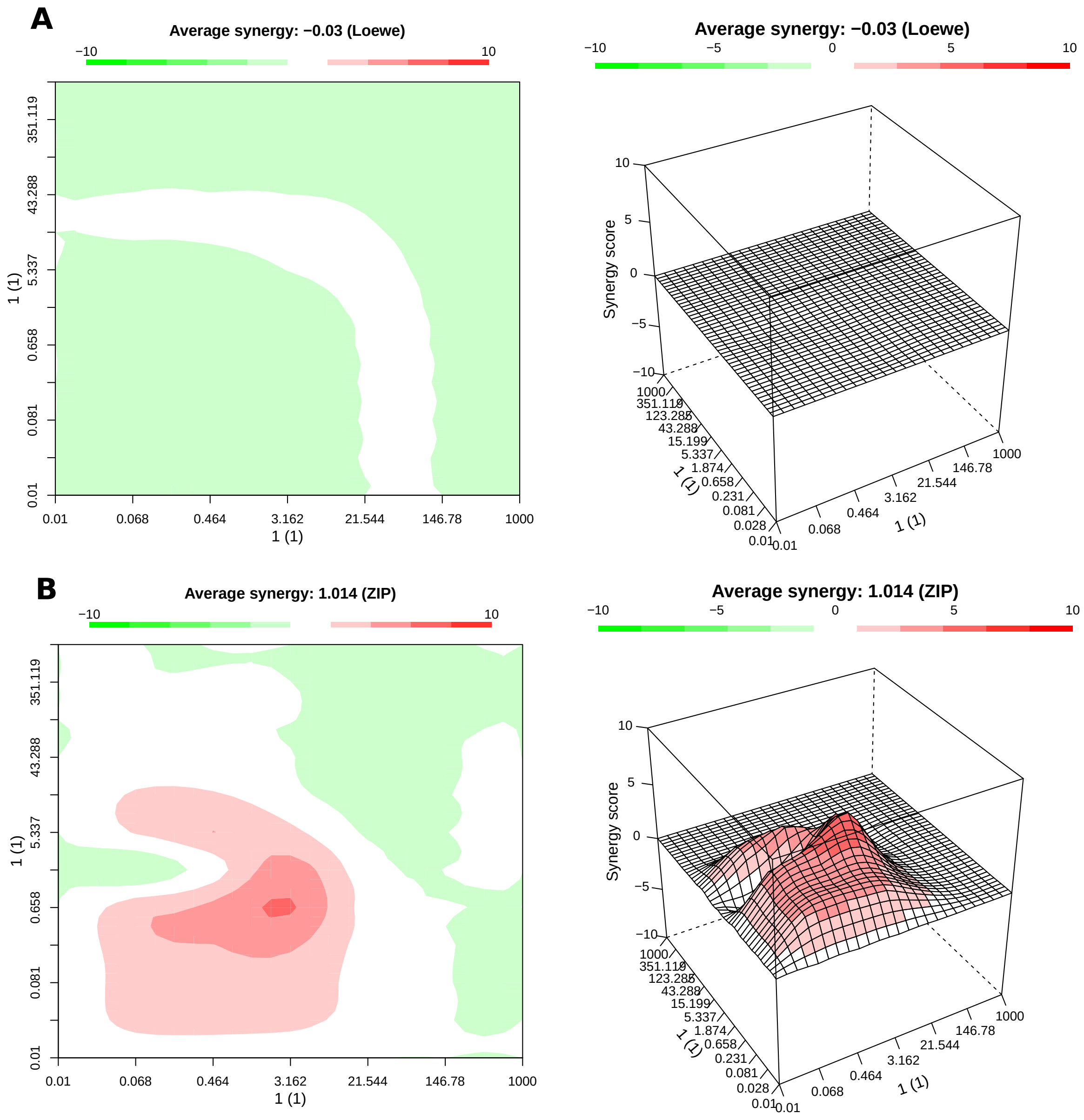

Figure S11: **Sham compliance of Loewe and ZIP synergy.** A) Loewe synergy calculated by synergyfinder[5] for a synthetic sham dose-response surface with $h = 2$. Loewe correctly identifies the combination as additive. The left shows the default heatmap generated by synergyfinder, while the right shows the default surface plot. Both the left and right represent the same data. B) ZIP quantifies synergy or antagonism at several concentrations for the sham dataset.

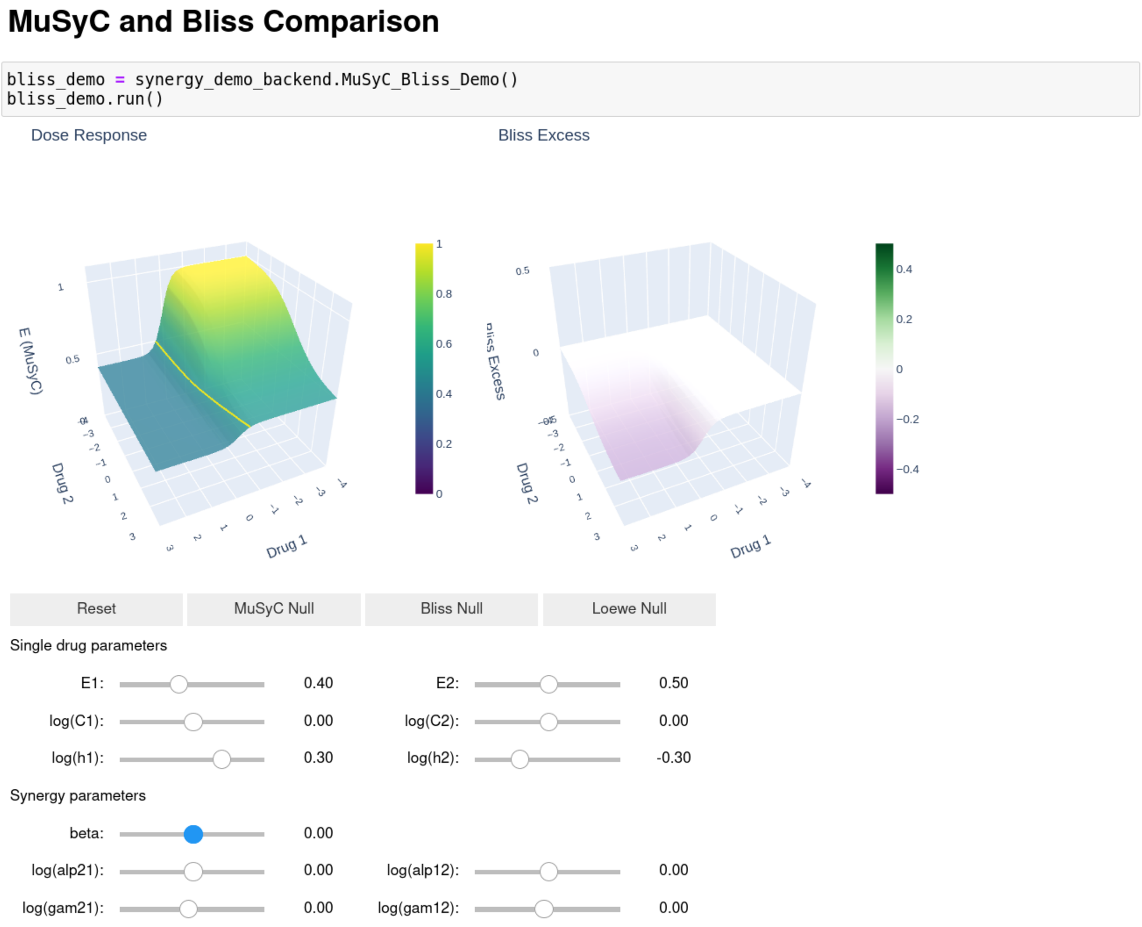

Figure S12: **Demo: Bliss shows antagonism for medium-efficacy drugs.** Screenshot of the interactive Jupyter Notebook in File S1, and hosted online at `https://mybinder.org/v2/gh/djwooten/natcomms-musyc2021/HEAD?filepath=demo.ipynb`. On the left is the MuSyC dose-response surface corresponding to the parameters given by the sliders below. On the right is the Bliss excess synergy landscape. Because $E_1 = 0.4$ and $E_2 = 0.5$, Bliss expects the combination to achieve $E_3 = E_1 \cdot E_2 = 0.2$. However, because $\beta = 0$, we have $E_3 = min(E_1, E_2) = 0.4$. Because of this, Bliss rates this combination as antagonistic at high doses of both drugs.

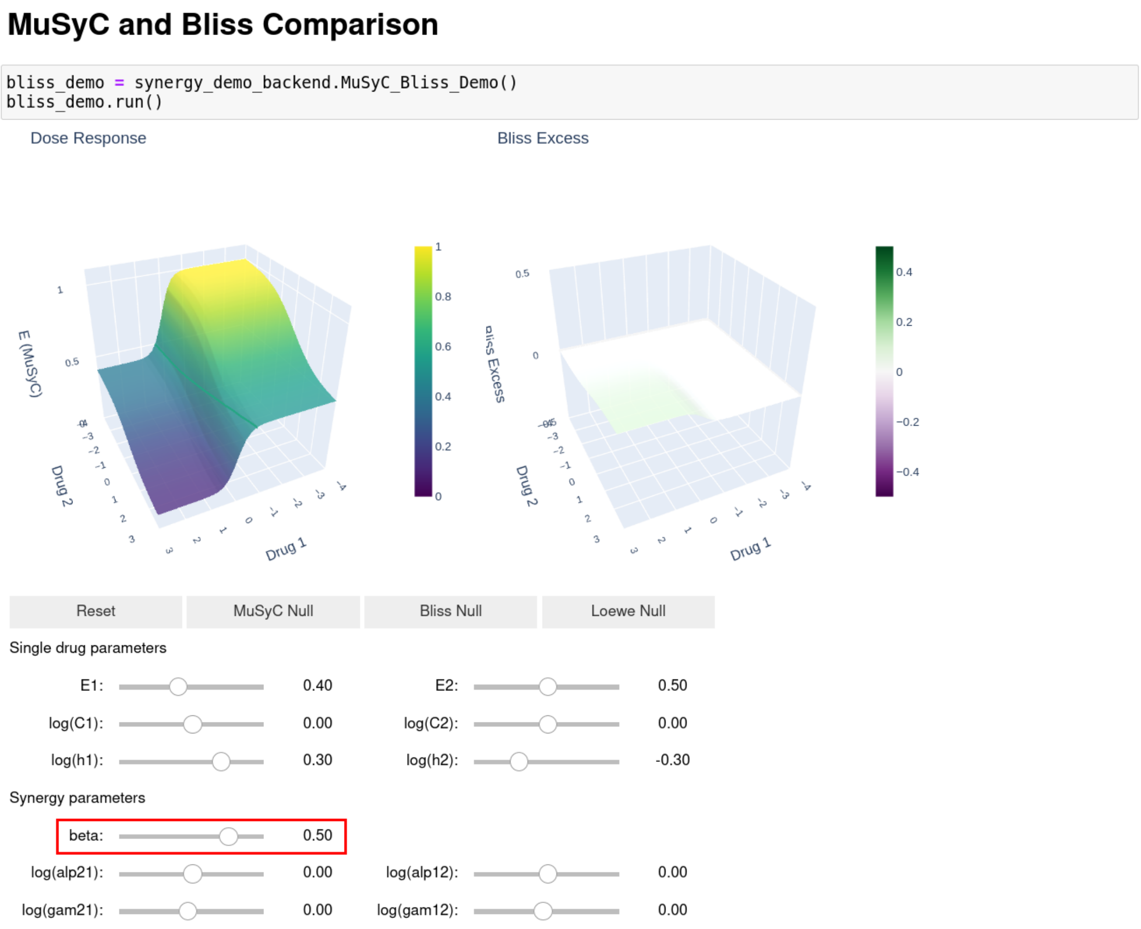

Figure S13: **Demo: Synergistic efficacy may be reflected by Bliss at high doses of both drugs.** Screenshot of the interactive Jupyter Notebook in File S1, and hosted online at https://mybinder.org/v2/gh/djwooten/natcomms-musyc2021/HEAD?filepath=demo.ipynb. On the left is the MuSyC dose-response surface corresponding to the parameters given by the sliders below. On the right is the Bliss excess synergy landscape. Because $E_1 = 0.4$ and $E_2 = 0.5$, Bliss expects the combination to achieve $E_3 = E_1 \cdot E_2 = 0.2$. However, because $\beta = 0.5$, this combination has $E_3 = 0.1$. Because of this, Bliss rates this combination as synergistic at high doses of both drugs. In general, adjusting the slider for beta affects both the dose response surface and the Bliss excess landscape at high doses of each drug.

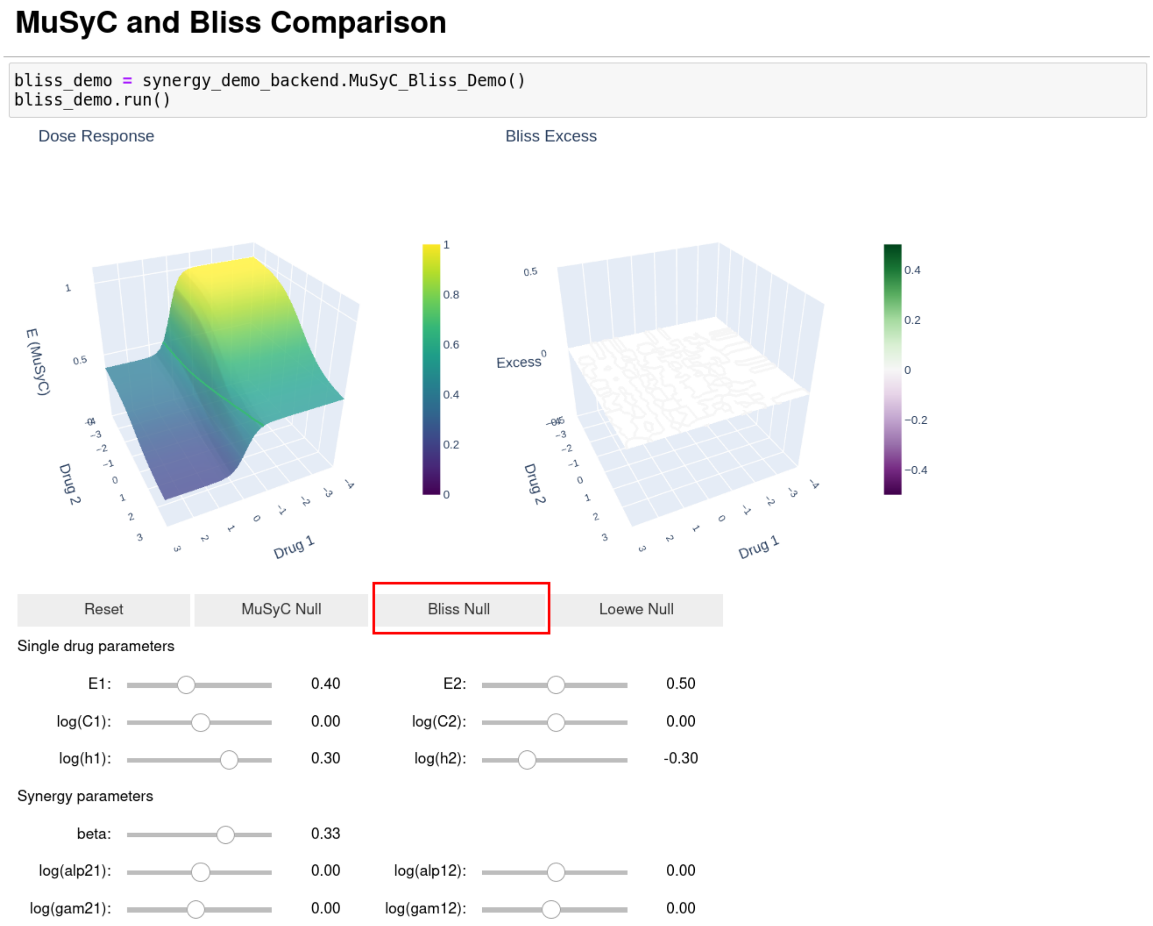

Figure S14: **Demo: MuSyC can reproduce Bliss null model.** Screenshot of the interactive Jupyter Notebook in File S1, and hosted online at `https://mybinder.org/v2/gh/djwooten/natcomms-musyc2021/HEAD?filepath=demo.ipynb`. On the left is the MuSyC dose-response surface corresponding to the parameters given by the sliders below. On the right is the Bliss excess synergy landscape. Clicking the "Bliss Null" button highlighted in red will adjust the MuSyC synergy sliders to have $\log(alpha) = \log(gamma) = 0$ and $E_3 = E_1 \cdot E_2 = 0.2$ (note: E_3 is controlled by adjusting the beta slider, such that $E_3 = min(E_1, E_2) - \beta \cdot (E0 - min(E_1, E_2))$, where $E_0 = 1$ is a constant in the demo). With these settings, as can be seen on the right, the MuSyC dose-response surface matches the Bliss null model, so that this combination has 0 synergy by Bliss at every dose. In general, adjusting the alpha sliders affects both the dose response surface and the Bliss synergy landscape near the EC50 of one drug, and at high doses of the other.

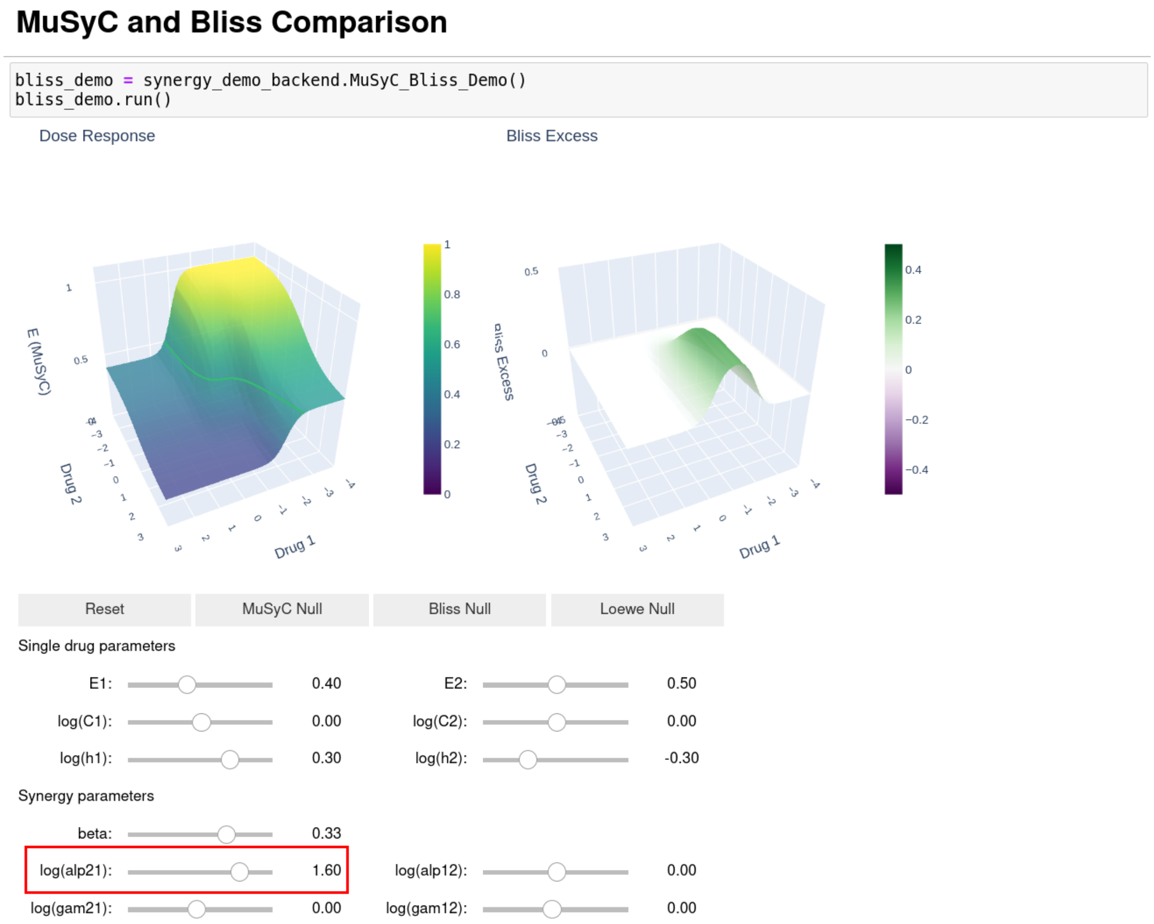

Figure S15: **Demo: Synergistic potency may be reflected near the EC50 of the drug whose potency is synergistically improved.** Screenshot of the interactive Jupyter Notebook in File S1, and hosted online at `https://mybinder.org/v2/gh/djwooten/natcomms-musyc2021/HEAD?filepath=demo.ipynb`. On the left is the MuSyC dose-response surface corresponding to the parameters given by the sliders below. On the right is the Bliss excess synergy landscape. Starting from the Bliss null model (obtained by pressing the "Bliss Null" button), and adjusting the slider for alpha21 highlighted in red, we see that this parameter manifests as Bliss synergy at doses of Drug 1 near its EC50, and at high doses of Drug 2. This is because the parameter alpha21 describes how the presence of Drug 2 (meaning Drug 2 must be administered at high enough doses to be impactful) affects the potency of Drug 1 (meaning this is most apparent near the EC50 of Drug 1).

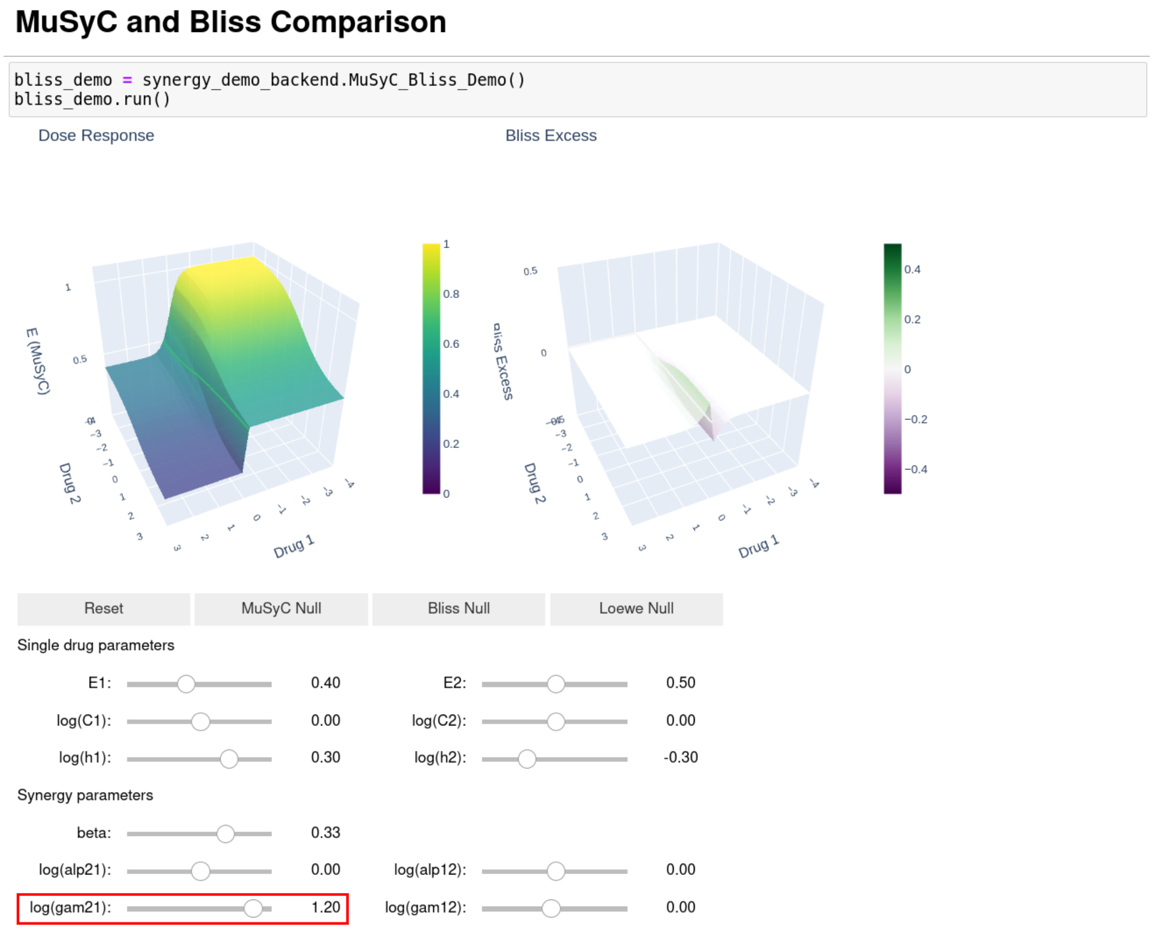

Figure S16: **Demo: Synergistic cooperativity may be reflected near the EC50 of the drug whose Hill-slope is synergistically altered.** Screenshot of the interactive Jupyter Notebook in File S1, and hosted online at https://mybinder.org/v2/gh/djwooten/natcomms-musyc2021/HEAD?filepath=demo.ipynb. On the left is the MuSyC dose-response surface corresponding to the parameters given by the sliders below. On the right is the Bliss excess synergy landscape. Starting from the Bliss null model (obtained by pressing the "Bliss Null" button), and adjusting the slider for gamma21 highlighted in red, we see that this parameter manifests as both Bliss synergy and Bliss antagonism at doses of Drug 1 near its EC50, and at high doses of Drug 2. In general, adjusting the gamma sliders affects both the dose response surface and the Bliss synergy landscape near the EC50 of one drug, and at high doses of the other.

2 Relationships between MuSyC and the MSP and DEP

2.1 Multiplicative Survival Principle

MuSyC matches the Bliss null surface when there is no potency synergy ($\alpha_{12} = \alpha_{21} = 1$), no cooperativity synergy ($\gamma_{12} = \gamma_{21} = 1$), and $E_3 = E_1 \cdot E_2$ (Figure 2A, main text). To show this, let each drug in isolation have a 1D hill response

$$U_i = \frac{1}{1 + \left(\frac{d_i}{C_i}\right)^{h_i}} \tag{22}$$

where U_i reflects the portion of cells unaffected by drug i alone. For the 2D case, when $\alpha_{12} = \alpha_{21} = 1$ and $\gamma_{12} = \gamma_{21} = 1$, each edge in Figure 1B (main text) satisfies detailed balance and therefore the state occupancy is given by

$$A_1 = \left(\frac{d_1}{C_1}\right)^{h_1} U$$

$$A_2 = \left(\frac{d_2}{C_2}\right)^{h_2} U$$

$$A_{1,2} = \left(\frac{d_2}{C_2}\right)^{h_2} A_1$$

Because $U + A_1 + A_2 + A_{1,2} = 1$, the MuSyC mass action model gives

$$U = \frac{1}{1 + \left(\frac{d_1}{C_1}\right)^{h_1} + \left(\frac{d_2}{C_2}\right)^{h_2} + \left(\frac{d_1}{C_1}\right)^{h_1}\left(\frac{d_2}{C_2}\right)^{h_2}} \tag{23}$$

$$A_1 = \frac{\left(\frac{d_1}{C_1}\right)^{h_1}}{1 + \left(\frac{d_1}{C_1}\right)^{h_1} + \left(\frac{d_2}{C_2}\right)^{h_2} + \left(\frac{d_1}{C_1}\right)^{h_1}\left(\frac{d_2}{C_2}\right)^{h_2}}$$

$$A_2 = \frac{\left(\frac{d_2}{C_2}\right)^{h_2}}{1 + \left(\frac{d_1}{C_1}\right)^{h_1} + \left(\frac{d_2}{C_2}\right)^{h_2} + \left(\frac{d_1}{C_1}\right)^{h_1}\left(\frac{d_2}{C_2}\right)^{h_2}}$$

$$A_{1,2} = \frac{\left(\frac{d_1}{C_1}\right)^{h_1}\left(\frac{d_2}{C_2}\right)^{h_2}}{1 + \left(\frac{d_1}{C_1}\right)^{h_1} + \left(\frac{d_2}{C_2}\right)^{h_2} + \left(\frac{d_1}{C_1}\right)^{h_1}\left(\frac{d_2}{C_2}\right)^{h_2}}$$

From this, it is easy to verify that $U = U_1 \cdot U_2$ where $U_1 = 1 - (A_1 + A_{1,2})$ and $U_2 = 1 - (A_2 + A_{1,2}$ which is equivalent to the Bliss Independence null model.

Furthermore, given $E_0 = 1$

$$E = U + A_1E_1 + A_2E_2 + A_{1,2}E_3 \tag{24}$$

We define $\overline{U_i}$, $\overline{A_i}$, and $\overline{E_i} = \overline{U_i} + \overline{A_i}E_i$ to be the fraction of unaffected cells, fraction of affected cells, and observed effect for treatment due to the single drug i, as described by equation 22. The overline distinguishes affects attributable to each drug, such that $\overline{A_1}$ includes cells affected either by drug 1 alone, or by both drug 1 and drug 2, while A_1 only includes cells affected by drug 1, but not drug 2 (i.e., $\overline{A_1} = A_1 + A_{1,2}$). Then

$$\begin{aligned}\overline{E_1} \cdot \overline{E_2} &= \left[\overline{U_1} + (1 - \overline{U_1}E_1)\right]\left[\overline{U_2} + (1 - \overline{U_2}E_2)\right] \\ &= \overline{U_1U_2} + E_1(\overline{U_2} - \overline{U_1U_2}) + E_2(\overline{U_1} - \overline{U_1U_2}) + E_1E_2(1 - \overline{U_1})(1 - \overline{U_2})\end{aligned}$$

From 23, we know $U = \overline{U_1} \cdot \overline{U_2}$, and $A_{1,2} = \overline{A_1} \cdot \overline{A_2}$, leading to

$$= U + E_1(\overline{U_2} - U) + E_2(\overline{U_1} - U) + E_1E_2A_{1,2}$$

Similarly, it is simple to show $A_1 = \overline{U_2} - U$, and similarly for A_2

$$= U + E_1A_1 + E_2A_2 + E_1E_2A_{1,2}$$

If $E_3 = E_1 \cdot E_2$, then this is equivalent to equation (24). Therefore, given $\alpha_{12} = \alpha_{21} = 1$, $\gamma_{12} = \gamma_{21} = 1$, $E_0 = 1$, and $E_3 = E_1 \cdot E_2$, MuSyC predicts $\overline{E_1} \cdot \overline{E_2} = E$. Thus, while Bliss was derived purely within the scope of "percent affected", MuSyC shows that the Bliss model may be appropriately extended to any measure of effect for which $E_0 = 1$ and effects are expected to be multiplicative. Nevertheless, for effects which do not satisfy these criteria, the Bliss model cannot be reliably used, while MuSyC may still be used for arbitrary effects.

2.2 Dose Equivalency Principle

The DEP defines asserts an expectation that for a given effect E, achievable either by dose d_1 of Drug 1 alone, or dose d_2 of Drug 2 alone, there is a constant ratio $R = \frac{d_1}{d_2}$ such that using Δd_2 less of Drug 2 can always be compensated for by using $\Delta d_1 = R\Delta d_2$ more of Drug 1 to achieve the same effect[6]. This definition leads to the linear isoboles characteristic of the Loewe null model.

Chou and Talalay showed that linear isoboles emerge when the two drugs are mutually exclusive[7], meaning that the double-drugged state ($A_{1,2}$ in Figure 1B, main text) is unreachable. In MuSyC, this requires setting $\alpha_{12} = \alpha_{21} = 0$, which reduces the 2D Hill equation (eq. 15, main text) to

$$(E - E_0) + (E - E_1)\left(\frac{d_1}{C_1}\right)^{h_1} + (E - E_2)\left(\frac{d_2}{C_2}\right)^{h_2} = 0 \tag{25}$$

From this it is easy to see when $h_1 = h_2 = 1$, the equation describes a straight line line, equivalent to the canonical linear isoboles of Loewe Additivity and the CI null models. Further from equation (25), we find the slope of isoble is equal to $-\frac{C_2}{C_1}$ as shown by:

$$\frac{\partial}{\partial E}\left((E - E_0) + (E - E_1)\left(\frac{d_1}{C_1}\right) + (E - E_2)\left(\frac{d_2}{C_2}\right)\right) = 0 \tag{26}$$

$$d_2 = -d_1\frac{C_2}{C_1} - C_2 \tag{27}$$

Therefore the constant R in the statement of the Dose Equivalence Principle is revealed by MuSyC to be equal to the ratio of the two drugs' EC50. There is no dependence on β or γ because those parameters relate to the $A_{1,2}$ state, which is blocked here. For fixed values of E, equation (25) results in linear isoboles only when $h_1 = h_2 = 1$. Thus, given these conditions on α and h, MuSyC reproduces the DEP. However, when $h \neq 1$ nonlinear isoboles result (Figures 6A (main text), S8A), suggesting that DEP is an inappropriate expectation for such drugs (see Results Section Re-examining the sham experiment: Sham compliance introduces Hill-dependent bias in DEPmodels for further investigation into this issue).

3 Relationships between different synergy frameworks

3.1 Combination Index

Details of how MuSyC compares to combination index can be found in Box 2 and Supplemental Section "Percent Affect vs Percent Effect".

Table S1: **Translating CI.** Where possible, the original variable names from Chou et. al. have been translated to the equivalent variable names used in this manuscript, for ease of readability.

Variable name in[7]	Variable name in[8]	MuSyC variable name
$(f_i)_j$	$(f_a)_j$	$\overline{A_j}$
$(f_i)_{1,2,...n}$	$(f_a)_{1,2,...,n}$	A
$(f_v)_j$	$(f_u)_j$	$\overline{U_j}$
$(f_v)_{1,2,...n}$	$(f_u)_{1,2,...,n}$	U
m_j	m	h_j
$(I)_j$	$(D)_j$	d_j
$(I_{50})_j$	$(ED_{50})_j$	C_j

3.2 Effective dose model (Zimmer et. al.)

Zimmer et. al.[9] introduced the effective dose model as a parameterized extension of Bliss, and to our knowledge were the first to account the asymmetric potency synergy, which is also present in MuSyC. The effective dose model is constructed by fitting the dose response of each single drug to a two-parameter 1D Hill equation in which E_0 and E_{max} are fixed at 1 and 0, respectively

$$g(d_{1,eff}) = \frac{1}{1 + (\frac{d_{1,eff}}{C_1})^{h_1}} \tag{28}$$

$$g(d_{2,eff}) = \frac{1}{1 + (\frac{d_{2,eff}}{C_2})^{h_2}}$$

To model combination synergy, the authors propose transforming the doses d_i to "effective doses" via a system of equations coupling effective doses to one another via a Michaelis-Menten term in the denominator scaled by a synergy parameter a.

$$d_{1,eff} = \frac{d_1}{1 + a_{12}\left(\frac{1}{1+\left(\frac{d_{2,eff}}{C_2}\right)^{-1}}\right)}$$
$$d_{2,eff} = \frac{d_2}{1 + a_{21}\left(\frac{1}{1+\left(\frac{d_{1,eff}}{C_1}\right)^{-1}}\right)} \tag{29}$$

Table S2: **Translating the effective dose model.** Where possible, the original variable names from Zimmer et. al. have been translated to the equivalent variable names used in this manuscript, for ease of readability.

Original variable name	MuSyC variable name
n_1	h_1
$D0_1$	C_1
n_2	h_2
$D0_2$	C_2

The parameter a_{12} represents how drug 2 modifies the effective dose synergistically ($a_{12} < 0$) or antagonistically ($a_{12} > 0$) drug 1. Note that as $a_{12} \to -\left(\frac{1+d_{2,eff}/C_2}{d_{2,eff}/C_2}\right)$, $d_{1,eff} \to +\infty$, and as $a_{12} \to +\infty$, $d_{1,eff} \to 0$, which defines the bounds over which a_{12} is defined. The authors then fit the a parameters using a surface model based on MSP

$$E_d = g(d_{1,eff}) \cdot g(d_{2,eff}) \tag{30}$$

Thus the Effective Dose model reduces to the Bliss null model when $a_{12} = a_{21} = 0$. There are obvious similarities between Effective Dose model's a parameters and MuSyC's α values, as both reflect a potency transformation; however, the exact details are slightly different. For example, Zimmer assumes each drug has a Michaelis-Menten like effect on the potency of the other drugs (eq. (29)), whereas MuSyC can account for non-Michaelis-Menten effects (when $h \neq 1$). Furthermore, by using equation 28, Zimmer explicitly assumes the measured drug effect ranges from 100% to 0%, and fit the data with this constraint. Their model is unable to accurately describe combinations where the two drugs either have unequal maximum effects, or the combination has a greater effect than the drugs can achieve alone, features which are commonly observed [10] (Figure S7). In contrast, MuSyC is able to fit dose response surfaces with arbitrary effect ranges.

3.3 ZIP

Table S3: **Translating ZIP.** Where possible, the original variable names from Yadav et. al.[11] have been translated to the equivalent variable names used in this manuscript, for ease of readability. Note we found an erratum flipping x_2 and m_2 in the step from equation 13 to equation 14 in Yadav et. al. which is propagated through to equation 19. Our analysis uses the intended form.

Original variable name	Translated variable name
m_1	C_1
m_2	C_2
$m_{1\to2}$	$\mu_1 \cdot C_2$
$m_{2\to1}$	$\mu_2 \cdot C_1$
λ_1	h_1
λ_2	h_2
$\lambda_{1\to2}$	$\eta_1 \cdot h_2$
$\lambda_{2\to1}$	$\eta_2 \cdot h_1$
x_1	d_1
x_2	d_2
δ	δ

In contrast to the Effective Dose Model, ZIP, accounts for changes in both the Hill slope and the potency across the dose-response surface. In ZIP, these changes are integrated into a single number (δ), given by

$$\delta = \frac{1}{2}\left(\frac{\frac{1}{1+\frac{d_2}{C_2}^{h_2}} + \frac{d_1}{\mu_2 C_1}^{\eta_2 h_1}}{1+\frac{d_1}{\mu_2 C_1}^{\eta_2 h_1}} + \frac{\frac{1}{1+\frac{d_1}{C_1}^{h_1}} + \frac{d_2}{\mu 1 C_2}^{\eta 1 h_2}}{1+\frac{d_2}{\mu_1 C_2}^{\eta_1 h_2}}\right) - \left(\frac{1}{1+\frac{d_1}{C_1}^{-h_1}} + \frac{1}{1+\frac{d_2}{C_2}^{-h_2}} - \frac{1}{1+\frac{d_1}{C_1}^{-h_1}}\frac{1}{1+\frac{d_2}{C_2}^{-h_2}}\right) \tag{31}$$

ZIP is formulated for arbitrary E_0 and E_{max}; however, it assumes E_{max} is the same for both drugs, as well as the combination ($E_1 = E_2 = E_3$). To calculate δ, the ZIP method fixes the concentration of one drug, then fits a Hill-equation dose response for the other drug. However, for combinations with efficacy synergy or antagonism, slices of the dose-response surface can have non-Hill, and even non-monotonic shapes. In these cases, ZIP parameter fits may not be meaningful. Because MuSyC accounts explicitly for efficacy synergy, its surfaces are able to describe such complex drug combination surfaces where ZIP cannot.

Nevertheless, ZIP parameters μ and η are closely related to MuSyC parameters α and γ. In the absence of synergistic efficacy, slices of MuSyC dose-response surfaces are sigmoidal, though in general do not perfectly follow a Hill equation, and so the ZIP model is still not identical to MuSyC. However, at saturating concentrations of one or the other drug, MuSyC does reduce to the Hill equation. In these saturating cases, ZIP's δ can be related analytically to MuSyC's α and γ by

$$\lim_{d_2\to\infty} \delta(d_1, d_2) = \frac{1}{2}\left(\frac{1}{1+\left(\frac{d_1}{\alpha_{21}C_1}\right)^{\gamma_{21}h_1}}\right)$$

$$\lim_{d_1\to\infty} \delta(d_1, d_2) = \frac{1}{2}\left(\frac{1}{1+\left(\frac{d_2}{\alpha_{12}C_2}\right)^{\gamma_{12}h_2}}\right)$$

3.4 BRAID

BRAID[12] is an extension of the DEP to effects exceeding the weaker drug and consequently reduces to Loewe under particular conditions (Figure 2, main text). The authors propose three BRAID models with increasing complexity, with eBRAID capable of describing the most general dose-interaction surfaces. We focus our analysis on eBRAID, which assumes that each drug alone has a Hill-like response, and constructs an Hill-like equation for the combination

$$E = E_0 + \frac{E_3 - E_0}{1 + D^{-\delta\cdot\sqrt{h_1 h_2}}} \tag{32}$$

where

$$D = D1^{\frac{1}{\delta\cdot\sqrt{h_1 h_2}}} + D2^{\frac{1}{\delta\cdot\sqrt{h_1 h_2}}} + \kappa\sqrt{D1^{\frac{1}{\delta\cdot\sqrt{h_1 h_2}}} D2^{\frac{1}{\delta\cdot\sqrt{h_1 h_2}}}}$$

$$D1 = \frac{\frac{E_1-E_0}{E_3-E_0}\left(\frac{d_1}{C_1}\right)^{h_1}}{1+\left(1-\frac{E_1-E_0}{E_3-E_0}\right)\left(\frac{d_1}{C_1}\right)^{h_1}}$$

$$D2 = \frac{\frac{E_2-E_0}{E_3-E_0}\left(\frac{d_2}{C_2}\right)^{h_2}}{1+\left(1-\frac{E_2-E_0}{E_3-E_0}\right)\left(\frac{d_2}{C_2}\right)^{h_2}}$$

The BRAID equation (eq. (32)) uses a dose parameter, which combines the doses of both individual drugs, using a parameter κ and a parameter for the Hill slopes δ which acts as a multiplicative of the geometric mean hill slope $h = \sqrt{(h_1 h_2)}$. This formalism, like Loewe, is sham compliant under certain conditions, namely when $\kappa = 2^{\frac{-1}{\delta h}} - 2^{-h}$. By adjusting κ, BRAID is able to fit complex drug combination surfaces, including non-monotonic responses, unlike ZIP. Additionally, because BRAID fits the whole combination surface using a single parameter, it can be used to make unambiguous statements about whether the combination is synergistic or antagonistic. Nevertheless, BRAID does not account for differences in synergy due to efficacy, potency, and cooperativity, whereas we find many combinations that are synergistic with respect to one, but antagonistic with respect to the other (Figure 3C, main text). Though κ and δ are related to the potency and cooperativity respectively, their biochemical interpretation is not straightforward.

Table S4: **Translating the BRAID model.** Based on eBRAID model set of equations in the supplement. Note we corrected a typo in equation for $\tilde{D}_A$ where $ID_{M,B}$ is suppose to be $ID_{M,A}$

Original variable name	Translated variable name
D_A	d_1
D_B	d_2
$\tilde{D}_{AB}$	D
$\tilde{D}_A$	$D1$
$\tilde{D}_B$	$D2$
E_0	E_0
E_f	E_3
$E_{f,A}$	E_1
$E_{f,B}$	E_2
E_{AB}	E_d
n_a	h_1
n_b	h_2
$ID_{M,A}$	C_1
$ID_{M,B}$	C_2
δ	δ
κ	κ

3.5 General Pharmacodynamic Interaction Model

Table S5: **Translating the GPDI model.** Where possible, the original variable names from Wicha et. al. have been translated to the equivalent variable names used in this manuscript, for ease of readability.

Original variable name	MuSyC variable name
C_A	d_1
C_B	d_2
$EC50_A$	C_1
$EC50_B$	C_2
$EC50_{INT,AB}$	$C_{INT,12}$
$EC50_{INT,BA}$	$C_{INT,21}$
$H_{INT,AB}$	$h_{INT,12}$
$H_{INT,BA}$	$h_{INT,21}$
H_A	h_1
H_B	h_2
E_A	E_{drug1}
E_B	E_{drug2}
$Emax_A$	E_1
$Emax_B$	E_2
INT_{AB}	INT_{12}
INT_{BA}	INT_{21}

Wicha et. al. introduced the general pharmacodynamic interaction model (GPDI) for computing synergy[13]. Like MuSyC, GPDI quantifies synergy based on parameters describing the effect of one drug on the other's dose-response properties. Out of all other models of drug synergy, only MuSyC and GPDI can be used to distinguish synergy of potency or efficacy (while not discussed explicitly within the GPDI paper, it could equally be applied to cooperativity). For instance the effect of Drug 2 on Drug 1's potency is modeled as

$$C_1 \to C_1 \cdot \left(1 + INT_{12} \frac{d_2^{h_{INT,12}}}{C_{INT,12}^{h_{INT,12}} + d_2^{h_{INT,12}}}\right) \tag{33}$$

where $INT_{12} \in [-1, +\infty)$ represents the interaction strength of Drug 2 on Drug 1's potency, and $C_{INT,12}$ and $h_{INT,12}$ represent the EC_{50} and Hill slope of Drug 2's effect on Drug 1's potency, respectively. When $INT = 0$, the original parameter is unchanged, indicating no synergy or antagonism. The sign of INT reflects whether the interaction is synergistic or antagonistic. For instance, in (eq. (33)), $INT_{12} < 0$ would

indicate synergy as C_1 would decrease, while $INT_{21} > 0$ would indicate antagonism. The magnitude of synergy is given by the concentration-dependant (depending on d_2 in eq. (33)) change in C_1.

GPDI then computes the effect attributable to each drug, independently, using a three-parameter Hill equation (data is transformed so $E_0 = 0$). For example, given two drugs interacting via potency, the GPDI model says (see Equations 1 and 2 from[13])

$$E_{drug1} = E_1 \frac{d_1^{h_1}}{\left(C_1 \cdot \left(1 + INT_{12} \frac{d_2^{h_{INT,12}}}{C_{INT,12}^{h_{INT,12}} + d_2^{h_{INT,12}}}\right)\right)^{h_1} + d_1^{h1}} \tag{34}$$

$$E_{drug2} = E_2 \frac{d_2^{h_2}}{\left(C_2 \cdot \left(1 + INT_{21} \frac{d_1^{h_{INT,21}}}{C_{INT,21}^{h_{INT,21}} + d_1^{h_{INT,21}}}\right)\right)^{h_2} + d_2^{h2}} \tag{35}$$

These effects can then be inserted into any model of drug effect additivity, such as simple additivity ($E = E_{drug1} + E_{drug2}$), Bliss Independence ($E = E_{drug1} + E_{drug2} - E_{drug1} \cdot E_{drug2}$), Loewe Additivity ($1 = \frac{d_1}{E_{drug1}^{-1}(E)} + \frac{d_2}{E_{drug2}^{-1}(E)}$), or any other.

This approach is similar to the effective dose model (29), but is not limited to describing synergistic potency, nor is it limited to the Bliss Independence effect additivity model. Further, unlike both MuSyC and the Effective Dose model, in GPDI, $C_{INT,21}$ and $h_{INT,21}$ may be different than C_1 and h_1. This means GPDI allows the effect a drug on other drugs have different dynamics than the effect it has on the cells. However, GPDI describes synergistic potency as a multiplicative factor on a drug's EC_50, whereas MuSyC and the effective dose model describe it as a multiplicative factor on a drug's dose. Thus, unlike in MuSyC and the Effective Dose model, in GPDI the "boost" Drug 1 may receive from other drugs does not feedback to the effect Drug 1 has on the other drugs' parameters, and vice versa. In other words, while Drug 2 may affect Drug 1's potency (C_1) via equation (33), this does not affect Drug 1's interaction on Drug 2 ($C_{INT,21}$).

Most significantly, unlike MuSyC, GPDI requires a researcher to choose a drug additivity model, such as Bliss or Loewe, whereas MuSyC is a single framework generalizing both models. As noted in[13], choosing Bliss versus Loewe can lead to different conclusions regarding synergy and antagonism, even when either model describes the data similarly well.

3.6 Highest Single Agent

Highest Single Agent (HSA)[6] is a parsimonious model that defines synergy as the net difference between the combination response and the stronger single-drug response

$$HSA = min(E_d(d_1, 0), E_d(0, d_2)) - E_d(d_1, d_2) \tag{36}$$

This form assumes that drug decreases E, though it can also be defined for drugs that increase E. At high concentrations of d_1 and d_2, equation (36) becomes proportional to our definition of efficacy synergy (β) as shown in equation 37 and Figure 2 (main text). Nevertheless, at intermediate doses, HSA will conflate synergy of potency, efficacy, and cooperativity (Figure 3C, main text) highlighting the importance of considering the whole dose-response surface when calculating synergy.

$$\lim_{d_1, d_2 \to \infty} HSA(d_1, d_2) = \beta \cdot (E_0 - min(E_1, E_2)) \tag{37}$$

3.7 2D Hill PDE

$$E_d = \left(\frac{E_1(\frac{d_1}{C_1}) + E_2(\frac{d_2}{C_2})}{(\frac{d_1}{C_1}) + (\frac{d_2}{C_2})}\right)\left(\frac{1}{1 + ((\frac{d_1}{C_1}) + (\frac{d_2}{C_2}))^{-\left(\frac{h_1(\frac{d_1}{C_1}) + h_2(\frac{d_2}{C_2})}{(\frac{d_1}{C_1}) + (\frac{d_2}{C_2})}\right)}}\right) \tag{38}$$

The most recent framework is one by Schindler[14] which interpolates a null dose-response surface from the single dose-response curves alone without any fit parameters. This was done by using PDE Hill equations and then imposing boundary conditions as well as sham compliance. It is therefore an extension of Loewe to effects greater than the least efficacious drug (Figure 2, Table 2, main text). The boundary conditions enforce the null model's maximal effect of the combination (E_3) is equal to the mean of E_1 and E_2. This results in the non-intuitive scenarios such as if the maximal effect of one compound is 0.25 and the other is 0.75, then the predicted maximal effect of the combination is 0.5 which is much less than achievable with a single drug.

Table S6: **Translating the Schindler et. al. model.** Based on equations 7 and 8 in the main text.

Parameter	Translated
m_a	d_1/C_1
m_b	d_2/C_2
a_{max}	E_1
b_{max}	E_2
α	h_1
β	h_2

4 Percent Affect vs Percent Effect

4.1 MSP

It is generally known that Bliss, and more generally the MSP methods, are only applicable to percentage data. Indeed, percent transformations are commonly applied to data in order to apply Bliss. In Bliss' original study, drug effect was quantified as the percentage of eggs killed (the probability that each egg would die at a given toxin dose), but in all cases the measurement was a discrete event (death of an insect egg). Therefore, the metric of drug effect was the a percentage of affected eggs. However, to apply Bliss to more general measures of drug effect for which discrete counts cannot be obtained, it has been ubiquitous practice to normalize the drug effect as a percent relative to control (e.g., percent viability in cancer research). Nevertheless, such normalization does not, in general, transform measures of efficacy into measures of percent affect.

Suppose, as an example, analyzing a drug treatment which actually caused the cells to grow slightly faster than control. By normalizing the drug effect to control, the percent viability is greater than 100% which cannot mean that >100% of the cells were affected.

Alternatively, consider the case when a cytostatic drug causes all treated cells to halt both proliferation and death. If the control population doubled twice over 72 hours, the percent viability would be $\frac{1}{2^3} = 16.25\%$. If the measure of percent viability was taken instead at 96 hours, the percent viability would be $\frac{1}{2^4} = 6.25\%$. At both time points the percent of affected cells was the same (100%); however, the percent of drug effect changes due to normalization.

4.2 Combination Index

Most DEP frameworks can work with any effect metric or effect range. However, CI does expect data to measure percent affect. CI is defined as[8]

$$CI(d_1, d_2) = \frac{d_1}{f_1^{-1}(E)} + \frac{d_2}{f_2^{-1}(E)}$$

where d_1 and d_2 are the doses of drugs 1 and 2, E is the effect (experimentally measured) achieved by the combination of those doses, and $f_1^{-1}(E)$ and $f_2^{-1}(E)$ give the dose of Drug 1 or Drug 2 that, when treated as a single-agent, achieve effect E. In CI, $E = f(d)$ is determined by the mass-action derived median-effect equation (Eq. 8, main text). As in Box 1, this leads to the two-parameter Hill equation (Eq. 9, main text) $U = \frac{C^h}{C^h + d^h}$. CI asserts $E = f(d) \equiv U$, the unaffected (or uninhibited) fraction of the target. In the context of molecular inhibition, this leads to expected behavior. For instance, $f(0) = 1$ (in the absence of drug, 100% of targets are uninhibited), and as $d \to \infty$, $f(d) \to 0$ (at saturating drug, all targets are inhibited).

But in drug assays, the measured effect is often not the fraction of (un)inhibited target molecules. Instead, common metrics include population-level readouts such as percent viability, colony formation, or proliferation rate. These data often do not approach 0 as $d \to \infty$, but may instead approach some non-zero maximum effect (Figure 6A, main text). As shown in Figure 6A (main text), when a drug's maximum effect is non-zero, the CI method fits data poorly, which can introduce significant errors in $f^{-1}(E)$. These errors can be observed in systematic, dose-dependent biases in CI synergy (Figure 6B, C, main text).

Unlike CI, MuSyC asserts $E = f(d) \equiv U \cdot E_0 + A \cdot E_1$, such that $f(0) = E_0$ and $\lim_{d \to \infty} = E_1$. MuSyC can thus be used to describe data with arbitrary finite efficacy limits. It is possible to redefine CI to use the same $f(d)$, the four-parameter Hill equation, as MuSyC. Indeed, this is exactly what we do to quantify Loewe synergy throughout this paper, due to the similarities between the definition of Loewe and CI. Loewe, however, has its own limitations discussed throughout this work. As a well-known example, $f_j^{-1}(E)$ becomes undefined for effects E that exceed the maximum efficacy of Drug j. Because of this, it is impossible to define Loewe synergy for effects exceeding the maximum effect of the weakest drug, because for that drug, $f^{-1}(E)$ is undefined.

5 Sham Compliance of Synergy Frameworks

To verify a new synergy model's consistency, it is traditionally tested with the "sham" combination thought experiment. Briefly, the thought experiment proposes a single drug is divided into two vials labeled drugs "A" and "B", before the vials are given to an unsuspecting researcher —who does not know they are the same drug —to perform combination synergy measurements[15]. Any synergy metric finding either synergy or antagonism for this "sham combination" fails the thought experiment, because a drug combined with itself should be additive. HSA, as well as Bliss and other MSP frameworks (Figure 2, main text) famously fail the sham experiment[6]. In contrast, Loewe additivity and other DEP frameworks are sham complaint.

In reviewing the literature, we identified a two errors beyond the Hill slope bias (Figure 5, main text) pertaining to the sham experiment that merit addressing.
1) Chou, one of the creators of the Combination Index (CI), has strongly argued that satisfaction of the sham experiment is critical. However, we identified an error in the derivation of the CI from its underlying model (see Box 2), so that while the CI equation is sham-compliant, the biochemical model proposed by Chou and Talalay is not.
2) ZIP was proposed as a framework that unifies Loewe Additivity (DEP) and Bliss Independence (MSP), much like MuSyC. In support of this, they prove ZIP is sham-compliant; however, we identified an error in their proof. To show this, we generate *in silico* sham response data and show that ZIP fails the sham experiment. For this reason, we place ZIP as an MSP method in Figure 2C (main text), and not DEP. However, overall we contend that satisfying the sham experiment should not be a sought-after standard in drug combinations, so we do not consider this a shortcoming of ZIP.

5.1 Sham Compliance of ZIP

ZIP was reported to satisfy the sham experiment[11] based on an argument that if both drugs are the same, then $m_1 = m_2 = m_{1\leftarrow 2} = m_{2\leftarrow 1}$. However, this statement is false for sham experiments, because once some of the drug is added, $m_{1\leftarrow 2}$ is shifted. To demonstrate this, consider their model (equation 13 from[11], without asserting $m_{1\leftarrow 2} = m_1$)

$$y_{1\leftarrow 2} = \frac{y_2 + \left(\frac{x_1}{m_{1\leftarrow 2}}\right)^{h_1}}{1 + \left(\frac{x_1}{m_{1\leftarrow 2}}\right)^{h_1}}$$

$m_{1\leftarrow 2}$ is the amount x_1 of drug 1 that is needed to achieve an effect halfway between y_2 and 1 (the asymptotic value at infinite drug), where y_2 is the effect achieved by adding an amount x_2 of drug 2. Specifically, when $x_1 = m_{1\leftarrow 2}$, $y_{1\leftarrow 2} = \frac{1+y_2}{2}$.

Sham experiments satisfy Equation 18 (main text), which combined with the above gives

$$y_{1\leftarrow 2} = \frac{\left(\frac{m_{1\leftarrow 2}+x_2}{m_1}\right)^{h_1}}{1 + \left(\frac{m_{1\leftarrow 2}+x_2}{m_1}\right)^{h_1}} = \frac{1+y_2}{2}$$

Further we know

$$y_2 = \frac{\left(\frac{x_2}{m_1}\right)^{h_1}}{1 + \left(\frac{x_2}{m_1}\right)^{h_1}}$$

This system can be solved to find

$$m_{1\leftarrow 2} = m_1 \left[1 + 2\left(\frac{x_2}{m_1}\right)^{h_1}\right]^{\frac{1}{h_1}} - x_2$$

demonstrating that $m_{1\leftarrow 2} \neq m_1$ for the sham experiment in the ZIP model, contradicting their proof that ZIP is sham-compliant.

To verify ZIP identifies synergy or antagonism for sham combinations, we generated a synthetic sham dose response surface. Sham experiments can be generated exactly for any drug with a pre-defined dose-response by asserting the condition in equation 18 (main text). We constructed a synthetic dataset describing a sham dose response surface for a drug with $h = 2$, sampled at 2.5 orders of magnitude above and below the EC50. One drug was sampled at 7 concentrations, the other at 12, defining a 7 x 12 sham dose response matrix. We used the synergyfinder[5] R package to calculate synergy by both Loewe and ZIP (Figure S11), and found Loewe reported close to 0 synergy, as expected for a sham combination, but confirms ZIP detects synergy and antagonism at several concentrations.

6 MuSyC statistically distinguishes efficacious and non-efficacious drug combinations in clinical trials based on *in vitro* combination screens.

From the datasets in Table 3 (main text), we identified 126 combinations with clinical trial information annotated in the Drug Combination Database (DCDB)[16]. These combinations were tested in a total of 2,996 *in vitro* disease models (Figure S1A,B). Of these clinical combinations, 18% were annotated as "Non-efficacious" in DCDB, compared to 75% annotated "Efficacious" (Figure S1C). We found clinically efficacious combinations have higher synergistic efficacy than clinically non-efficacious combinations, based on data from the *in vitro* screens (Figure S9A left panel, one-sided t-test p-val=0.002). However, we found no significant difference in synergistic potency between the two sets (Figure S9A middle, p-val>0.05) pointing to the importance of distinguishing different types of synergy. Loewe (Figure S9A), Bliss, and CI (Figure S10D) were unable to distinguish clinically efficacious combinations. Only HSA, which is related to synergistic efficacy (Figure 2C, main text), is statistically higher in efficacious combinations (Figure S10).

As an example, the combination of gemcitabine and docetaxel for the treatment of prostate cancer was tested in two Phase II clinical trials, where it did not improve overall survival over docetaxel alone[17,18]. Loewe rates this combination as synergistic (Figure S9B), while MuSyC finds this combination to be antagonistically efficacious (Figure S9B) and antagonistically potent in the prostate cell line PC3. Loewe's incorrect designation as synergistic stems in part from the Hill-slope bias in Loewe (Figure 5C,D, main text), as $\sqrt{h_1 \cdot h_2} < 1$. This analysis illustrates the potential for *in vitro* drug combination screens to impact the clinic if analyzed via MuSyC.

Despite over 100 years of work on drug synergy frameworks, few successful clinical combinations have been discovered from *in vitro* screens. This is commonly attributed to the lack of clinical relevance of *in vitro* models. However, significantly less discussion has centered around how the quantification of *in vitro* screens impacts clinical relevance. Using MuSyC, we here distinguished clinically efficacious and non-efficacious combinations based on *in vitro* screens, whereas prior art in the field cannot. However, there are important caveats in this analysis that merit commentary. The definition of "non-efficacious" is idiosyncratic to each individual trial, and therefore not all combinations were judged the same way. For instance, different trials had different patient-selection guidelines, or control arms for determining improvement. Additionally, because most clinical trials are focused on efficacy gains as compared to dose reduction, it is unsurprising synergy of efficacy, but not synergy of potency, distinguished clinical successes. MuSyC thus lays the foundation for designing clinical trials that assess the translation of specific types of synergistic interactions (e.g., synergistic potency, as opposed to efficacy).

7 Proof of boundary behavior of the 2D Hill equation

When $d_1 \to \infty$, then the 4 state reduces to 2 states transition model between A1 and A12.

$$A_1 \underset{r_{-2}^{\gamma_{12}}}{\overset{r_2^{\gamma_{12}} \cdot (\alpha_{12} d_2)^{\gamma_{12} h_2}}{\rightleftharpoons}} A_{1,2} \tag{39}$$

At equilibrium

$$\frac{A_{1,2}}{A_1} = \frac{r_2^{\gamma_{12}} (\alpha_{12} d_2)^{\gamma_{12} h_2}}{r_{-2}^{\gamma_{12}}} \tag{40}$$

When $A_1 = A_{1,2}$ this is the EC50 for drug 2 given saturating concentrations of drug 1 (C_2'). This dose can be found by solving the above.

$$C_2' = \frac{C_2}{\alpha_{12}} = \frac{1}{\alpha_{12}} \left(\frac{r_2}{r_{-2}} \right)^{1/h_2} \tag{41}$$

The boundary then reduces to a Hill equation of the form

$$E = E_3 + \frac{E_1 - E_3}{1 + \frac{\alpha_{12} d_2}{C_2}^{\gamma_{12} h_2}} \tag{42}$$

following the derivation in box 1. By this we can see when $d_1 \to \infty$, the system reduces to a new HIll equation with potency ($\frac{C2}{\alpha_{12}}$), Hill slope ($\gamma_{12} h_2$), and efficacy ($E_1 - E_3$). It is important to note, unlike ZIP, intermediate doses are not necessarily Hill curves when $\beta \neq 1$. Indeed, it is possible to have non-monotonic curves allowing MuSyC to capture complex interactions between drugs.

8 MuSyC Web Application

8.1 Data format

The MuSyC web application (https://musyc.lolab.xyz) allows users to run the MuSyC algorithm, obtain dose-response surface parameters, and visualize the dose-response surface using a web browser.

Users' data is uploaded in comma-separated value (CSV) format, using the Unicode UTF-8 encoding (the default across most software). The required columns and their contents are shown in Table S7. Combinations are grouped based on drug1, drug2, sample, and batch columns. Thus, multiple combinations can be uploaded in the same file and will be processed separately. The drug1.units and drug2.units are arbitrary, but must be consistent within each combination.

Table S7: **CSV columns for MuSyC web application**.

Column Name	Data type	Description
expt.date	yyyy-mm-dd	Experiment date
drug1.conc	Float	Drug 1 concentration
drug2.conc	Float	Drug 2 concentration
effect	Float	Effect value (e.g., % viability, DIP rate)
sample	String	Sample name (e.g., name of cell line)
drug1	String	Drug 1 name
drug2	String	Drug 2 name
drug1.units	String	Drug 1 units (e.g., nM)
drug2.units	String	Drug 2 units (e.g., nM)
effect.95ci	Float (optional)	95% confidence interval for effect value
batch	String (optional)	Batch name

8.2 Usage

8.2.1 Create an account

Open https://musyc.lolab.xyz in a web browser, and click "Create an account". Enter your email address and a choice of password. Your email address will be verified by sending an email with a clickable link. Click on this link, and you will then be able to log in to the site with your credentials.

8.2.2 Create a dataset

After logging in, click the "Create dataset" button. Enter a name for the dataset, and use the "browse" button to select a CSV file from your computer. You can also select the orientation of the dose response surface (whether Emax >E0, or otherwise), the name of the effect metric (e.g., percent effect, DIP rate), apply any constraints to the E0 and Emax values (either unconstrained, fixed values, or range bounds). Click the "Create dataset" button on this page, and the upload will begin.

8.2.3 View results

The dataset is first uploaded to the server and validated. Each combination experiment is then sent to a queue for processing. The processing step runs the MuSyC algorithm to fit the dose-response surface, and returns the relevant fitting parameters. For small datasets, this process typically only takes a few minutes, but this will vary depending on dataset size and server demand.

When the upload is complete, the web browser will redirect to a page showing the dataset name and a list of the combination experiments in a table. A progress bar indicates whether there are still fitting tasks queued, in progress, or completed. At the bottom of the page, there is a link to download the dataset's fitting parameters as a CSV file.

Each drug combination in the table shows the fitting algorithm's status (e.g., queued, started, success, failed). For successful tasks, clicking on the word "SUCCESS" will show the parameters for that combination, along with an interactive dose-response surface plot, which can be zoomed, panned, rotated etc. in the web browser. For tasks marked as "FAILED", clicking on that word will show more details about the error (e.g., if there was a data validation issue that the user should correct). There is also a link at the bottom of the task result page to download that single combination's parameters as a CSV file.

9 Interactive MuSyC Jupyter Notebook

We created an interactive Jupyter Notebook that can be used to explore how MuSyC's 2D Hill-equation parameters relate to the dose-response surface, as well as how they relate to Bliss and Loewe synergy.

This notebook, the backend code, and a conda environment file are included in File S1. The notebook may be run locally, but we also provide a version online at https://mybinder.org/v2/gh/djwooten/natcomms-musyc2021/HEAD?filepath=demo.ipynb. To run the code locally, extract the contents of File S1 to a directory. We recommend using Anaconda to manage installation of Python dependencies. On MacOS or Linux, from a command line terminal in the directory of the contents of File S1, the following commands will install the necessary Python dependencies and run the notebook:

```
conda env create -f environment.yml
conda activate musyc_demo
jupyter-notebook demo.ipynb
```

From Windows, we recommend using the Anaconda GUI application to set up the conda environment.

As noted in Table 2, Bliss and Loewe are not concentration independent synergy models, and thus synergy can be calculated individually at each concentration. Doing so leads to a Bliss or Loewe "synergy landscape". Using this interactive notebook, we explored how distinct types of synergy (e.g., potency, efficacy) may be reflected in the Bliss or Loewe synergy landscapes. Figures S12-S16 show what happens to the Bliss synergy landscape as specific MuSyC synergy parameters are adjusted. Specifically, synergistic efficacy is reflected in the Bliss synergy landscape at high concentrations of both drugs, while synergistic potency and synergistic cooperativity are reflected near the EC50 of one drug, and high dose of the other. Similar observations can be found in the notebook for Loewe synergy. Nevertheless, the simultaneous presence of multiple synergy types can lead to difficult to disentangle Bliss or Loewe landscapes, which can be compounded by the biases discussed throughout the text. See the examples in File S1 for more details. We note that some real combinations may not be well fit by MuSyC's 2D Hill equation, and in these cases, the Bliss or Loewe synergy landscapes may be able to provide specific insights that MuSyC is unable to capture. Nevertheless, a key advantage of MuSyC is that it is able to describe most real-world complex synergy landscapes in a small number of easy to interpret synergy parameters.

References

[1] Jennifer O'Neil, Yair Benita, Igor Feldman, Melissa Chenard, Brian Roberts, Yaping Liu, Jing Li, Astrid Kral, Serguei Lejnine, Andrey Loboda, William Arthur, Razvan Cristescu, Brian B. Haines, Christopher Winter, Theresa Zhang, Andrew Bloecher, and Stuart D. Shumway. An Unbiased Oncology Compound Screen to Identify Novel Combination Strategies. *Molecular Cancer Therapeutics*, 15(6):1155–1162, 6 2016.

[2] Susan L. Holbeck, Richard Camalier, James A. Crowell, Jeevan Prasaad Govindharajulu, Melinda Hollingshead, Lawrence W. Anderson, Eric Polley, Larry Rubinstein, Apurva Srivastava, Deborah Wilsker, Jerry M. Collins, and James H. Doroshow. The National Cancer Institute ALMANAC: A Comprehensive Screening Resource for the Detection of Anticancer Drug Pairs with Enhanced Therapeutic Activity. *Cancer Research*, 77(13):3564–3576, jul 2017.

[3] Bryan T. Mott, Richard T. Eastman, Rajarshi Guha, Katy S. Sherlach, Amila Siriwardana, Paul Shinn, Crystal McKnight, Sam Michael, Norinne Lacerda-Queiroz, Paresma R. Patel, Pwint Khine, Hongmao Sun, Monica Kasbekar, Nima Aghdam, Shaun D. Fontaine, Dongbo Liu, Tim Mierzwa, Lesley A. Mathews-Griner, Marc Ferrer, Adam R. Renslo, James Inglese, Jing Yuan, Paul D. Roepe, Xin-zhuan Su, and Craig J. Thomas. High-throughput matrix screening identifies synergistic and antagonistic antimalarial drug combinations. *Scientific Reports*, 5(1):13891, 11 2015.

[4] Murat Cokol, Hon Nian Chua, Murat Tasan, Beste Mutlu, Zohar B Weinstein, Yo Suzuki, Mehmet E Nergiz, Michael Costanzo, Anastasia Baryshnikova, Guri Giaever, Corey Nislow, Chad L Myers, Brenda J Andrews, Charles Boone, and Frederick P Roth. Systematic exploration of synergistic drug pairs. *Molecular systems biology*, 7(1):544, 11 2011.

[5] Liye He, Evgeny Kulesskiy, Jani Saarela, Laura Turunen, Krister Wennerberg, Tero Aittokallio, and Jing Tang. Methods for High-throughput Drug Combination Screening and Synergy Scoring. *Methods in molecular biology (Clifton, N.J.)*, 1711:351–398, 2018.

[6] Julie Foucquier and Mickael Guedj. Analysis of drug combinations: current methodological landscape. *Pharmacology research & perspectives*, 3(3):e00149, 6 2015.

[7] T C Chou and P Talalay. Generalized equations for the analysis of inhibitions of Michaelis-Menten and higher-order kinetic systems with two or more mutually exclusive and nonexclusive inhibitors. *European journal of biochemistry*, 115(1):207–16, 3 1981.

[8] T C Chou and P Talalay. Quantitative analysis of dose-effect relationships: the combined effects of multiple drugs or enzyme inhibitors. *Advances in enzyme regulation*, 22:27–55, 1984.

[9] Anat Zimmer, Itay Katzir, Erez Dekel, Avraham E Mayo, and Uri Alon. Prediction of multidimensional drug dose responses based on measurements of drug pairs. *Proceedings of the National Academy of Sciences of the United States of America*, 113(37):10442–7, 9 2016.

[10] Mohammad Fallahi-Sichani, Saman Honarnejad, Laura M Heiser, Joe W Gray, and Peter K Sorger. Metrics other than potency reveal systematic variation in responses to cancer drugs. *Nature Chemical Biology*, 9(11):708–714, 9 2013.

[11] Bhagwan Yadav, Krister Wennerberg, Tero Aittokallio, and Jing Tang. Searching for Drug Synergy in Complex Dose–Response Landscapes Using an Interaction Potency Model. *Computational and Structural Biotechnology Journal*, 13:504–513, 1 2015.

[12] Nathaniel R. Twarog, Elizabeth Stewart, Courtney Vowell Hammill, and Anang A. Shelat. BRAID: A Unifying Paradigm for the Analysis of Combined Drug Action. *Scientific Reports*, 6(1):25523, 7 2016.

[13] Sebastian G. Wicha, Chunli Chen, Oskar Clewe, and Ulrika S.H. Simonsson. A general pharmacodynamic interaction model identifies perpetrators and victims in drug interactions. *Nature Communications*, 2017.

[14] Michael Schindler. Theory of synergistic effects: Hill-type response surfaces as 'null-interaction' models for mixtures. *Theoretical Biology and Medical Modelling*, 14(1):15, 12 2017.

[15] Ting-Chao Chou, Paul Talalay, M. Bowie, T.-C. Chou, J.M. Budinger, K.A. Watanabe, J.J. Fox, and F.S. Philips. Analysis of combined drug effects: a new look at a very old problem. *Trends in Pharmacological Sciences*, 4:450–454, 1 1983.

[16] Y. Liu, Q. Wei, G. Yu, W. Gai, Y. Li, and X. Chen. DCDB 2.0: a major update of the drug combination database. *Database*, 2014(0):bau124–bau124, dec 2014.

[17] Trine Zeeberg Buch-Hansen, Lise Bentzen, Steinbjoern Hansen, Morten Hoeyer, Niels Viggo Jensen, Charlotte Saxe, and Lisa Sengeloev. Phase I/II study on docetaxel, gemcitabine and prednisone in castrate refractory metastatic prostate cancer. *Cancer Chemotherapy and Pharmacology*, 66(2):295–301, jul 2010.

[18] Jorge A. Garcia, Thomas E. Hutson, Dale Shepard, Paul Elson, and Robert Dreicer. Gemcitabine and docetaxel in metastatic, castrate-resistant prostate cancer. *Cancer*, 117(4):752–757, feb 2011.
