# Supplementary material for: MuSyC is a consensus framework that unifies multi-drug synergy metrics for combinatorial drug discovery
Source: Nat Commun. 2021 Jul 29;12:4607. doi: 10.1038/s41467-021-24789-z (PMC8322415; doi:10.1038/s41467-021-24789-z)
Supplement: Supplementary file 2 — Description of Additional Supplementary Files [file 41467_2021_24789_MOESM2_ESM.pdf]

Description of Additional Supplementary Files

File Name: Supplementary Code 1
Description: A zip archive including coe and instructions needed to run an interactive Jupyter Notebook demonstrating the MuSyC synergy framework, its parameters, and its relationship to the Bliss and Loewe synergy frameworks.
