# Supplementary figures and images for: MuSyC is a consensus framework that unifies multi-drug synergy metrics for combinatorial drug discovery
Source: Nat Commun. 2021 Jul 29;12:4607. doi: 10.1038/s41467-021-24789-z (PMC8322415; doi:10.1038/s41467-021-24789-z)

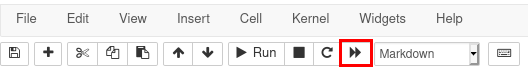

Supplement: Supplementary file 3 — Supplementary Code 1 [file 41467_2021_24789_MOESM3_ESM.zip › run_instructions.png]

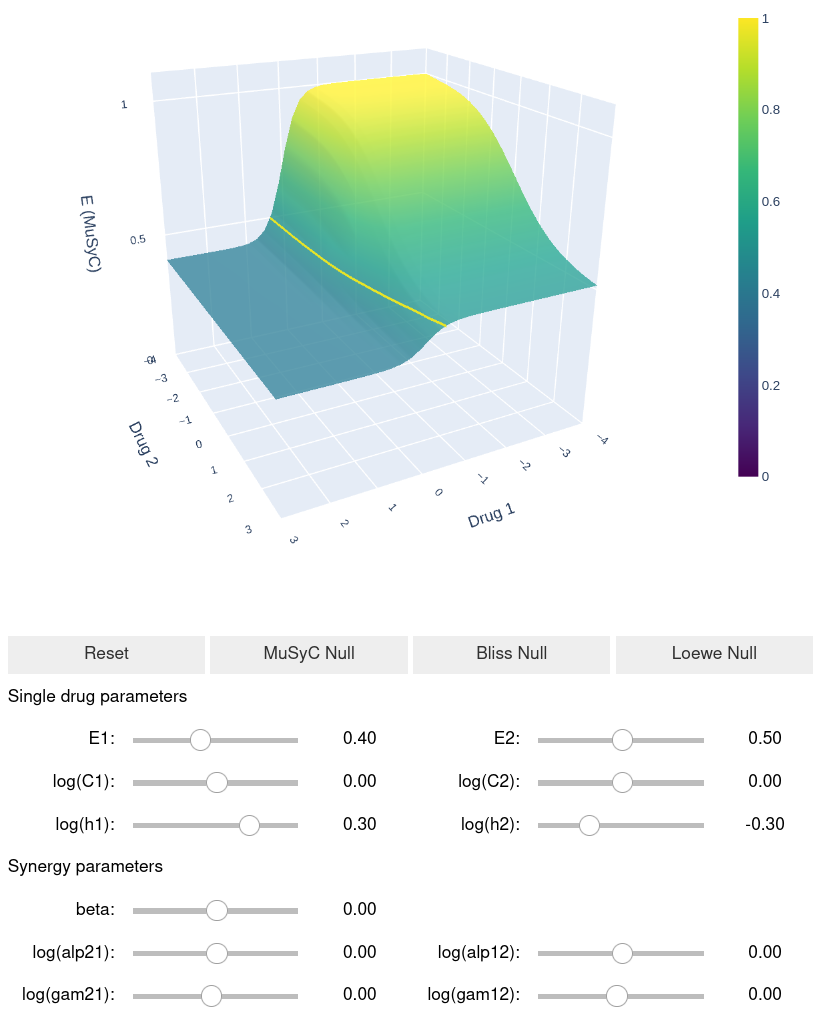

Supplement: Supplementary file 3 — Supplementary Code 1 [file 41467_2021_24789_MOESM3_ESM.zip › screenshot.png]
