# Supplementary material for: MuSyC is a consensus framework that unifies multi-drug synergy metrics for combinatorial drug discovery
Source: Nat Commun. 2021 Jul 29;12:4607. doi: 10.1038/s41467-021-24789-z (PMC8322415; doi:10.1038/s41467-021-24789-z)
Supplement: Supplementary file 4 — Reporting Summary [file 41467_2021_24789_MOESM4_ESM.pdf]

nature research

Corresponding author(s): Carlos F. Lopez

Last updated by author(s): June 7, 2021

Reporting Summary

Nature Research wishes to improve the reproducibility of the work that we publish. This form provides structure for consistency and transparency in reporting. For further information on Nature Research policies, see our Editorial Policies and the Editorial Policy Checklist.

Statistics

For all statistical analyses, confirm that the following items are present in the figure legend, table legend, main text, or Methods section.

n/a	Confirmed	
☐	☒	The exact sample size (*n*) for each experimental group/condition, given as a discrete number and unit of measurement
☐	☒	A statement on whether measurements were taken from distinct samples or whether the same sample was measured repeatedly
☐	☒	The statistical test(s) used AND whether they are one- or two-sided<br>*Only common tests should be described solely by name; describe more complex techniques in the Methods section.*
☒	☐	A description of all covariates tested
☐	☒	A description of any assumptions or corrections, such as tests of normality and adjustment for multiple comparisons
☐	☒	A full description of the statistical parameters including central tendency (e.g. means) or other basic estimates (e.g. regression coefficient) AND variation (e.g. standard deviation) or associated estimates of uncertainty (e.g. confidence intervals)
☐	☒	For null hypothesis testing, the test statistic (e.g. *F*, *t*, *r*) with confidence intervals, effect sizes, degrees of freedom and *P* value noted<br>*Give P values as exact values whenever suitable.*
☒	☐	For Bayesian analysis, information on the choice of priors and Markov chain Monte Carlo settings
☒	☐	For hierarchical and complex designs, identification of the appropriate level for tests and full reporting of outcomes
☒	☐	Estimates of effect sizes (e.g. Cohen's *d*, Pearson's *r*), indicating how they were calculated

Our web collection on statistics for biologists contains articles on many of the points above.

Software and code

Policy information about availability of computer code

Data collection	No software was used for data collection.
Data analysis	All code required for recreating manuscript analyses from the MuSyC fits are available for review in the repository (https://bitbucket.org/meyerct1/musyc_theory/). A web application to calculate MuSyC parameters is available at (https://musyc.lolab.xyz). Software used in analyses was:<br>- Python v2.7<br>And python libraries:<br>- numpy v1.14.3<br>- scipy v1.1.0<br>- pandas v0.23.0<br>- matplotlib v2.2.2<br>- uncertainties v3.0.2<br>- fuzzywuzzy v0.17.0

For manuscripts utilizing custom algorithms or software that are central to the research but not yet described in published literature, software must be made available to editors and reviewers. We strongly encourage code deposition in a community repository (e.g. GitHub). See the Nature Research guidelines for submitting code & software for further information.

Data

Policy information about availability of data

All manuscripts must include a data availability statement. This statement should provide the following information, where applicable:

- Accession codes, unique identifiers, or web links for publicly available datasets
- A list of figures that have associated raw data
- A description of any restrictions on data availability

The datasets analyzed in this study were obtained from publicly available sources with DOIs Mott (10.1038/srep13891, Figures 3, 6, S4, S6, S9), O'Neil (10.1158/1535-7163.MCT-15-0843, Figures 3-4, S4-S9), Holbeck (10.1158/0008-5472.CAN-17-0489, Figures 7, S6, S9), Tan (10.1038/nbt.2391, Figure S6), and Cokol (10.1038/msb.2011.71, Figures S6, S9). Clinical trial data was collected from the Drug Combination Database (DCDB) (10.1093/database/bau124, http://public.synergylab.cn/dcdb/, Figures S9-S10). The synergy datasets generated in this study are available in a repository at https://bitbucket.org/meyerct1/musyc_theory/.

Field-specific reporting

Please select the one below that is the best fit for your research. If you are not sure, read the appropriate sections before making your selection.

☒ Life sciences ☐ Behavioural & social sciences ☐ Ecological, evolutionary & environmental sciences

For a reference copy of the document with all sections, see nature.com/documents/nr-reporting-summary-flat.pdf

Life sciences study design

All studies must disclose on these points even when the disclosure is negative.

Sample size	Data were collected from publicly available sources, and include over 340,000 drug combinations with many different metrics of drug effect, in many different cell lines and disease contexts. The smallest study (Tan) included 116 combinations. Collectively this provides an enormous number of conditions to observe biases in synergy.
Data exclusions	Combinations were excluded if parameter estimation did not converge, which was a predetermined criteria. Additionally, combinations whose fits had an R^2 value <=0.7 were excluded, which was chosen based on visual inspection of fit qualities. For analyses reporting systematic biases, combinations whose best fit EC50 was not between the minimum and maximum tested dose were excluded, because such combinations lacked data to tightly constrain parameters. This exclusion was established after fitting hte data. Some synergy methods, such as Combination Index, become undefined for datapoints with E < 0 or E > 1, and thus those points were excluded from analysis in those cases. This exclusion was predetermined. In the analysis comparing drug synergy with clinical trial efficacy, all drugs whose name had <85% fuzzy match (as described in methods) with a drug used in clinical trials were excluded (this value was predetermined).
Replication	Experimental replication was not possible as all experimental data come from previously published sources. Each experiment was analyzed using synthetic replicates generated using Monte Carlo resampling to obtain bootstrapped 95% confidence interavals for each MuSyC parameters. A synergy parameter was deemed synergistic (or antagonistic) only if its entire 95% confidence interval indicated synergy (or antagonism).
Randomization	For observing large-scale trends, all data that were not excluded were analyzed equally with no sub-sampling, and thus no randomization was required. Individual example combinations that are highlighted were selected manually to have: (1) a high R^2 fit value indicating that the MuSyC model fits the data well, (2) EC50 values for each drug near the middle of the tested dose range so the full dose-response surface was clear, and (3) drug-response parameters leading to large bias to illustrate the intended conclusions.
Blinding	N/A: Blinding was impossible because each dataset had different effect ranges and dose-sampling densities, so the origin study was evident from the data.

Reporting for specific materials, systems and methods

We require information from authors about some types of materials, experimental systems and methods used in many studies. Here, indicate whether each material, system or method listed is relevant to your study. If you are not sure if a list item applies to your research, read the appropriate section before selecting a response.

Materials & experimental systems

n/a	Involved in the study
☒	☐ Antibodies
☒	☐ Eukaryotic cell lines
☒	☐ Palaeontology and archaeology
☒	☐ Animals and other organisms
☒	☐ Human research participants
☒	☐ Clinical data
☒	☐ Dual use research of concern

Methods

n/a	Involved in the study
☒	☐ ChIP-seq
☒	☐ Flow cytometry
☒	☐ MRI-based neuroimaging
